# Autophagy inhibition potentiates the antileukemic effect of FLT3 inhibitors and overcomes resistance in *FLT3*-ITD acute myeloid leukemia

**DOI:** 10.1038/s41420-026-03037-7

**Published:** 2026-03-24

**Authors:** Manuela Albuquerque de Melo, Brunno Gilberto Santos de Macedo, Diego A. Pereira-Martins, Antônio Bruno Alves-Silva, Natasha Peixoto Fonseca, Livia Bassani Lins de Miranda, Cleide Lucia Araújo Silva, Priscila Santos Scheucher, Jan Jacob Schuringa, João Agostinho Machado-Neto, Fabiola Traina

**Affiliations:** 1 Department of Medical Images, Hematology, and Oncology, University of São Paulo at Ribeirão Preto Medical School, Ribeirão Preto, SP Brazil; 2 Department of Experimental Hematology, Cancer Research Center Groningen, University Medical Center Groningen, Groningen, Netherlands; 3 Department of Pharmacology, Institute of Biomedical Sciences, University of São Paulo, São Paulo, SP Brazil; 4 Regional Blood Center of Ribeirão Preto, University of São Paulo at Ribeirão Preto Medical School, Ribeirão Preto, SP Brazil

**Keywords:** Translational research, Acute myeloid leukaemia, Cancer therapeutic resistance

## Abstract

Autophagy induction has recently emerged as a mechanism of resistance to FLT3 inhibitors (FLT3i) in patients with *FLT3*-ITD mutant acute myeloid leukemia (AML). Here, we assessed the molecular mechanisms of autophagy inhibition associated with FLT3i and its impact on cell survival and pharmacological resistance. In *FLT3*-ITD AML cell lines (MOLM13 and MV4-11), treatment with first- and second-generation FLT3i (midostaurin and quizartinib, respectively) induced autophagy. Combining FLT3i with autophagy inhibitors further decreased cell viability and increased cell apoptosis in both cell lines and in primary patient samples. Label-free quantification proteomics of MOLM13 cells revealed that RFC4 (Replication Factor C Subunit 4), an autophagy regulator linked to increased chemosensitivity, and GATD3/C21orf33 (Glutamine Amidotransferase Class 1 Domain Containing 3) proteins were upregulated only in the combined group, while 11 proteins mostly associated with chemoresistance were downregulated. In vivo, the combination of midostaurin and autophagy inhibition improved overall survival in MOLM13-transplanted mice. *ATG5*- (Autophagy Related 5) and *ATG7*-knockdown (Autophagy Related 7) increased sensitivity to first- and second-generation FLT3i in MOLM13 cells. To investigate the potential of autophagy inhibition in overcoming FLT3i resistance, we generated MV4-11 cells resistant to quizartinib (MV4-11QR). The resistant cell line exhibited higher basal levels of autophagy compared to the parental cell line. The combination of quizartinib and chloroquine demonstrated a synergistic effect in MV4-11QR cells and this effect was associated with greater inhibition of the FLT3 receptor compared to the monotherapies. Therefore, combining FLT3i with autophagy inhibition enhances the FLT3i antileukemic efficacy and overcomes pharmacological resistance.

## Introduction

Mutations in the FLT3 receptor are present in approximately one third of patients with acute myeloid leukemia (AML), with the *FLT3*-ITD mutation being the most prevalent and often associated with poor prognosis [1]. The development of midostaurin and quizartinib, a first- and second-generation FLT3 inhibitors (FLT3i), represented a major advancement in *FLT3*-ITD AML treatment [2–4]. Although FLT3i provide improved survival in AML patients, clinical limitations represent an opportunity to investigate possible acquired mechanisms of drug resistance. Autophagy has been implicated in resistance to second-generation FLT3i and is emerging as a potential therapeutic target in AML [5–9].

Autophagy is a cellular mechanism that involves lysosomal degradation and recycling of senescent and/or damaged intracellular components, favoring cellular homeostasis, maintaining cellular vitality and promoting a cytoprotective effect when cells are under conditions of intracellular infections, metabolic stress and, especially, therapeutic stress [10]. However, in cells that are unable to die by apoptosis, autophagy can also be considered a cell death process, as, in this context, it can mediate apoptosis or even induce cell death itself [10]. We therefore hypothesize that first- and second-generation FLT3i are capable of inducing autophagy as a mechanism of drug resistance, and that the inhibition of autophagy can reverse resistance and enhance the antineoplastic effect of FLT3i.

We herein compared the cellular and molecular effects of first- and second-generation FLT3i as monotherapy or in combination with autophagy inhibitors in human and murine *FLT3*-ITD AML models, including a FLT3i-resistant model. Our findings demonstrated that FLT3i treatment triggers autophagic flux, restricting the inhibitory activity of the drug, and that the antileukemic efficacy of FLT3i was enhanced by autophagy, overcoming cellular resistance.

## Results

### First- and second-generation FLT3 inhibitors induce autophagy as a mechanism that reduces their efficacy by attenuating the PI3K/AKT/mTOR pathway

**Fig. 1:**
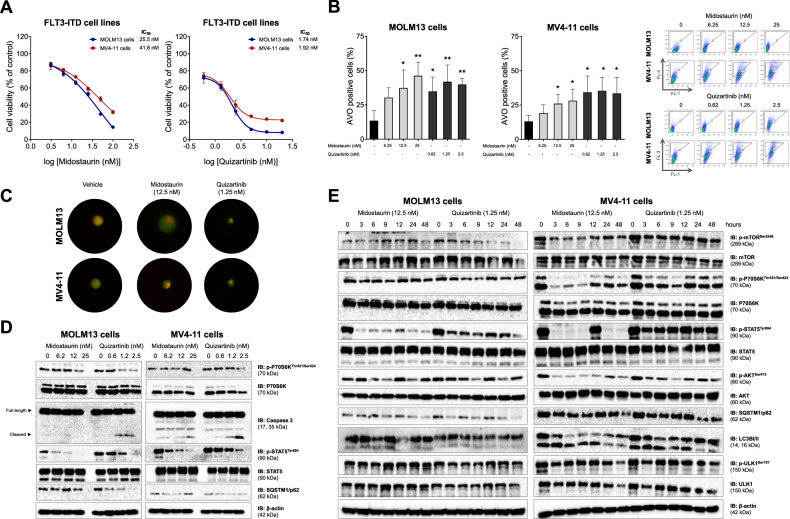
FLT3 inhibitors induce autophagy in *FLT3*-ITD models. **A** Dose-response curve for midostaurin and quizartinib treatment in MOLM13 and MV4-11 cells. Analysis was performed by methylthiazoletetrazolium (MTT) assay for MOLM13 cells (blue) and MV4-11 cells (red) after 48 h of treatment with vehicle, midostaurin (3.125, 6.25, 12.5, 25, 50, and 100 nM) or quizartinib (0.625, 1.25, 2.5, 5, 10, and 20 nM). Values are expressed as the percentage of viable cells for each condition relative to untreated controls. Data are presented as the mean of at least three independent experiments. In MOLM13 cells, the median inhibitory concentration (IC_50_) of midostaurin was 25.5 nM, whereas in MV4-11 cells, the IC_50_ was 41.8 nM. The IC_50_ of quizartinib was 1.8 nM in MOLM13 cells and 2.327 nM in MV4-11 cells. **B** Treatment with FLT3i increased the frequency of acidic vesicular organelles (AVO) detected by flow cytometry in MOLM13 and MV4-11 cells treated with vehicle or graded concentrations of midostaurin (6.25, 12.5, and 25 nM) or quizartinib (0.62, 1.25, and 2.5 nM) for 48 h using the acridine orange staining method. Representative dot plots are shown for each condition; the “AVO” gate contains cells positive for the FL-3 channel, which is characterized by increased acidic vesicular organelle formation and acridine orange color shift. Bar graphs represent the mean and standard deviation of at least three independent experiments; *p* values are indicated on the graphs. *p < 0.05; **p < 0.01, for cells treated compared with vehicle; ANOVA test and Bonferroni post-test. **C** Treatment with FLT3i increased the formation of autophagosomes in MOLM13 and MV4-11 cells, visualized by the color change of acridine orange (green to orange) upon pH changes during the formation of acidic autophagic organelles. Slides were prepared after 48 h of treatment with vehicle, midostaurin (12.5 nM) or quizartinib (1.25 nM), and after 15 min of incubation with the acridine orange probe. **D** Analysis of the dose-dependent effect of midostaurin and quizartinib on the protein expression of MOLM13 and MV4-11 cells. Western blotting analysis was performed on total cell extracts of cells treated with vehicle, midostaurin (6.25, 12.5, and 25 nM) or quizartinib (0.62, 1.25, and 2.5 nM) for 48 h. **E** Analysis of the effect of midostaurin and quizartinib over time on protein expression of MOLM13 and MV4-11 cells. Western blotting analysis was performed on total cell extracts of cells treated with vehicle, midostaurin (12.5 nM) or quizartinib (1.25 nM) for 0, 3, 6, 9, 12, 24, and 48 h.

To evaluate whether first- and second-generation FLT3i induce autophagy, we assessed the formation of AVOs in *FLT3*-ITD-positive MOLM13 and MV4-11 AML cell lines treated with midostaurin (6.25, 12.5, and 25 nM) or quizartinib (0.62, 1.25, and 2.5 nM) for 48 h. The selected doses were based on the dose-response curve for each drug (Fig. 1A). Treatment with both FLT3i significantly increased AVO formation (Fig. 1B, C).

Analysis of the dose- and time-dependent effects of midostaurin and quizartinib on protein expression and activation revealed a reduction in the phosphorylation of key downstream FLT3-related targets, including STAT5, mTOR, AKT, and P70S6K. The increase in autophagic flux following treatment with pharmacological FLT3i was also evident, as indicated by the decrease in p-ULK1 levels at its inhibitory site (Ser757), the conversion of LC3B-I to LC3B-II, and the consumption of SQSTM1/p62 and LC3B-II proteins. The conversion of LC3B-I to LC3B-II began after 3 h and was observed predominantly between 6 and 12 h, followed by degradation of LC3B-II after 24 and 48 h. The most pronounced reduction in SQSTM1/p62 was observed between 24 and 48 h (Fig. 1D, E, and Supplementary Fig. S1A, B). These findings suggest an initial period of autophagic induction followed by flux progression.

### Pharmacological inhibition of autophagy potentiates the antileukemic effect of FLT3 inhibitors

**Fig. 2:**
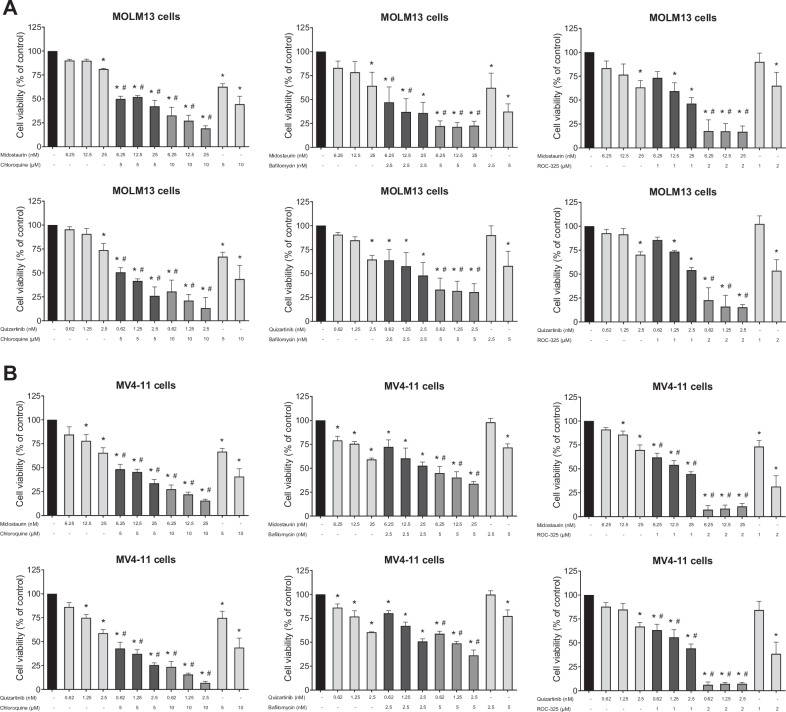
Combining FLT3 inhibitors with pharmacological inhibition of autophagy reduces cell viability in vitro compared with monotherapies. Cell viability after treatment with vehicle, midostaurin (6.25, 12.5, and 25 nM) or quizartinib (0.62, 1.25, and 2.5 nM) alone or combined with chloroquine (5 and 10 μM), bafilomycin A1 (2.5 and 5 nM), or ROC-325 (1 and 2 μM) in MOLM13 (**A**) and in MV4-11 (**B**) for 48 h. Values expressed represent the percentage of viable cells for each condition relative to the control. Bar graphs represent the mean and standard deviation of at least three independent experiments; *p* values are indicated in the graphs; *p < 0.05 for cells treated with FLT3i or autophagy inhibitor compared to vehicle; #*p* < 0.05 for cells treated with the combination of FLT3i and autophagy inhibitor compared to FLT3i alone at the corresponding dose; ANOVA and Bonferroni post-test.

To assess the impact of combining first- and second-generation FLT3i with autophagy inhibitors, we treated MOLM13 and MV4-11 cell lines for 48 h with midostaurin (6.25, 12.5, and 25 nM) or quizartinib (0.62, 1.25, and 2.5 nM) in combination with the autophagy inhibitors chloroquine (5 and 10 μM), bafilomycin A1 (2.5 and 5 nM), or ROC-325 (1 and 2 μM). The combination of FLT3i with autophagy inhibitors significantly reduced cell viability and cell counts compared to FLT3i monotherapy at the corresponding doses (Fig. 2A, B, and Supplementary Figs. S2, S3A).

**Fig. 3:**
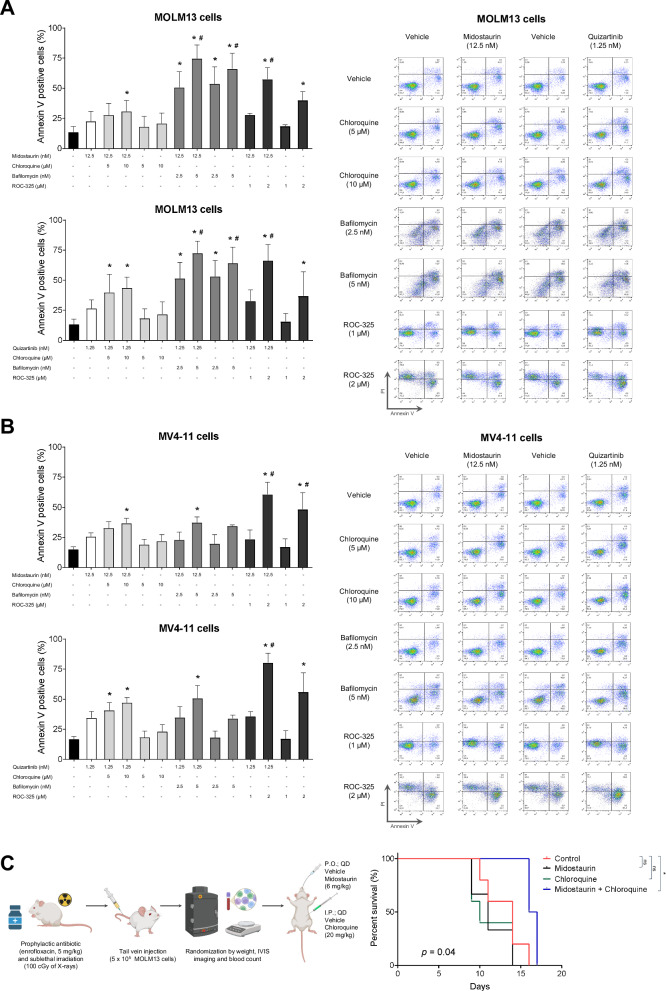
Combining FLT3 inhibitors with autophagy inhibitors increases cell apoptosis in vitro and improves overall survival in vivo of FLT3-ITD mice model. Apoptosis was detected by flow cytometry in MOLM13 cells (**A**) and in MV4-11 cells (**B**) treated with vehicle, midostaurin (12.5 nM) or quizartinib (1.25 nM) alone or combined with chloroquine (5 and 10 μM), bafilomycin A1 (2.5 and 5 nM), or ROC-325 (1 and 2 μM) for 48 h; the staining method used was annexin V/PI. Bar graphs represent the mean and standard deviation of at least three independent experiments; *p* values are indicated in the graphs; *p < 0.05 for cells treated with FLT3i or autophagy inhibitor compared with vehicle; #*p* < 0.05 for cells treated with the combination of FLT3i and autophagy inhibitor compared with FLT3i treatment alone at the corresponding dose; ANOVA and Bonferroni post-test. Representative dot plots are shown for each condition; the upper and lower right quadrants (Q2 and Q3) contain cumulatively the apoptotic population (annexin V+ cells). **C** Design of the in vivo experiment in a *FLT3*-ITD AML xenograft murine model. A single dose of the MOLM13 cell line (5 × 10^5^ cells) was transplanted by tail vein injection into female NSG (NOD.Cg-Prkdc scid IL2Rg Tm1Wjl Tg) mice to induce an AML xenograft model. The animals were previously treated with prophylactic antibiotics for 5 days and previously irradiated with a dose of 100 cGy of X-rays. After 7 days of xenograft, the animals underwent imaging and blood count and the experimental groups were randomized according to weight, chimerism and blood count. The four experimental groups were treated daily with vehicle, midostaurin (6 mg/kg, oral), chloroquine (20 mg/kg, intraperitoneal), or combined treatment until death. Figure were created with Biorrender.com software. Combined treatment of midostaurin with chloroquine increased overall survival in *FLT3*-ITD AML murine model in vivo. Survival analysis was performed using the Kaplan–Meier method and Gehan–Breslow–Wilcoxon test.

Increased apoptosis was observed in MOLM13 and MV4-11 cell lines following 48-hour treatment with midostaurin (12.5 nM) or quizartinib (1.25 nM) in combination with chloroquine (5 and 10 μM), bafilomycin A1 (2.5 and 5 nM), or ROC-325 (1 and 2 μM) (Fig. 3A, B). The ex vivo combined treatment with midostaurin (12.5 nM) or quizartinib (1.25 nM) and chloroquine (5 μM) significantly reduced cell viability and induced apoptosis in the *FLT3*-ITD AML sample, whereas no effect was observed in the *FLT3*-WT AML sample (Supplementary Fig. S4).

An in vivo xenograft mouse model using MOLM13 cells (*n* = 5 per group) was established. The combination of low-dose FLT3i midostaurin (6 mg/kg/day) and chloroquine (20 mg/kg/day), significantly improved overall survival and preserved hemoglobin levels in the transplanted mice (Fig. 3C and Supplementary Table S5).

### Genetic inhibition of autophagy potentiates the antileukemic effect of FLT3 inhibitors

**Fig. 4:**
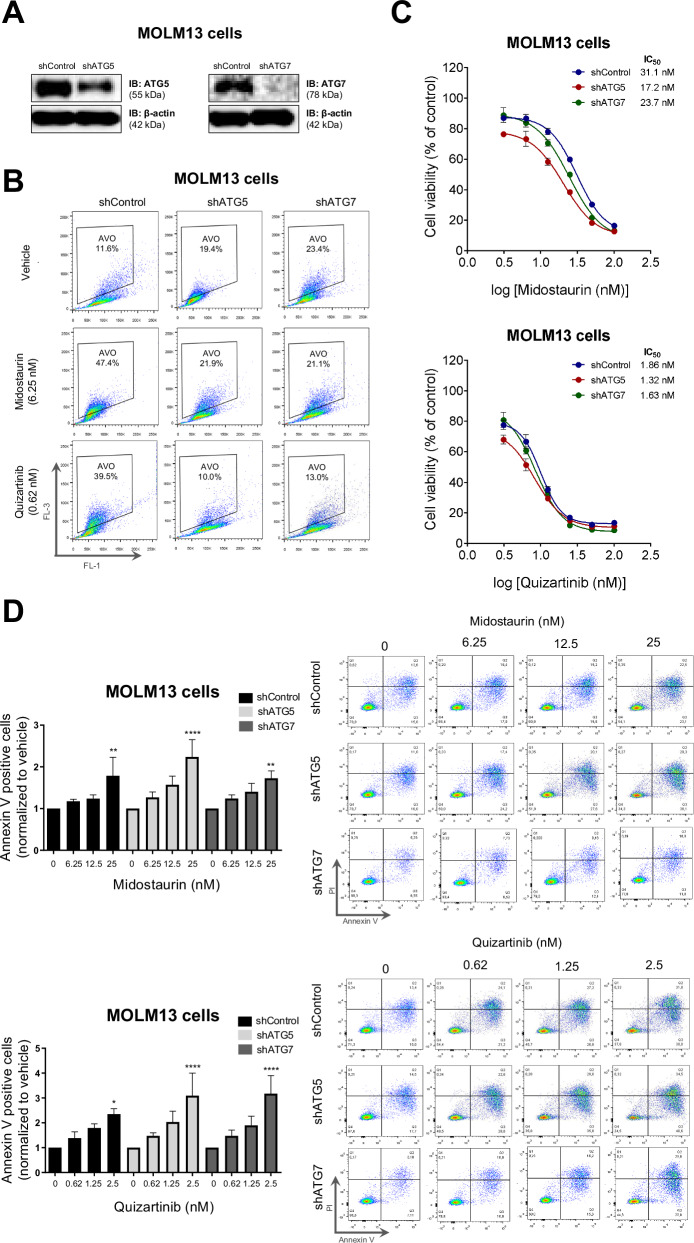
Combining FLT3 inhibitors with genetic inhibition of autophagy reduces cell viability and increases apoptosis in vitro. **A** Analysis of protein expression of ATG5 and ATG7 in MOLM13 cells after gene silencing. Cells were named as: “shControl”, non-silencing control; “shATG5”, *ATG5*-silenced cells; “shATG7”, *ATG7*-silenced cells. **B** Treatment with FLT3i was unable to induce autophagy in MOLM13 cells after *ATG5* and *ATG7* gene silencing. The frequency of acidic vesicular organelles was detected by flow cytometry in MOLM13 cells (shCTRL, shATG5, and shATG7) treated with vehicle, midostaurin (6.25 nM) or quizartinib (0.62 nM) for 48 h using the acridine orange staining method. Representative dot plots are shown for each condition; the “AVO” gate contains cells positive in the FL-3 channel, which is characterized by increased acidic vesicular organelle formation and acridine orange chromatic shift. **C** Dose-response curve for midostaurin and quizartinib treatment after genetic inhibition of autophagy in MOLM13 cells. Analysis was performed by MTT assay for MOLM13 cells silenced for *ATG5* (red), for *ATG7* (black) or for the non-silenced control (blue) after 48 h of treatment with vehicle, midostaurin (3.125, 6.25, 12.5, 25, 50, and 100 nM) or quizartinib (0.62, 1.25, 2.5, 5, 10, and 20 nM). Values are expressed as the percentage of viable cells for each condition relative to untreated controls. Data are presented as the mean of at least three independent experiments. In non-silenced MOLM13 cells, the IC_50_ of midostaurin was 31.03 nM, whereas in *ATG5* and *ATG7*-silenced cells, the IC_50_ was reduced to 17.16 nM and 23.66 nM, respectively. The IC_50_ of quizartinib was 1.857 nM in shCTRL cells, 1.327 nM in shATG5 cells, and 1.623 nM in shATG7 cells. **D** Apoptosis was detected by flow cytometry in *ATG7* knockdown (shATG7), *ATG5* knockdown (shATG5) or non-knockdown (shCTRL) MOLM13 cells treated with vehicle, midostaurin (6.25, 12.5, and 25 nM) or quizartinib (0.62, 1.25, and 2.5 nM) for 48 h at the indicated doses; the staining method used was annexin V/PI. Bar graphs represent the mean and standard deviation of at least three independent experiments; *p* values are indicated in the graphs. *p < 0.05, **p < 0.01, ****p < 0.0001, compared with vehicle; ANOVA test and Bonferroni post-test. Representative dot plots are shown for each condition; the upper and lower right quadrants (Q2 and Q3) cumulatively contain the apoptotic population (annexin V+ cells).

To provide complementary evidence supporting the role of autophagy in the mechanistic effect of FLT3i, we performed a knockdown (KD) of the autophagy-essential genes, *ATG5* and *ATG7*, using shRNA in the MOLM13 cell line (Fig. 4A). The induction of autophagy following 48-hour treatment with midostaurin and quizartinib was abolished after *ATG5*-KD and *ATG7*-KD (Fig. 4B). Genetic inhibition of autophagy, through *ATG5* and *ATG7* silencing, combined with FLT3i, significantly enhanced the efficacy of FLT3i in *FLT3*-ITD cells, as evidenced by reduced cell counts compared to FLT3i monotherapy at the corresponding dose in MOLM13 (Supplementary Fig. S3B). *ATG5*-KD and *ATG7*-KD combined with FLT3i were associated with lower IC50 values, reduced cell counts, and increased apoptosis for both first- and second-generation FLT3i in MOLM13 and MV4-11, compared to controls (Fig. 4C, D).

### FLT3 inhibitors-induced autophagy enhances autophagy inhibitors effect

Protein molecular analysis of the combination of FLT3i (both first- and second-generation) and autophagy inhibitors was conducted in the MOLM13 cell line. FLT3i monotherapy (midostaurin at 12.5 nM or quizartinib at 1.25 nM) increased ATG7 expression, a protein essential for the initiation of the autophagic process.

**Fig. 5:**
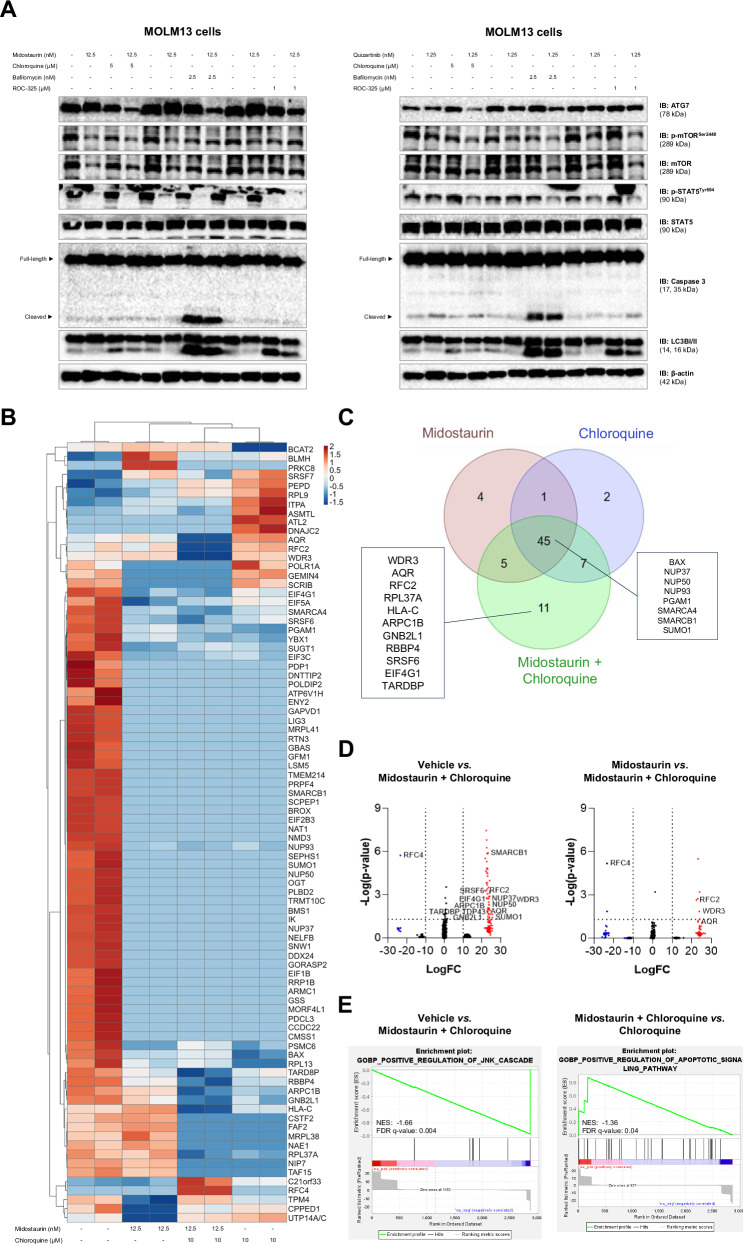
Protein modulation of combined treatment between FLT3 inhibitors and autophagy inhibitors. **A** Analysis of the effect of midostaurin and quizartinib combined or not with chloroquine, bafilomycin A1 or ROC-325 in the MOLM13 cell line. Western blotting analysis was performed on total cell extracts from cells treated with vehicle, midostaurin (12 nM) or quizartinib (1.2 nM) alone or combined with chloroquine (5 μM), bafilomycin A1 (2.5 nM) or ROC-325 (1 μM) for 48 h. **B** Heatmap for the protein expression analysis in MOLM13 cells treated with vehicle, midostaurin (12.5 nM), chloroquine (10 µM) and combination for 48 h. The data represent the fold change of the vehicle-treated cells, and the downregulated and upregulated proteins are shown in blue and red, respectively. **C** Venn diagram analysis of downregulated proteins expressed in midostaurin monotherapy, chloroquine monotherapy and combination. **D** Volcano plot demonstrates the differentially expressed proteins comparing vehicle *vs*. combination and monotherapy with midostaurin *vs*. combination. **E** GSEA of the MOLM13 proteome comparing vehicle *vs*. combination and combination *vs*. monotherapy with chloroquine.

In contrast, the combination of FLT3i with autophagy inhibitors (chloroquine at 5 μM, bafilomycin A1 at 2.5 nM, or ROC-325 at 1 μM) further reduced ATG7 expression compared to the autophagy inhibitor alone, indicating that the activity of autophagy inhibitors is dependent on autophagic flux. Autophagy inhibition was also demonstrated by the accumulation of LC3B-II. Importantly, the combined treatment did not affect the efficacy of FLT3i in inhibiting p-mTOR^Ser2448^ and p-STAT5^Tyr694^; in fact, it appeared to reduce these markers more effectively than FLT3i monotherapy (Fig. 5A, and Supplementary Fig. S1D, E).

To identify novel targets associated with the response to the combined treatment of FLT3i and chloroquine, we performed label-free quantification proteomics on MOLM13 cells treated with vehicle, midostaurin (12.5 nM), chloroquine (5 μM), and combination. A total of 86 proteins were differentially expressed among the four conditions (Fig. 5B). Notably, 45 proteins were downregulated across all treatment conditions (midostaurin, chloroquine, and combination), many of which are involved in cell proliferation and survival (e.g., BAX, SMARCA4, and SUMO1), suggesting common pathways of action between both drugs (Fig. 5C). RFC4, an autophagy regulator associated with increased chemosensitivity, and GATD3/C21orf33, a mitochondrial protein, were upregulated exclusively in the combination treatment group (Fig. 5B, D). Eleven proteins, including RBBP4, EIF4G, WDR3, AQR, RFC2, ARPC1B, GNB2L1/RACK1, SRSF6, and TDP43, were downregulated only in the combined treatment group, all of which are linked to chemotherapy resistance and poor prognosis in AML *FLT3*-ITD models and other cancer models (Fig. 5B, D). Gene set enrichment analysis (GSEA) revealed molecular signatures associated with cell death in the combination treatment group, including the “positive regulation of JNK cascade” compared to vehicle and the “positive regulation of apoptotic signaling pathway” compared to chloroquine (Fig. 5E).

### Chloroquine reverses pharmacological resistance in a cell line model resistant to quizartinib

**Fig. 6:**
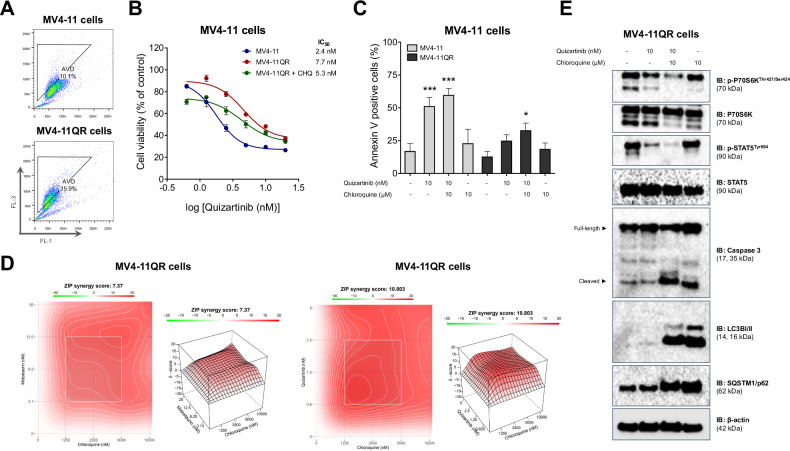
Quizartinib-resistant cell line has high basal autophagy, which, when inhibited by chloroquine, increases cell death. **A** Acquisition of quizartinib resistance increased the frequency of acidic vesicular organelles detected by flow cytometry using the acridine orange staining method in MV4-11QR cells compared with nonresistant MV4-11 cells. Representative dot plots are shown for each condition; the “AVO” gate contains cells positive for the FL-3 channel, which is characterized by increased acidic vesicular organelle formation and acridine orange color shift. **B** Dose-response curve for quizartinib treatment in nonresistant MV4-11 (black), MV4-11QR (blue), and MV4-11QR cells treated with 10 μM chloroquine (red). Analysis was performed by MTT assay after 48 h of treatment with vehicle or quizartinib (0.625, 1.25, 2.5, 5, 10, and 20 nM). Values are expressed as the percentage of viable cells for each condition relative to untreated controls. Data are presented as the mean of at least three independent experiments. In nonresistant MV4-11 cells, the IC_50_ of quizartinib was 2.2 nM, whereas in MV4-11QR cells it was 7.7 nM, and in chloroquine-treated MV4-11QR cells the IC_50_ was reduced to 5.3 nM. **C** Chloroquine treatment increased quizartinib-induced apoptosis in both nonresistant MV4-11 and MV4-11QR cell lines. Apoptosis was detected by flow cytometry in cells treated with vehicle, quizartinib (10 nM), chloroquine (10 μM), or combination for 48 h; the staining method used was annexin V/PI. Bar graphs represent the mean and standard deviation of at least three independent experiments; *p* values indicated in the graphs. *p < 0.05, and ***p < 0.001 for cells treated compared with vehicle; ANOVA test and Bonferroni post-test. **D** Determination of the degree of synergy between midostaurin and chloroquine and between quizartinib and chloroquine in MV4-11QR cells. Dose-response cytotoxicity of the combined treatment was performed by the MTT assay after 48 h of treatment with vehicle, midostaurin (3.1, 6.2, 12.5, and 25 nM) or quizartinib (0.6, 1.2, 2.5, and 5 nM) combined with chloroquine (1.2, 2.5, 5, and 10 μM). Values are expressed as the percentage of viable cells for each condition relative to untreated controls. The ZIP score was determined using the software SynergyFinder (https://synergyfinder.fimm.fi/). **E** Analysis of the effect of quizartinib combined or not with chloroquine on protein expression of MV4-11QR cells. Western blotting analysis was performed on total cell extracts from cells treated with vehicle, quizartinib (10 nM), chloroquine (10 μM), or combination for 48 h.

The MV4-11QR cells exhibited an increase in basal autophagic flux, as evidenced by the elevated quantification of AVOs (Fig. 6A) and enhanced consumption and conversion of the LC3B-I protein (Fig. 6E).

The combination treatment of quizartinib and chloroquine reduced the IC_50_ of quizartinib (7.7 nM vs. 5.3 nM) (Fig. 6B) and increased the proportion of cells undergoing apoptosis (Fig. 6C). Chloroquine showed an additive effect with midostaurin (ZIP score = 7.37) and a synergistic effect with quizartinib (ZIP score = 10.8), in MV4-11QR cells (Fig. 6D). The ZIP score for chloroquine combined with quizartinib was higher in the resistant MV4-11QR lineage compared to the non-resistant lineage (Supplementary Fig. S5), suggesting that the antineoplastic effect of chloroquine was more evident in the quizartinib-resistant cellular model, which exhibited increased basal autophagic flux.

Treatment with chloroquine effectively reversed autophagy, as indicated by the accumulation of LC3B-II and SQSTM1/p62 proteins. This, in turn, enhanced the inhibitory effect of quizartinib on the FLT3 receptor, demonstrated by a greater reduction in the phosphorylation of STAT5^Tyr694^ and P70S6K^Thr421/Ser424^. Consequently, this led to an increase in cell death, marked by elevated levels of cleaved Caspase 3, compared to quizartinib monotherapy. Compared to the combined treatment, autophagy inhibitor monotherapy showed higher LC3B-I and SQSTM1/p62 accumulation, a fact that is consistent with the finding that FLT3i-induced autophagy through LC3B consumption (Fig. 6E, Supplementary Figs. S1F, S6).

## Discussion

We observed that both first- and second-generation FLT3i induced autophagy in *FLT3*-ITD AML cell lines. Inhibition of autophagy enhanced FLT3i antineoplastic effect and overcame pharmacological resistance. FLT3i-induced autophagy activation represents a therapeutic opportunity to combine autophagy inhibitors with FLT3i, particularly in cases of pharmacologic resistance to FLT3 inhibition, as autophagy is a key mechanism of resistance.

In a previous study, *FLT3*-ITD expression in the MOLM13 cell line was associated with increased basal autophagy, indicating the association between *FLT3*-ITD mutation and autophagy induction in AML cells [5]. Through analyses of autophagy proteins expression, we identified that *FLT3*-ITD mutation in MOLM13 and MV4-11 cells indeed led to an increase in basal autophagy, which can be explained by the high mitochondrial oxidative phosphorylation present in these cells, which, in turn, activates autophagy, and may contribute to AML tumorigenesis [11–13].

Through in silico analysis, our group previously demonstrated that AML patients who respond poorly to ex vivo treatment with FLT3i have enriched autophagy-related pathways [8]. This observation indicates that *FLT3*-ITD AML samples that were inherently more resistant to FLT3i had higher expression of genes associated with autophagy [8]. Wang et al. identified autophagy activation as a potential target in chemotherapy AML resistant patients by analyzing ex vivo drug response and public multi-omics data [14].

Herein, we demonstrated that genetic or pharmacological inhibition of autophagy induced by midostaurin and quizartinib increased cell sensitivity to FLT3i in MOLM13 and/or MV4-11 cell lines. Furthermore, chloroquine and hydroxychloroquine are among the most extensively studied autophagy inhibitors in preclinical and clinical settings [15]. We demonstrated that the combined treatment with midostaurin and chloroquine was effective in *FLT3*-ITD AML models, in vitro, ex vivo and in vivo.

We demonstrate that the combination of midostaurin and chloroquine increased overall survival and maintained stable hemoglobin levels, even at low FLT3i dose, in a highly aggressive murine model of FLT3-ITD AML transplanted with MOLM13 cells. These findings are consistent with previous in vivo observations reported by Koshade et al., who showed that combined treatment with the FLT3i gilteritinib and the autophagy inhibitor ROC-325 improved survival in mice xenografted with MV4-11 cells [6]. These results suggest that the antileukemic efficacy of FLT3i may be enhanced by pharmacological inhibition of autophagy across distinct *FLT3*-ITD AML models, encompassing both more aggressive and less aggressive disease settings. Our data further indicate that chloroquine, a low-cost and widely accessible drug, may represent a feasible strategy to pharmacologically modulate autophagy and improve therapeutic responses to FLT3 inhibition in AML.

Koshade et al. also demonstrated, using cell counting assays, that *ATG3*-KD or pharmacological inhibition of autophagy with ROC-325 acts synergistically with second-generation FLT3i in vitro [6]. In agreement, the present study showed that genetic inhibition of autophagy through *ATG5* or *ATG7* silencing, as well as pharmacological autophagy inhibition using chloroquine, bafilomycin, or ROC-325, was associated with enhanced antineoplastic effects of midostaurin and quizartinib in vitro. The magnitude of the response was more robust when evaluated by cell counting and MTT assays than when assessed by annexin V–based apoptosis assays. This difference is related to methodological aspects and to the distinct biological processes measured by each assay. While cell counting and MTT assays capture cumulative effects on cell proliferation, metabolic activity, and viability over time, annexin V staining specifically detects early apoptotic events within a defined time window. Autophagy inhibition may initially impair cellular fitness, proliferation, or metabolic adaptation before committing cells to apoptotic death, which could result in attenuated differences when apoptosis is assessed as a single endpoint. These considerations support the complementary use of multiple methodological approaches to assess treatment response.

FLT3i as monotherapy increased ATG7 protein expression, essential for the initiation of the autophagic process, and inhibited AKT^Ser473^, mTOR^Ser2448^ and ULK1^Ser757^ phosphorylation, promoting autophagy as a consequence. In agreement, Koschade et al. showed that, in MOLM14 and MV4-11 cells, second-generation FLT3i induced autophagy mediated by decreased phosphorylation of these same proteins [6]. We also observed that the combined treatment with FLT3i and autophagy inhibitors reduced ATG7, and more effectively reduced mTOR and p-p70S6K phosphorylation compared to the respective monotherapies. This finding suggests that autophagy inhibitors act more efficiently in situations of high autophagic flux induced by FLT3i and that autophagy inhibition increases the molecular effect of FLT3i, resulting in cell death.

By evaluating protein expressions following combined treatment with FLT3i and pharmacological autophagy inhibitors, a more pronounced reduction of ATG7 compared to the use of the autophagy inhibitor alone was observed. This finding indicates that autophagy is a relevant therapeutic target upon FLT3i, as *FLT3*-ITD–mutated cells become more sensitive to autophagy inhibition. The FLT3i and autophagy inhibitors combined treatment also reduced STAT5^Tyr694^ phosphorylation more effectively compared to monotherapy with FLT3i, indicating that autophagy inhibitors increase the therapeutic efficacy of FLT3i not only by inhibiting the cellular resistance mechanism (autophagy), but also by favoring the inhibition of the FLT3 receptor by FLT3i. Proteomic analysis revealed that pathways related to cell proliferation and survival were downregulated in combined treatment with midostaurin and chloroquine and in monotherapy for both. One of the proteins significantly upregulated only in the combined treatment was GATD3/C21orf33, a mitochondrial deglycase responsible for maintaining mitochondrial integrity by removing advanced glycation end products [16]. Another protein also upregulated in the combined treatment was RFC4, a DNA replication factor recently associated with increased sensitivity to Cytarabine in AML model [17]. Among the proteins downregulated only in the combined treatment, two have already been studied in the context of AML, RBBP4 and EIF4G. RBBP4 knockdown was able to increase the sensitivity of AML cells to Histone Deacetylase inhibitors [18]. Increased formation of the EIF4E-EIF4G complex was associated with STAT5 activation and resistance to PI3K-AKT inhibitors in *FLT3*-ITD AML [19]. Other proteins also downregulated in the combined treatment have already been studied in other types of cancer and associated with tumor phenotype and/or prognosis: WDR3, RFC2, ARPC1B, GNB2L1/RACK1, SRSF6 and TDP43 [20–28].

The MV4-11QR cells, a quizartinib-resistant cell model, showed higher basal autophagy compared to the nonresistant MV4-11 cell line, and combined treatment between midostaurin or quizartinib and chloroquine had an even greater synergy in the resistant cell, confirming that autophagy can be considered a mechanism of acquired resistance to FLT3i in the *FLT3*-ITD AML model and that inhibition of autophagy indeed reverses drug resistance and increases the efficacy of first- or second-generation FLT3i. The cellular resistance model allowed us to validate our findings that chloroquine is autophagy-dependent, demonstrating a potentiated effect under conditions of resistance to the FLT3i. The accumulation of LC3B-II and SQSTM1/p62 proteins following the combined treatment indicates the inhibition of FLT3i-induced autophagy and the increased basal autophagy associated with acquired drug resistance. The combined treatment more effectively reduced p70S6K and STAT5 phosphorylation, enhancing the molecular efficacy of the FLT3i, and promoting cell death, as evidenced by increased cleaved Cas3 levels.

This study demonstrates that both first- and second-generation FLT3i induce autophagy, and the resulting increase in autophagic flux limits the therapeutic efficacy of FLT3i. Inhibition of autophagy enhances the antineoplastic effect of midostaurin and quizartinib, showing a greater synergistic effect in a model of FLT3i resistance. These findings highlight that FLT3i-induced autophagy activation represents a therapeutic opportunity, providing valuable insights into the mechanisms of FLT3i resistance. The analysis of autophagic profiles in AML may play a crucial role in diagnostic processes and the development of personalized treatment strategies.

## Material and methods

### Ethical statement

This research followed all ethical protocols and was approved by the Ethics Committee on the Use of Animals of the University of São Paulo at Ribeirão Preto Medical School and by the Research Ethics Committee of Clinical Hospital at University of São Paulo at Ribeirão Preto Medical School.

The reagents and equipment used are indicated in Supplementary Tables S1, S2, S3.

### Cell lines and cell culture

The MOLM13 and the MV4-11 cell lines were obtained from DSMZ and ATCC; respectively. The quizartinib-resistant MV4-11 cell line (MV4-11QR) was provided by Prof. Dr. João Agostinho Machado-Neto (University of São Paulo, São Paulo, Brazil). The induction of resistance to quizartinib in the MV4-11 cell line was carried out through intermittent drug exposure, selecting the cells that survived and gradually increasing the dose. Cells were tested and authenticated by Short Tandem Repeat (STR) matching analysis using the PowerPlex® 16 HS system and the ABI 3500 Sequence Detector System. RPMI 1640 culture medium containing 1% antibiotics penicillin and streptomycin 10,000 U/mL and 10% fetal bovine serum (FBS) was used in the cultivation of both strains. Cells were cultured at 37 °C with 5% carbon dioxide (CO_2_) in a humidified incubator and confirmed to be free of mycoplasma. The cells were used for a period of a maximum of 3 months, and the passages were carried out in accordance with the manufacturer. In all experiments, a confluence of 2 × 10^5^ cells/mL was used.

### Pharmacological inhibitors

The multi-targeted protein kinase inhibitors midostaurin (PKC412), and the type II FLT3i quizartinib (AC220), and the autophagy inhibitors ROC-325, bafilomycin A1 and chloroquine were diluted according to the manufacturer, in dimethyl sulfoxide (DMSO), distilled water or culture medium.

### Gene knockdown by short hairpin RNA (shRNA)

MOLM13 cells were transduced with lentivirus-mediated shRNA nonspecific control (shControl) or lentivirus-mediated shRNA targeting the *ATG5* (Autophagy Related 5 gene) (shATG5) or *ATG7* (Autophagy Related 7 gene) (shATG7). Briefly, 5 × 10^4^ cells were transduced with lentivirus by spinoculation at the multiplicity of infection equal to 4 and selected by Puromycin at 1 μg/mL.

### Cell viability assessment

Cell lines silenced or not for *ATG5* or *ATG7* and primary cells of patients with AML were subjected to cell viability assay both to determine half-maximal inhibitory concentration (IC_50_) and to verify the effect of treatment with FLT3i combined or not with autophagy inhibition. The cells were cultured in 96-well plates at 2 × 10^5^ cells/mL (20,000/well) and subjected to treatment with inhibitor drugs. After incubation for 48 h at 37 °C, 5% CO_2_, 50 μg of methylthiazol tetrazolium (MTT) reagent was added, and the cells were incubated again at 37 °C, 5% CO_2_ for 4 h. The reaction was stopped by the addition of 100 μL of hydrochloric acid-isopropanol solution in each well. Viability was assessed by measuring absorbance at 570 nm, using an automatic plate reader. Cell viability following combined treatment with FLT3i and genetic or pharmacological inhibition of autophagy was also assessed by cell counting using optical microscopy. Each condition was tested with at least three technical replicates and three biological replicates.

### Flow cytometry (FACS) for autophagy and apoptosis assessment

A 24-well plate was used, where 1 × 10^5^ cells were plated in each well in appropriate medium and treated with different concentrations of midostaurin and quizartinib. After 48 h, the cells were centrifuged at 450 × *g* for 5 min and resuspended in solution of acridine orange at 1 μg/mL in PBS buffer. The cells were incubated in the dark at room temperature for 15 min and the autophagic activity was determined by quantifying the ratio of green-to-red fluorescence, with the reading being carried out on the FL3 x FL1 channels, thus determining the number of acidic vesicular organelles (AVOs). To avoid false positive results, data acquisition and analysis was carried out in accordance with the recommendations published by Thomé and collaborators [29].

For cellular apoptosis assay, 1 × 10^5^ cells were plated in each well of a 24-well plate using appropriate culture medium and treated with FLT3i, in different concentrations, combined or not with autophagy inhibition, for 48 h in an oven at 37 °C, 5% CO_2_. The study of apoptosis was carried out by labeling cells with APC-annexin V and propidium iodide.

In all experiments, the mean fluorescence intensity of at least 10,000 events was obtained using the FACSCanto. Analyzes were performed with the FlowJo program. Each condition was tested with at least two technical replicates and three biological replicates.

### Fluorescence microscopy

To visualize the presence of the autophagic process in MOLM13 and MV4-11 cell lines, 1 × 10^5^ cells/well were plated into a 24-well plate and treated with midostaurin and quizartinib. After 48 h of treatment incubation at 37 °C, 5% CO_2_, cells were resuspended in acridine orange solution at 1 μg/mL buffer PBS and viewed under a microscope fluorescence. Each condition was tested with at least two technical replicates and three biological replicates.

### Western blot analysis

Equal amounts of protein were used as total extracts, followed by SDS-PAGE, Western blot analysis with the indicated antibodies and imaging using the SuperSignal™ West Dura Extended Duration Substrate System and Gel Doc XR+ system. The antibodies used are indicated in Supplementary Table S2. Detailed protocol is available in Supplementary Material. The protein acronyms are indicated in Supplementary Table S4. The bands were quantified using the UN-SCAN-IT software and the protein expression was normalized by the expression of the endogenous protein (Supplementary Fig. S1). Full and uncropped western blots are available (Supplemental Material - Full and uncropped western blots).

### Proteomics analysis

MOLM13 cells were exposed to vehicle, FLT3i (midostaurin, 12.5 nM), autophagy inhibitor (chloroquine, 10 μM) or with the combination of FLT3i plus autophagy inhibitor for 48 h. Proteomics analysis was carried out by the Proteomics/Mass Spectrometry Platform of the Carlos Chagas Institute of Fiocruz Paraná (ICC Fiocruz, Curitiba, Paraná, Brazil) using an Orbitrap Fusion Lumos System. All procedures were performed in accordance with the facility’s instructions. Detailed protocol is available in Supplementary Material. The protein acronyms are indicated in Supplementary Table S4.

### In vivo AML FLT3-ITD xenograft model

A single dose of cell line *FLT3*-ITD positive AML (MOLM13, 5 × 10^5^ cells) was transplanted through injection into the tail vein in animals anesthetized with 2% isoflurane, using a 30 G insulin needle, with a volume of 100 μL to induction of the AML xenotransplantation model. The animals’ tails were exposed to infrared light to heat and dilate the veins in the tail region. The mice used were female NSG (NOD.Cg-Prkdc scid IL2Rg Tm1Wjl Tg), at 12–16 weeks old, sublethally irradiated the day before with a dose of 100 cGy of X-rays and treated with prophylactic antibiotic enrofloxacin (5 mg/kg) for five days. One week after xenotransplantation, the animals were subjected to imaging on the IVIS Lumina Imaging System. The animals remained sedated with 1.5% isoflurane throughout the imaging. The experimental groups were randomized according to weight, degree of grafting and blood count. The four experimental groups consisted of animals treated once a day with vehicle, FLT3i (midostaurin, 6 mg/kg, orally), autophagy inhibitor (chloroquine, 20 mg/kg, intraperitoneally), and the combination of FLT3i and autophagy inhibitor – 5 animals per group; total of 20 animals. Twenty microliters of peripheral blood were collected from the facial vein (under anesthesia with 1.5% isoflurane) to evaluate the hematimetric count pre- and post-treatment. To assess survival, animals were monitored until death or were euthanized when presenting signs of terminal disease such as interruption of voluntary water and food consumption, hunched posture, bristly hair, lethargic state or reaching below 80% of its original weight.

### Ex vivo AML FLT3-ITD samples and treatment

Primary mononuclear cells from bone marrow were collected from 2 adult patients with diagnosis of AML, at the time of diagnosis (#1: absence of the *FLT3*-ITD variant; #2: presence of the *FLT3*-ITD variant). The mononuclear cell isolation of bone marrow was performed using gradient centrifugation using Histopaque®. The mononuclear cells were treated with FLT3i (midostaurin at 12.5 nM or quizartinib at 1.25 nM) associated or not with an autophagy inhibitor (chloroquine at 5 μM) to investigate the induction of autophagic process by FLT3i, as well as to evaluate the viability and cell death after combined treatment.

### Statistical analysis

Statistical analyses were performed using the GraphPad Prism software version 8 or by R software environment and programming language (RRID:SCR_001905). For comparisons, Student’s *t* tests or ANOVA tests were used. The Bonferroni post-test for correction was used when necessary. For mice survival, the Kaplan–Meier method with the Gehan–Breslow–Wilcoxon test and Benjamini–Hochberg Procedure were used to assess statistically significant differences in overall survival. The *p* values were adjusted for both sides with a significance level of 0.05.

## Supplementary information

Supplementary Materials

Full and Uncropped Western Blot

## Data Availability

The data generated in this study are available upon request from the corresponding author.

## Data availability

All datasets generated or analyzed during this study are included in the Materials and Methods section or in the Supplementary Materials. Additional data or information supporting the findings of this study are available from the corresponding author upon reasonable request.
