# Supplementary material for: Autophagy inhibition potentiates the antileukemic effect of FLT3 inhibitors and overcomes resistance in FLT3-ITD acute myeloid leukemia
Source: Cell Death Discov. 2026 Mar 24;12:174. doi: 10.1038/s41420-026-03037-7 (PMC13039817; doi:10.1038/s41420-026-03037-7)
Supplement: Supplementary file 1 — Supplementary Materials [file 41420_2026_3037_MOESM1_ESM.docx]

**SUPPLEMENTARY MATERIALS**

- Additional Material and Methods
- Supplementary Tables 1-5
- Supplementary Figures 1-6

**Additional Material and Methods**

**Detailed Western blot analysis**

The cell lines were plated in petri dishes in confluence of 2×10^5^ cells/mL and subjected to treatment with FLT3i drugs combined or not with autophagy inhibition, in different doses and times. After the determined time, the cells were then collected, centrifuged at 450 g for 5 minutes, resuspended in 1 mL of PBS buffer and centrifuged again at 700 g for 10 minutes at 4°C. The cellular precipitate containing between 5×10^6^ and 10×10^6^ cells was added protein extraction buffer containing 100 mM Tris (pH 7.6), 1% Triton X-100, 150 mM NaCl, 35 mg phenylmethylsulfonyl fluoride (PMSF)/mL, 10 mM Na_3_VO_4_, 100 mM NaF, 10 mM Na_4_P_2_O_7_, and 4 mM ethylenediaminetetraacetic acid (EDTA). The samples were homogenized and incubated for 60 to 120 minutes at 4°C. After this period, the samples were centrifuged at 12000 g, 4°C for 20 minutes. The protein extract was quantified based on Bradford method (Cat. No.: 5000006) (BioRad). Laemmli buffer was added to the total protein extract product containing 100 mmol/L dithiothreitol (DTT), 10% sodium dodecyl sulfate (SDS) and sodium blue bromophenol. The samples were stored at -80°C to carry out the Western Blot assay. The protein extract (15 μg) was subjected to electrophoresis in 8 to 15% polyacrylamide gel in an electrophoresis apparatus (Mini-Protean, Bio-Rad Laboratories). An electrotransfer of proteins from the gel to the nitrocellulose membrane was carried out for 120 minutes at 120V in a miniaturized BiotransferRad. Binding of antibodies to non-specific proteins was reduced by membrane incubation for 1 hour with a blocking buffer (5% dry milk sugar-free skim, 10 mM Tris, 150 mM NaCl, and 0.02% Tween 20) at room temperature. The nitrocellulose membrane was then incubated overnight with primary antibodies specific for the protein of interest diluted in buffer blocking (3% unsweetened skim milk powder, 10 mM Tris, 150 mM NaCl, and 0.02% Tween 20) for 12 hours at 4°C under agitation and then washed 3 times with basal solution (10 mM Tris, 150 mM NaCl, and 0.02% Tween 20). After that, the membrane was incubated for 2 hours with a secondary antibody specific to the antibody primary and conjugated to peroxidase. The development system used was based on chemiluminescence, using the SuperSignal West Dura Extended Duration kit Substrate System (Cat. No.: 34075) (Thermo Fisher Scientific) and Gel Doc XR+ system (Bio-Rad). The antibodies used are indicated in Supplementary Table 1. The intensity of expression protein was normalized by the expression of the endogenous protein actin, and the intensity of post-translational modifications, through the intensity of the total protein band corresponding. The final images were created from the portions of the gel that contains the bands of interest.

**Detailed proteomics analysis**

For total protein extraction, we collected MOLM13 cells that were plated in 10 mL in a petri dish, at a confluence of 2×10^5^ cells/mL, along with treatment with vehicle, FLT3i (midostaurin, 12.5 nM), autophagy inhibitor (chloroquine, 10 μM) or with the combination of FLT3i plus autophagy inhibitor for 48 hours. The cell pellets were then resuspended in 300 μL of 8M urea buffer 0.1M tris HCl with addition of protease and phosphatase inhibitors and subjected to 5 cycles of ultrasound in a bath (UltraSonic Cleaner 750, Unique) at 45 W for 5 minutes, interspersing agitation in vortexing and cooling in an ice bath. Then, the samples were centrifuged at 20000 g for 30 minutes at 4°C. The protein extract (supernatant) was collected and quantified based on Bradford method (Cat. No.: 5000006) (BioRad). Sample preparation represents the fundamental step to allow quantification adequate measurement through the mass spectrometer and therefore consists of several steps, such as: complete solubilization of proteins with the aid of chaotropic agents (urea); complete enzymatic digestion by enzyme (trypsin), decomplexation/fractionation of peptides of interest by solid phase separation (SPE) techniques, based on size, charge and hydrophobicity; removal of contaminants and interferents. After quantification by the Bradford method, aliquots of 100 µg of total extract protein content of the samples were subjected to enzymatic digestion with trypsin. For this, the proteins were reduced with 10 µg/µL DTT for 5 minutes at 98°C and alkylated with iodoacetamide (IAA) (50 µg/µL) for 20 minutes in the dark. Samples were diluted 5 times with 0.1 M Tris solution, pH 8.8 for addition of trypsin in a 1:50 ratio (enzyme:substrate) and kept for 2 hours at 37ºC. Subsequently, a new rate was added of modified trypsin (Cat. No.: V5111) (Promega) in a 1:25 ratio (enzyme:substrate) and the reaction was maintained overnight at 37ºC. Samples were purified in reverse-phase extraction columns (Oasis–Waters), according to the manufacturer’s instructions, dried in speedvac and sent to FioCruz Paraná (Carlos Chagas Institute, Brazil). The proteomic analysis was carried out in collaboration with the FioCruz using liquid chromatography coupled to mass spectrometry (LC-MS/MS) on the Orbitrap Fusion Lumos system. Analysis was performed using maxquant and perseus software. Heatmap was performed using the ClustVis software (<https://biit.cs.ut.ee/clustvis/>). Venn diagram was performed using the Molbiotools software (<https://molbiotools.com/listcompare.php>). Volcano plot was performed using the GraphPad Prism software version 8. GSEA was performed using the Broad Institute software (<https://www.gsea-msigdb.org/gsea/index.jsp>).

| **Supplementary Table S1. List of reagents.** | | |
| --- | --- | --- |
| **Reagent** | **Identifier** | **Company** |
| MOLM13 cell line | ACC 554 | DSMZ (Braunschweig, Germany) |
| MV4-11 cell line | CRL-9591 | ATCC (Manassas, Virginia, USA) |
| APC-Annexin V | 640920 | Biolegend, San Diego, California, USA |
| Midostaurin (PKC412) | HY-10230 | MedChemExpress (Princeton, New Jersey, USA) |
| Quizartinib (AC220) | HY-13001 |  |
| ROC-325 | HY-103706 |  |
| Propidium iodide | P1304MP | Molecular Probes, Eugene, Oregon, USA |
| shRNA nonspecific control | sc-108080 | Santa Cruz Biotechnology (Dallas, Texas, USA) |
| shRNA targeting the ATG5 (Autophagy Related 5 gene) | sc-41445-V |  |
| shRNA targeting the ATG7 (Autophagy Related 7 gene) | sc-41447-V |  |
| Bafilomycin A1 | B1793 | Sigma-Aldrich (St. Louis, Missouri, USA) |
| Chloroquine | C6628 |  |
| Dimethyl Sulfoxide | D2650 |  |
| Puromycin | P8833 |  |
| Methylthiazol tetrazolium | M5655 |  |
| Acridine orange free-permeabilization cell probe | A6014 |  |
| Histopaque® | 10771 |  |
| RPMI 1640 culture medium | 11875119 | Thermo Fisher Scientific (Waltham, Massachusetts, USA) |
| Penicillin and Streptomycin 10 000 U/mL | 15140122 |  |
| Fetal Bovine Serum | 12657029 |  |
| PBS buffer | 70011044 |  |
| SuperSignal™ West Dura Extended Duration Substrate System | 34076 |  |

| **Supplementary Table S2. List of antibodies used to perform western blotting.** | | | | |
| --- | --- | --- | --- | --- |
| **Company** | **Target** | **Identifier** | **Species** | **Dilution** |
| Cell Signaling | p-STAT5^Tyr694^  STAT5  p-AKT^Ser473^  AKT  p-mTOR^Ser2448^  mTOR  p-P70S6K^Thr421/Ser424^  P70S6K  p-ULK1^S757^  ULK1  p62  LC3BI/II  ATG5  ATG7  Cas3/CleavedCas3 | 9359S  94205S  4060S  4685S  2971S  2972S  9204S  9202S  14202S  8054S  88588S  2775S  12994S  2631S  9662S | Rabbit IgG  Rabbit IgG  Rabbit IgG  Rabbit IgG  Rabbit IgG  Rabbit IgG  Rabbit IgG  Rabbit IgG  Rabbit IgG  Rabbit IgG  Mouse IgG1  Rabbit IgG  Rabbit IgG  Rabbit IgG  Rabbit IgG | 1:5000  1:5000  1:1000  1:1000  1:1000  1:1000  1:2000  1:2000  1:2000  1:2000  1:1000  1:1000  1:2000  1:2000  1:2000 |
| Novus Biologicals | β-Actin | NB600-501 | Mouse IgG1 | 1:5000 |

| **Supplementary Table S3. List of equipments.** | |
| --- | --- |
| **Reagent/Equipment** | **Company** |
| PowerPlex® 16 HS system | Promega, Madison, Wisconsin, USA) |
| ABI 3500 Sequence Detector System | Life Technologies (Carlsbad, California, USA) |
| FACSCanto | BD Bioscience, San Diego, California, USA |
| Leica Microscope Fluorescence DMI6000B | Leica, Wetzlar, Germany |
| Gel Doc XR+ system | Bio-Rad, Hercules, California, USA |
| Orbitrap Fusion Lumos System | Thermo Fisher Scientific, Waltham, Massachusetts, USA |
| IVIS Lumina Imaging System® | Caliper LifeSciences, Hopkinton, Massachusetts, USA |

| **Supplementary Table S4. List of protein acronyms.** |
| --- |
| STAT5: Signal Transducer And Activator Of Transcription 5 |
| mTOR: Mechanistic Target Of Rapamycin Kinase |
| AKT: AKT Serine/Threonine Kinase |
| P70S6K: Ribosomal protein S6 kinase |
| ULK1: Unc-51 Like Autophagy Activating Kinase 1 |
| LC3B I/II: Microtubule Associated Protein 1 Light Chain 3 Beta, LC3 Protein |
| SQSTM1/p62: Sequestosome 1 |
| BAX: BCL2 Associated X, Apoptosis Regulator |
| SMARCA4: SWI/SNF Related BAF Chromatin Remodeling Complex Subunit ATPase 4 |
| SUMO1: Small Ubiquitin Like Modifier 1 |
| RFC4: Replication Factor C Subunit 4 |
| GATD3/C21orf33: Glutamine Amidotransferase Class 1 Domain Containing 3 |
| RBBP4: RB Binding Protein 4, Chromatin Remodeling Factor |
| EIF4G: Eukaryotic Translation Initiation Factor 4 Gamma |
| WDR3: WD Repeat Domain 3 |
| AQR: Aquarius Intron-Binding Spliceosomal Factor |
| RFC2: Replication Factor C Subunit 2 |
| ARPC1B: Actin Related Protein 2/3 Complex Subunit 1B |
| GNB2L1/RACK1: Receptor For Activated C Kinase 1 |
| SRSF6: Serine And Arginine Rich Splicing Factor 6 |
| TDP43: TAR DNA-binding protein 43 |

| **Supplementary Table S5. Blood count of the *FLT3*-ITD AML animal model pre- and post-treatment.** | | | | | | |
| --- | --- | --- | --- | --- | --- | --- |
|  | **Pre-Treatment**  **(Day 7 post-transplant)** | | | **Post-Treatment**  **(Day 14 post-transplant)** | | |
| **Mice** | **WBC**  **(x10^3^/μL)** | **Hb**  **(g/dL)** | **Platelets (x10^3^/μL)** | **WBC**  **(x10^3^/μL)** | **Hb (g/dL)** | **Platelets (x10^3^/μL)** |
| Vehicle | 1.5  0.4  0.9  0.8  1.4 | 13.2  10.8  13.8  12.4  18.2 | 508  268  291  289  135 | X  1.1  1.9  1.5  X | X  9  10.8  12  X | X  381  359  275  X |
| Midostaurin | 1.4  0.8  1.2  1.3  0.9 | 12.5  8.3  12.3  11.5  12.4 | 250  97  335  296  129 | 1.4  X  2  0.9  X | 9.8  X  11.3  10.1  X | 340  X  307  210  X |
| Midostaurin and Chloroquine | 1.5  1.3  0.8  0.1  0.7 | 11.3  10.4  10.8  5.8  12.4 | 185  354  264  158  327 | 2.4  3.4  1.6  1.3  2.4 | 11.2  8.4  11  10.8  10.3 | 186  165  160  188  203 |
| Chloroquine | 1.2  0.3  0.7  0.4  1 | 7.2  10.1  10.6  11.5  14.7 | 413  193  126  244  123 | X  1.1  X  2.2  X | X  6.6  X  11  X | X  291  X  208  X |

Abbreviations: Hb: hemoglobin; WBC: white blood cell counts.

| 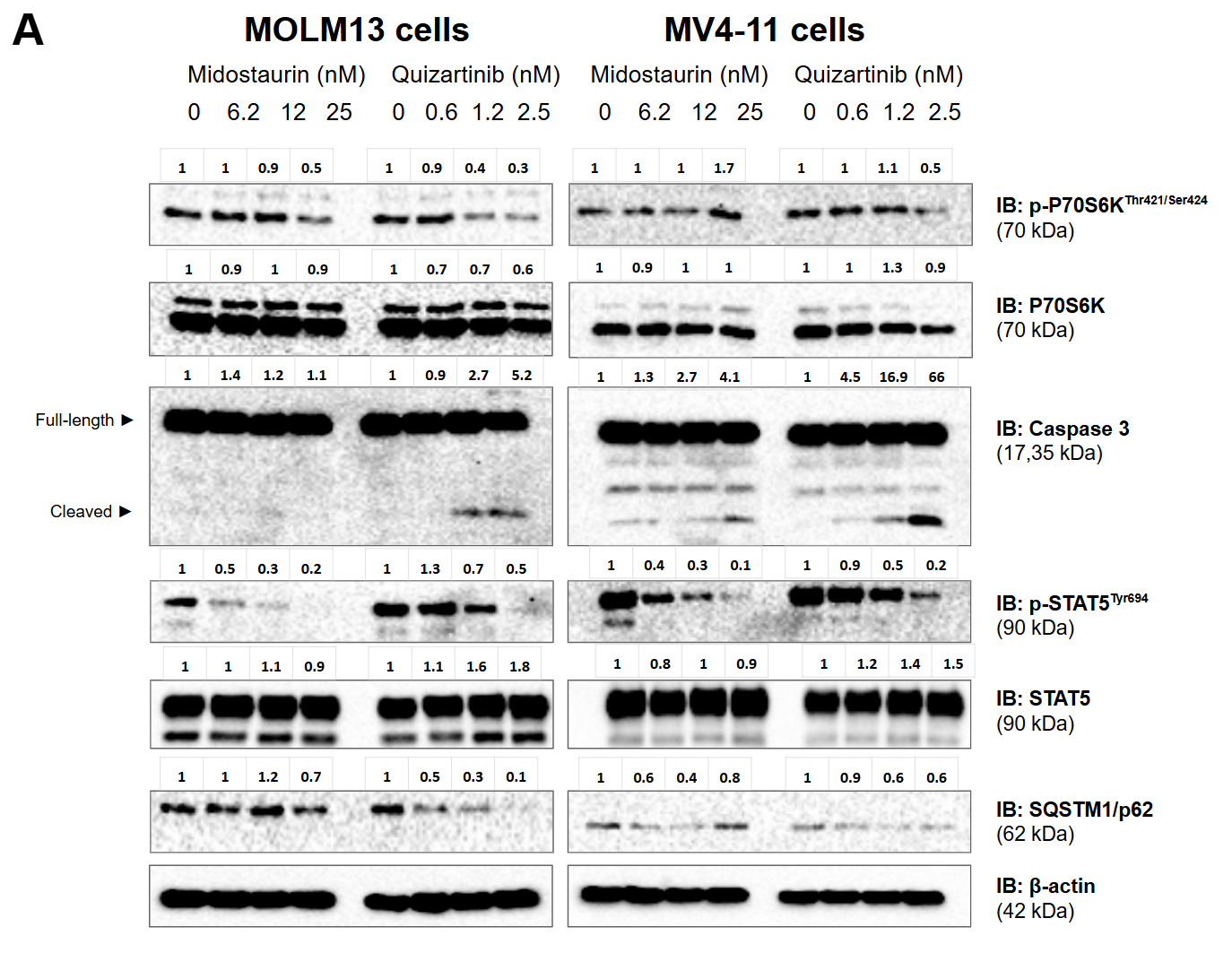 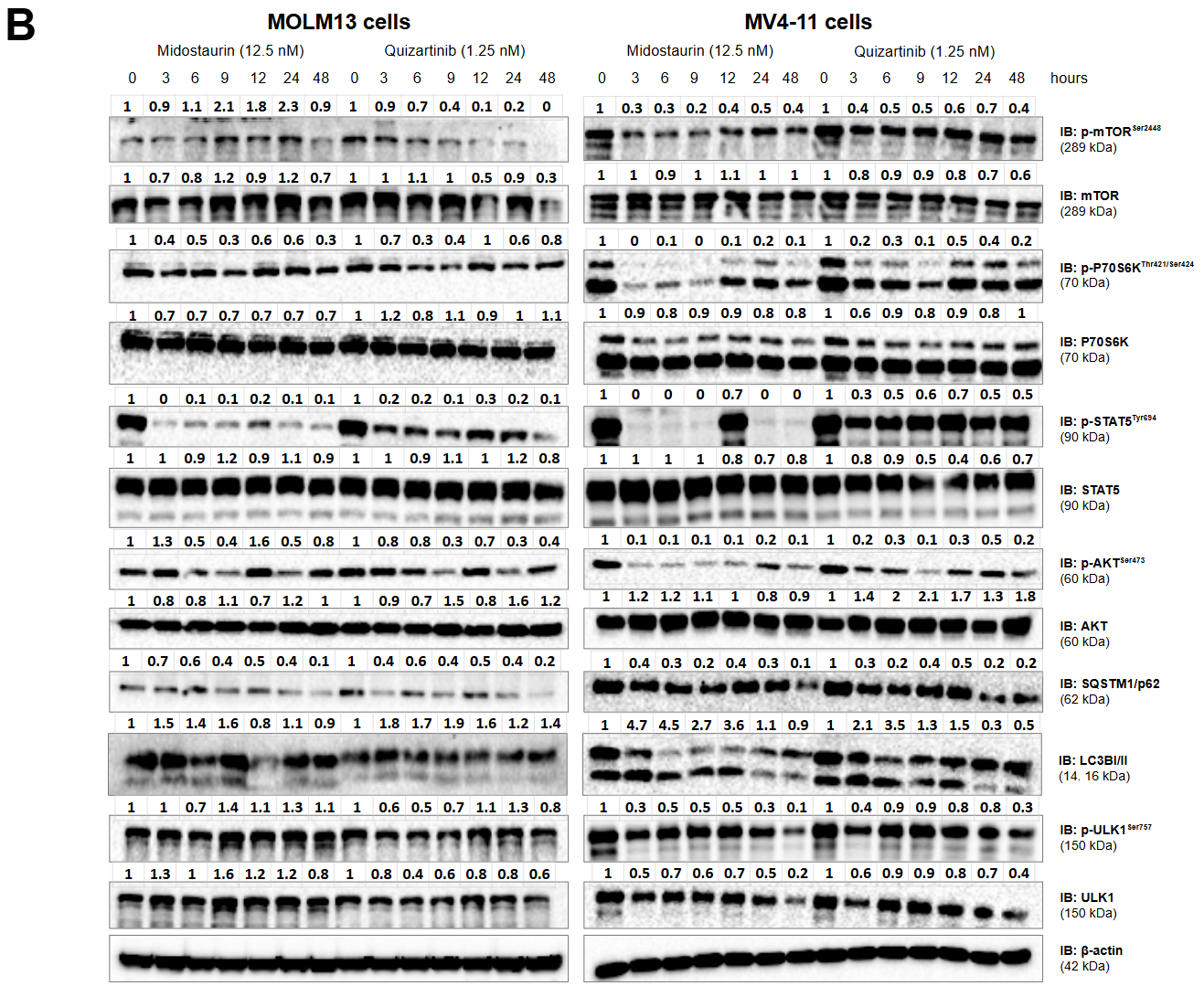 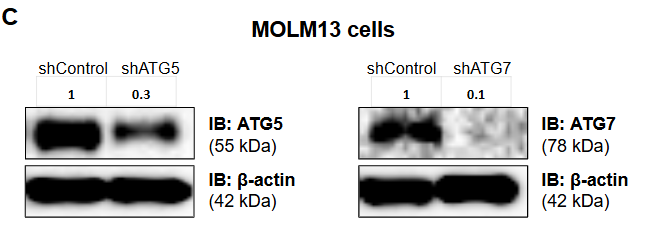 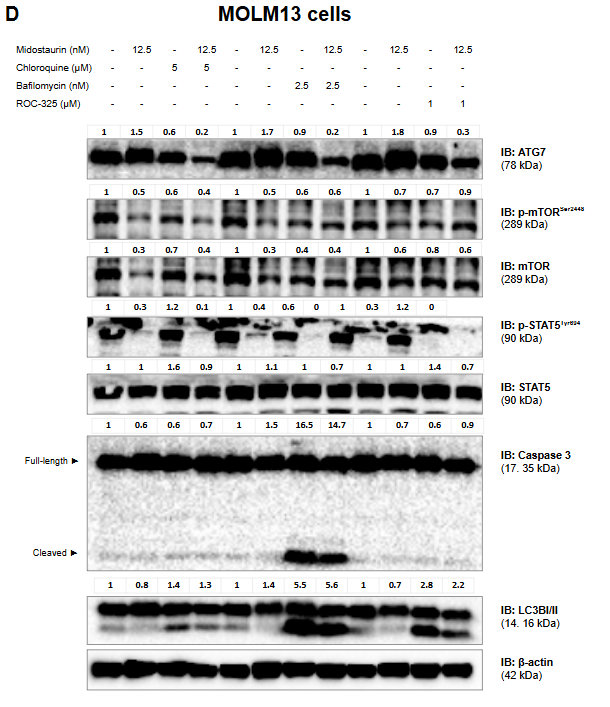 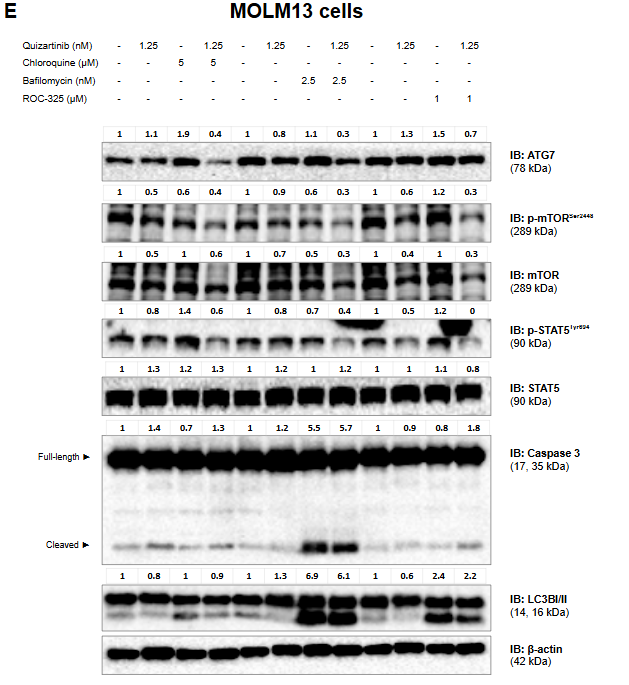 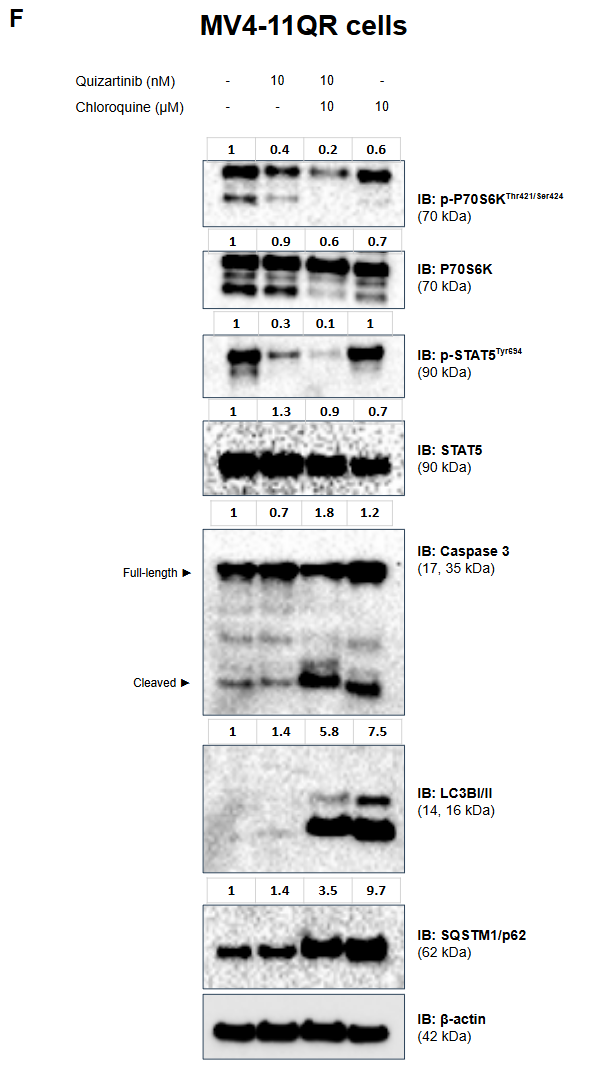 |
| --- |
| **Supplementary Figure S1. Quantitative analysis of protein expression assessed by western blot.** (A) Densitometric analysis of the Western blot shown in Figure 1D, depicting the dose-dependent effects of midostaurin and quizartinib on protein expression in MOLM-13 and MV4-11 cells. (B) Densitometric analysis of the Western blot shown in Figure 1E, depicting the time-dependent effects of midostaurin and quizartinib on protein expression in MOLM-13 and MV4-11 cells. (C) Densitometric analysis of the Western blot shown in Figure 4A, depicting the protein expression of ATG5 and ATG7 in MOLM-13 cells after gene silencing. (D) Densitometric analysis of the Western blot shown in Figure 5A, depicting the effects of midostaurin, alone or in combination with chloroquine, bafilomycin A1, or ROC-325, in the MOLM-13 cell line. (E) Densitometric analysis of the Western blot shown in Figure 5A, depicting the effects of quizartinib, alone or in combination with chloroquine, bafilomycin A1, or ROC-325, in the MOLM-13 cell line. (F) Densitometric analysis of the Western blot shown in Figure 6E, depicting the effects of quizartinib, alone or in combination with chloroquine, on protein expression in MV4-11QR cells. Values associated with proteins bands were normalized to standard β-actin for the relative expression measure. The variation in the band intensity was calculated relative to the corresponding vehicle. Cleaved Cas3 and LC3BII protein levels were normalized to total Cas3 and LC3BI protein levels, respectively, enabling the determination of the conversion rate. |

| **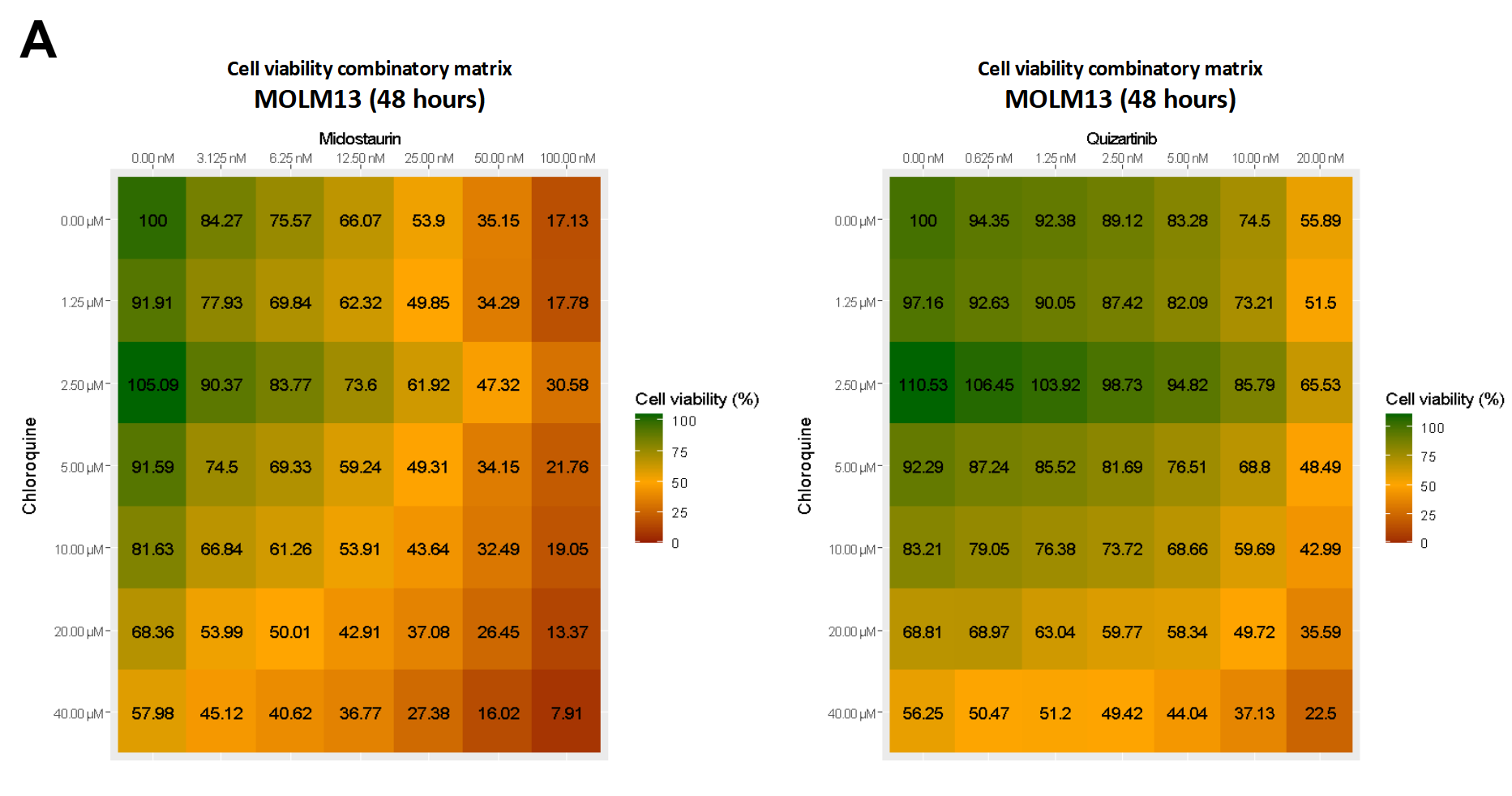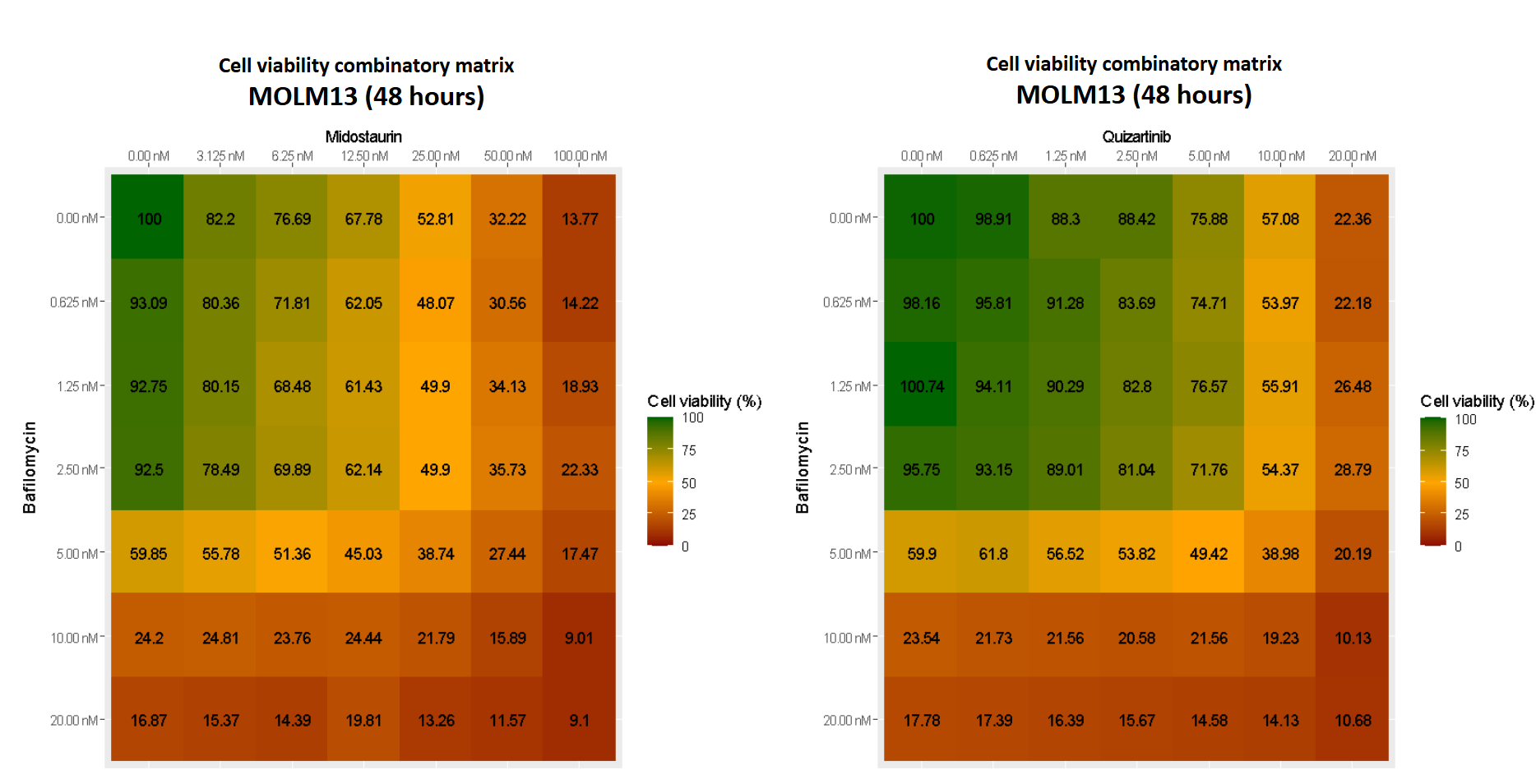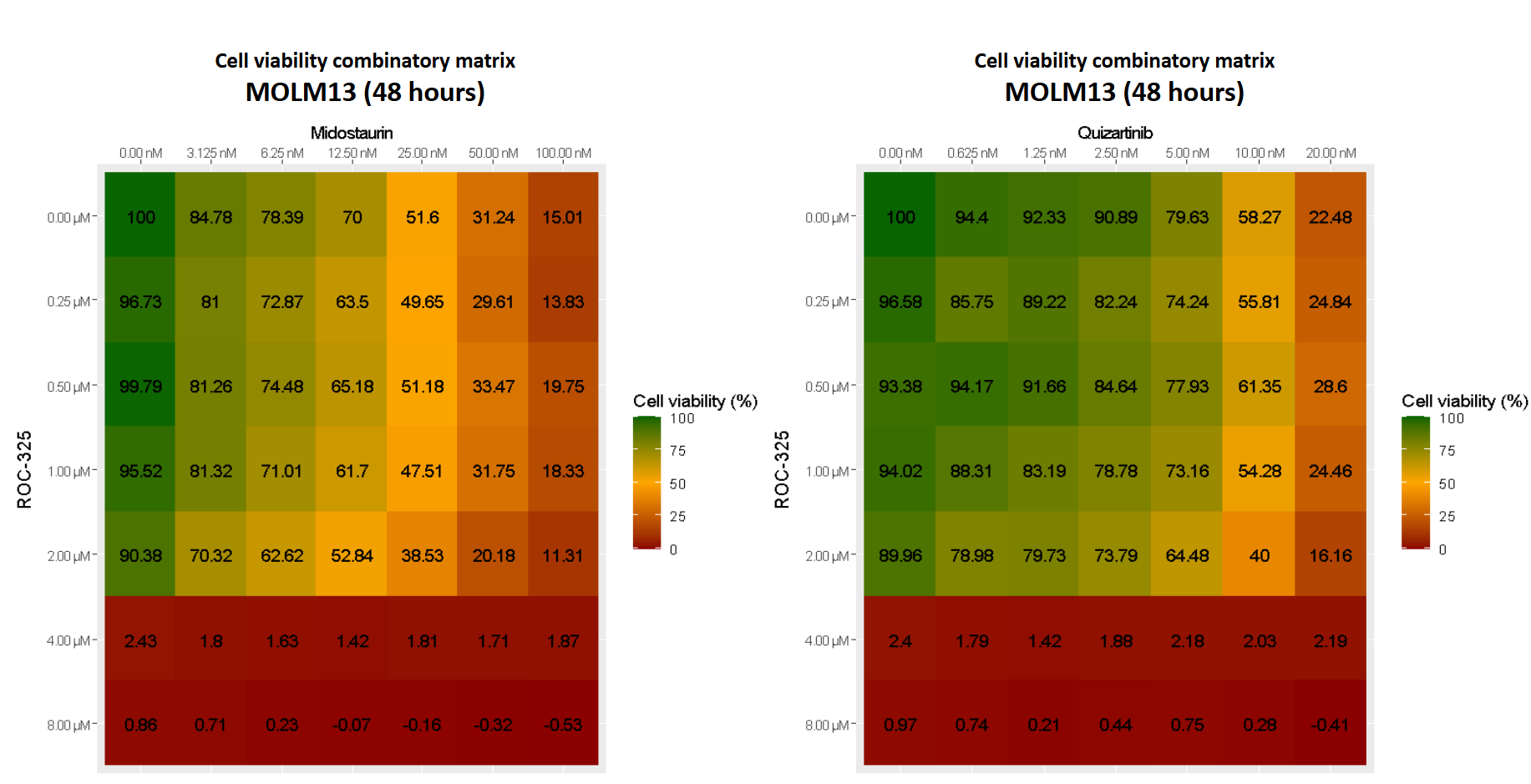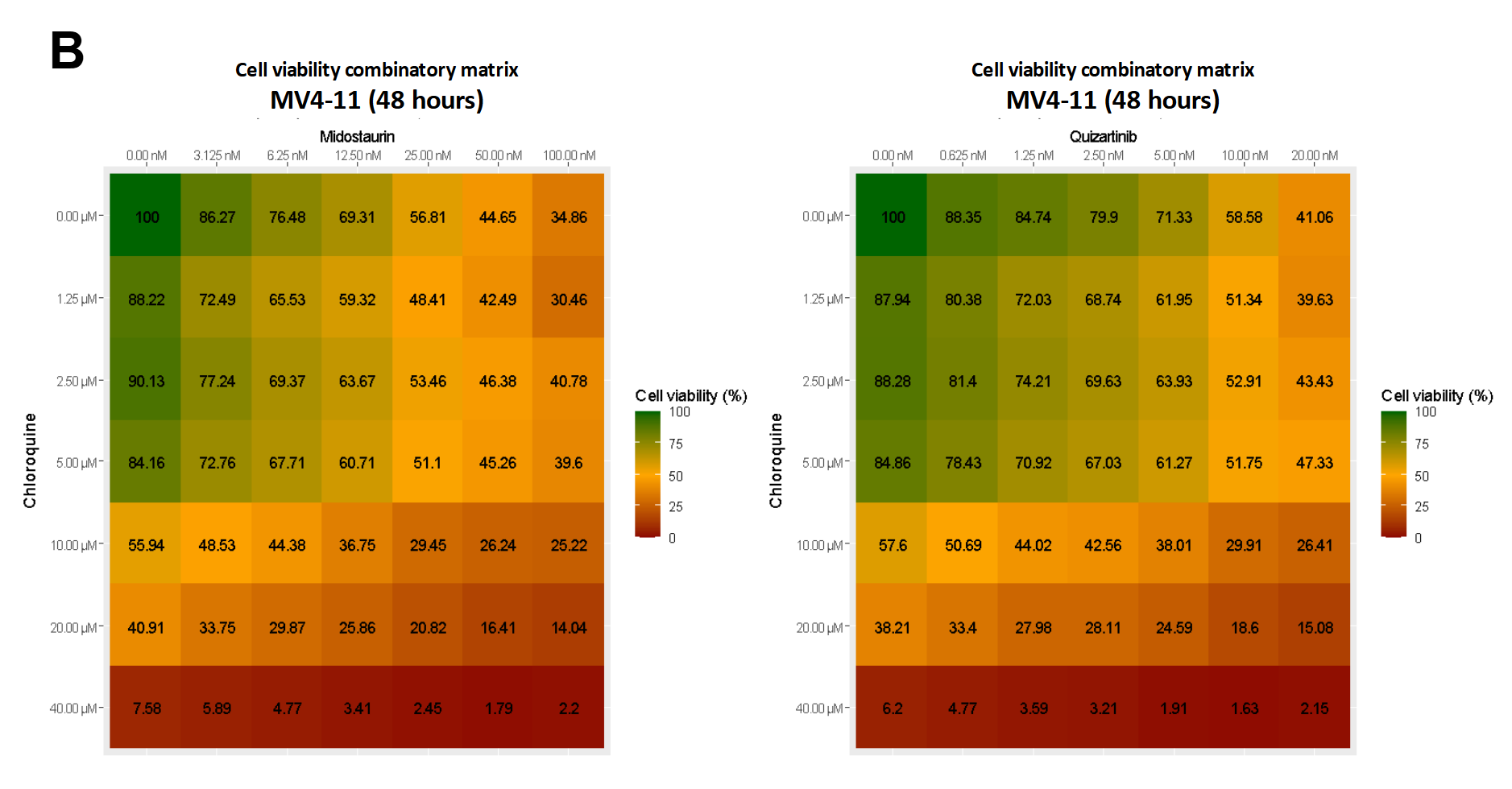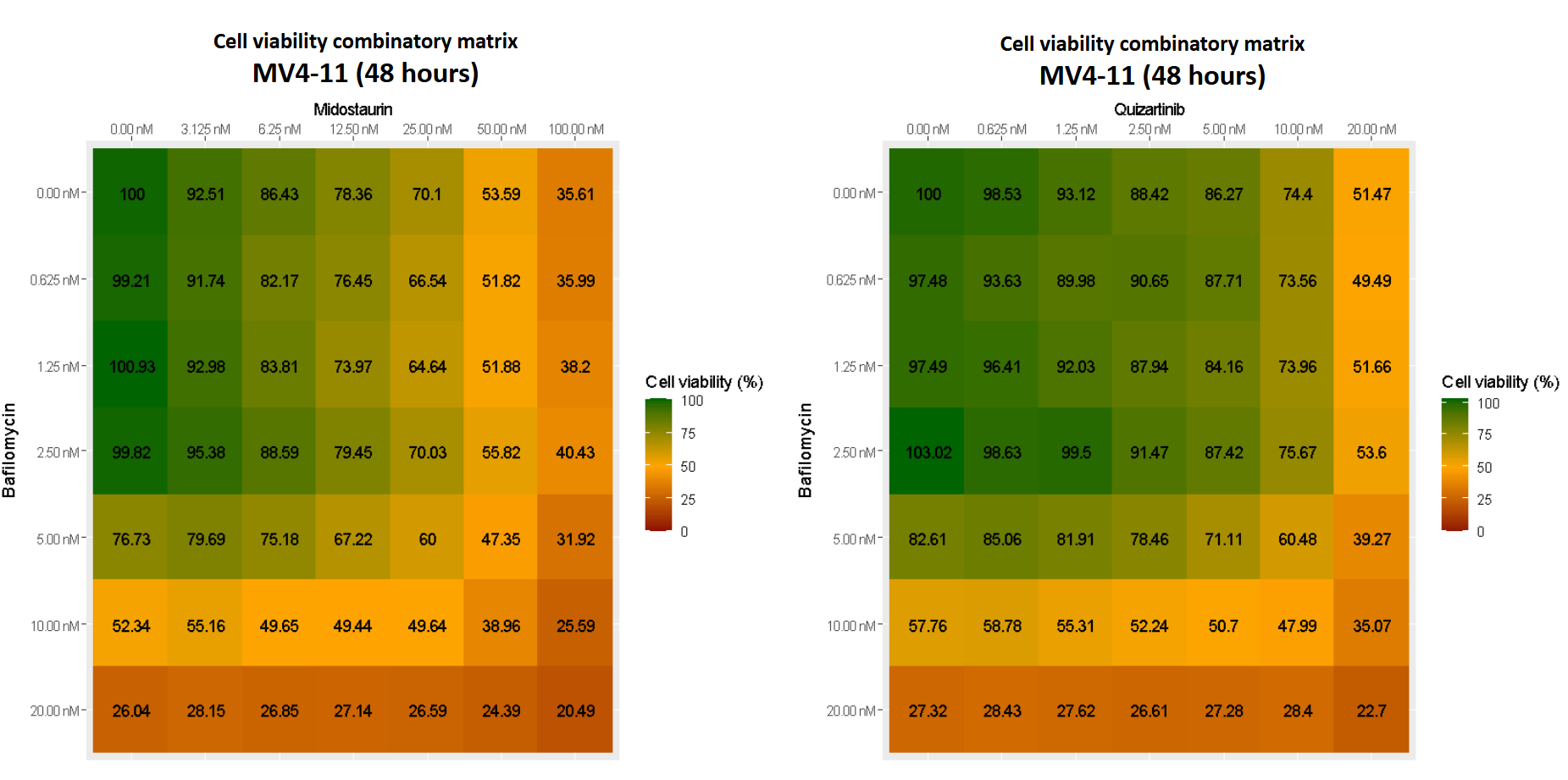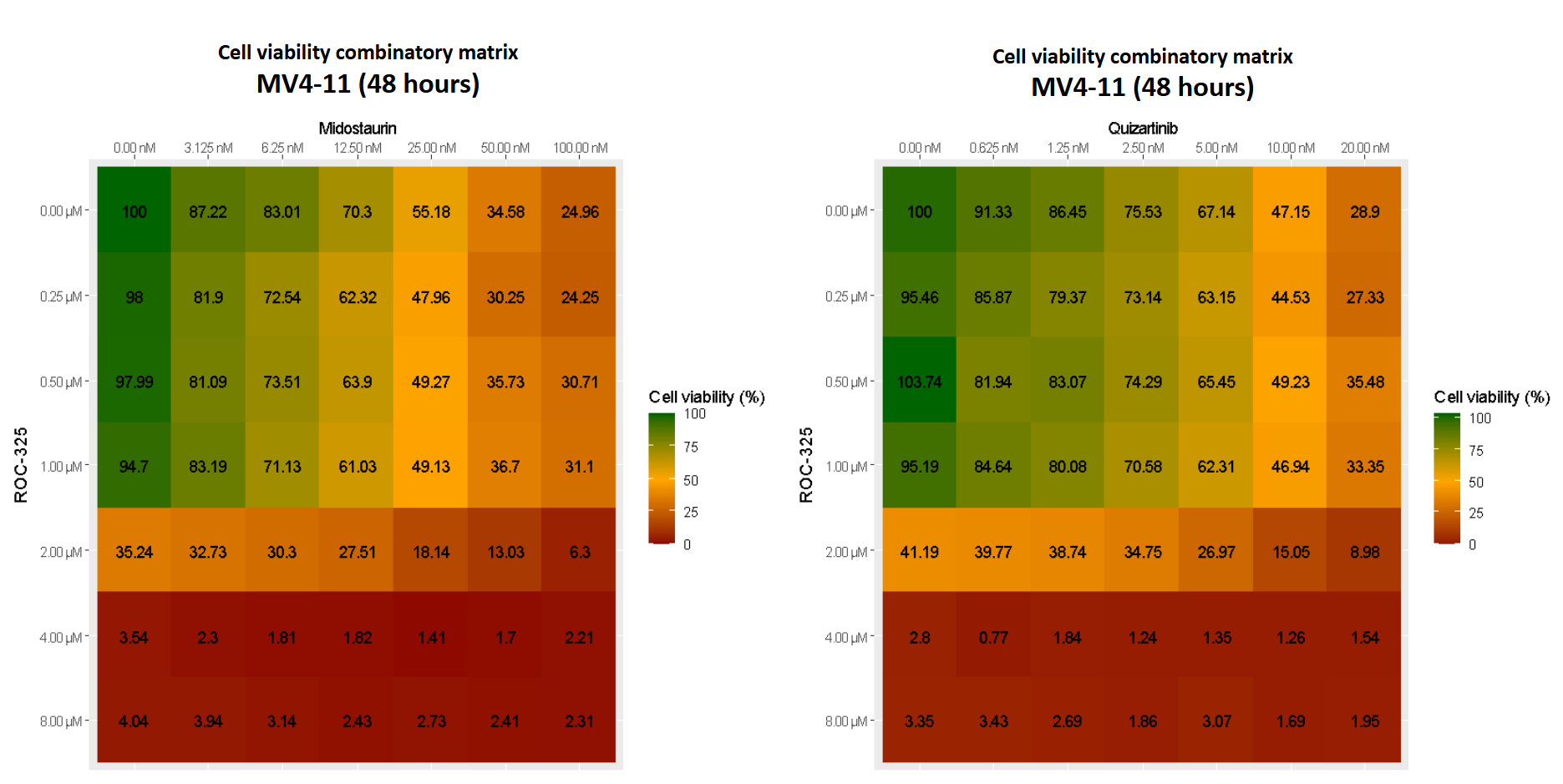** |
| --- |
| **Supplementary Figure S2. The combination of FL3i and autophagy inhibitors reduces cell viability in *FLT3*-ITD models.** Cell viability after treatment with vehicle, midostaurin (3.125, 6.25, 12.5, 25, 50, and 100 nM) or quizartinib (0.625, 1.25, 2.5, 5, 10, and 20 nM) alone or combined with vehicle, chloroquine (1.25, 2.5, 5, 10, 20, and 40 μM), bafilomycin A1 (0.625, 1.25, 2.5, 5, 10, and 20 nM) or ROC-325 (0.25, 0.5, 1, 2, 4, and 8 μM) in MOLM13 cells (A) and MV4-11 cells (B). Dose-response cytotoxicity of the combined treatment was performed by the methylthiazoletetrazolium (MTT) assay after 48 hours. Values expressed represent the percentage of viable cells for each condition relative to the control. |

| 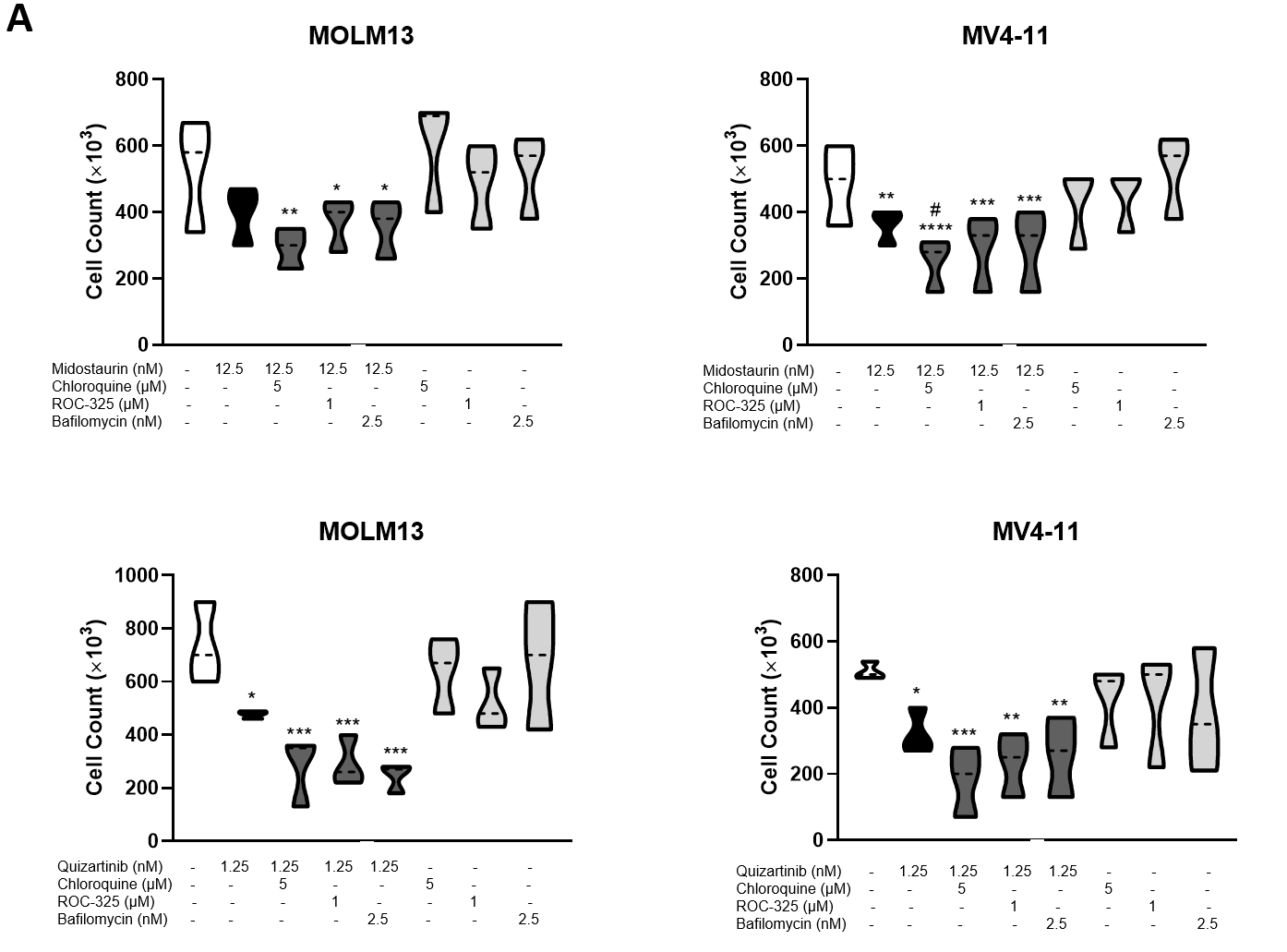 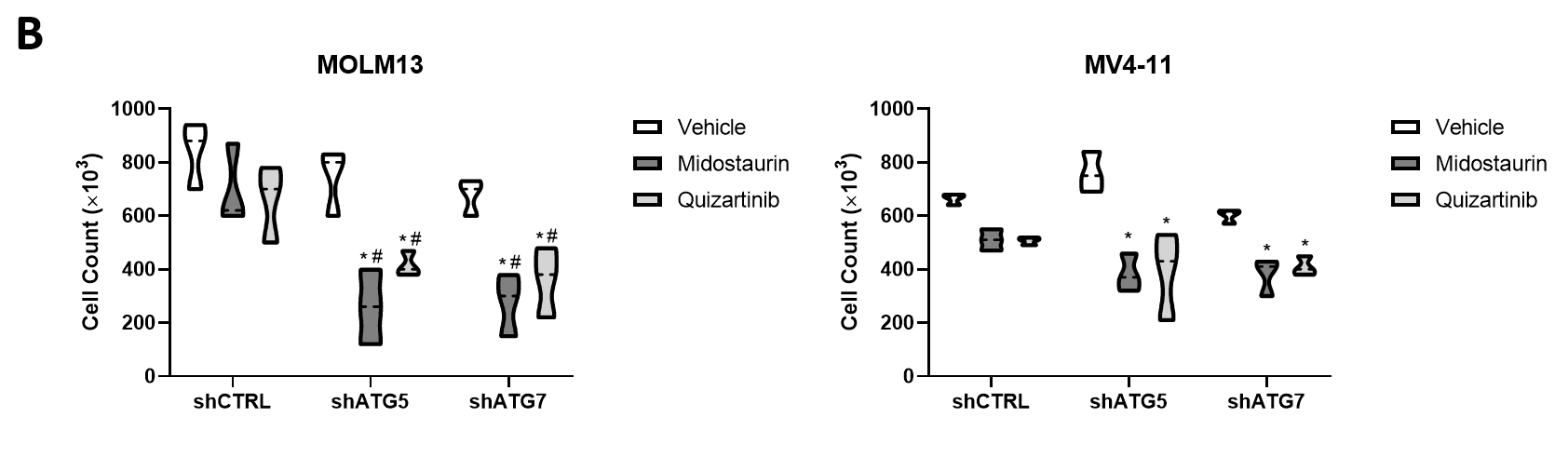 |
| --- |
| **Supplementary Figure S3. The combination of FLT3i and pharmacological or genetic autophagy inhibition reduces cell count in *FLT3*-ITD AML cell lines.** (A) *In vitro* treatment with midostaurin (12.5 nM) or quizartinib (1.25 nM) combined or not with chloroquine (5 µM), bafilomycin (2.5 nM) or ROC-325 (1 µM) for 48 hours in MOLM13 and MV4-11 cells. (B) *In vitro* treatment with midostaurin (12.5 nM) or quizartinib (1.25 nM) after genetic inhibition of autophagy in MOLM13 and MV4-11 cells (shCONTROL, shATG5 and shATG7) for 48 hours. Violin plots represent the cell counting of at least three independent experiments; *p* values are indicated on the graphs. **p*<0.05; ***p*<0.01; ****p*<0.001; *****p*<0.0001 compared with vehicle; #*p*<0.05 compared to FLT3i alone at the corresponding dose; ANOVA test and Bonferroni post-test. |

| **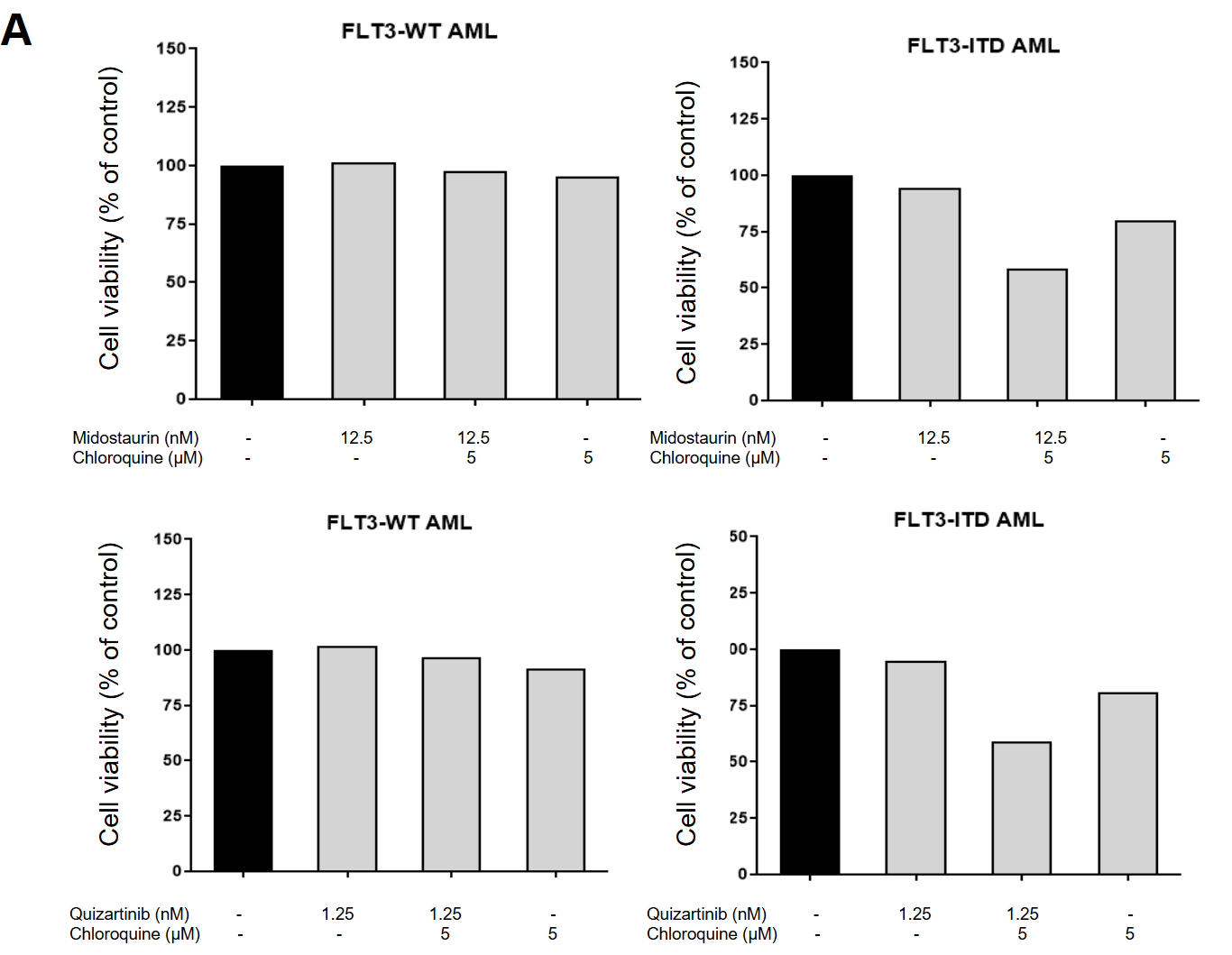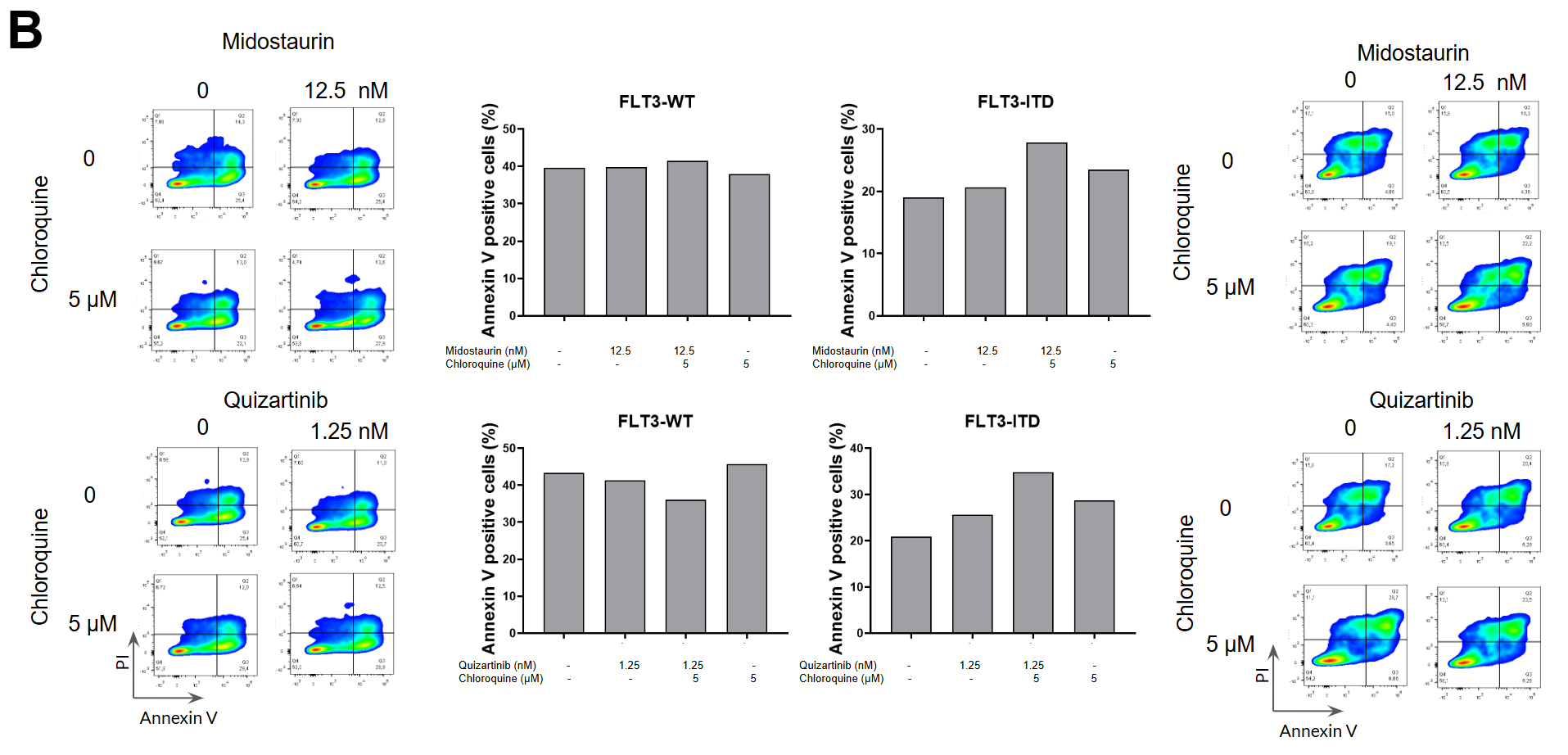** |
| --- |
| **Supplementary Figure S4. The combination of FL3i and chloroquine reduces cell viability and increases apoptosis in primary samples of *FLT3*-ITD AML patients.** (A) *Ex vivo* treatment with midostaurin or quizartinib combined with chloroquine reduces cell viability determined by MTT assay in primary cells from AML patients with *FLT3*-ITD mutation. Primary cells from patients with and without *FLT3*-ITD mutation received 48-hour treatment with vehicle, midostaurin (12.5 nM) or quizartinib (1.25 nM) alone or combined with vehicle or chloroquine (5 μM). The values expressed represent the percentage of viable cells for each condition in relation to the control. (B) *Ex vivo* treatment with midostaurin or quizartinib combined with chloroquine increases cell apoptosis in primary cells from AML patients with *FLT3*-ITD mutation. Apoptosis was detected by flow cytometry in cells treated with vehicle, midostaurin (12.5 nM) or quizartinib (1.25 nM) alone or combined with vehicle or chloroquine (5 μM) for 48 hours; the staining method used was annexin V/PI. Representative density plots are shown for each condition; the upper and lower right quadrants (Q2 and Q3) cumulatively contain the apoptotic population (annexin V+ cells). #*FLT3*-WT AML patient: Woman, 46 years old, Leukocytes: 12.7×103/μL, Hemoglobin: 8.5 g/dL, Platelets: 25×103/μL, % Blasts in BM 94%, absence of the FLT3-ITD variant, wild-type NMP1; #*FLT3*-ITD AML patient: Woman, 32 years old, Leukocytes 8.3×103/μL, Hemoglobin 7.2 g/dL, Platelets 44×103/μL, % Blasts in BM 59%, presence of the FLT3-ITD variant, wild-type NMP1. |

| **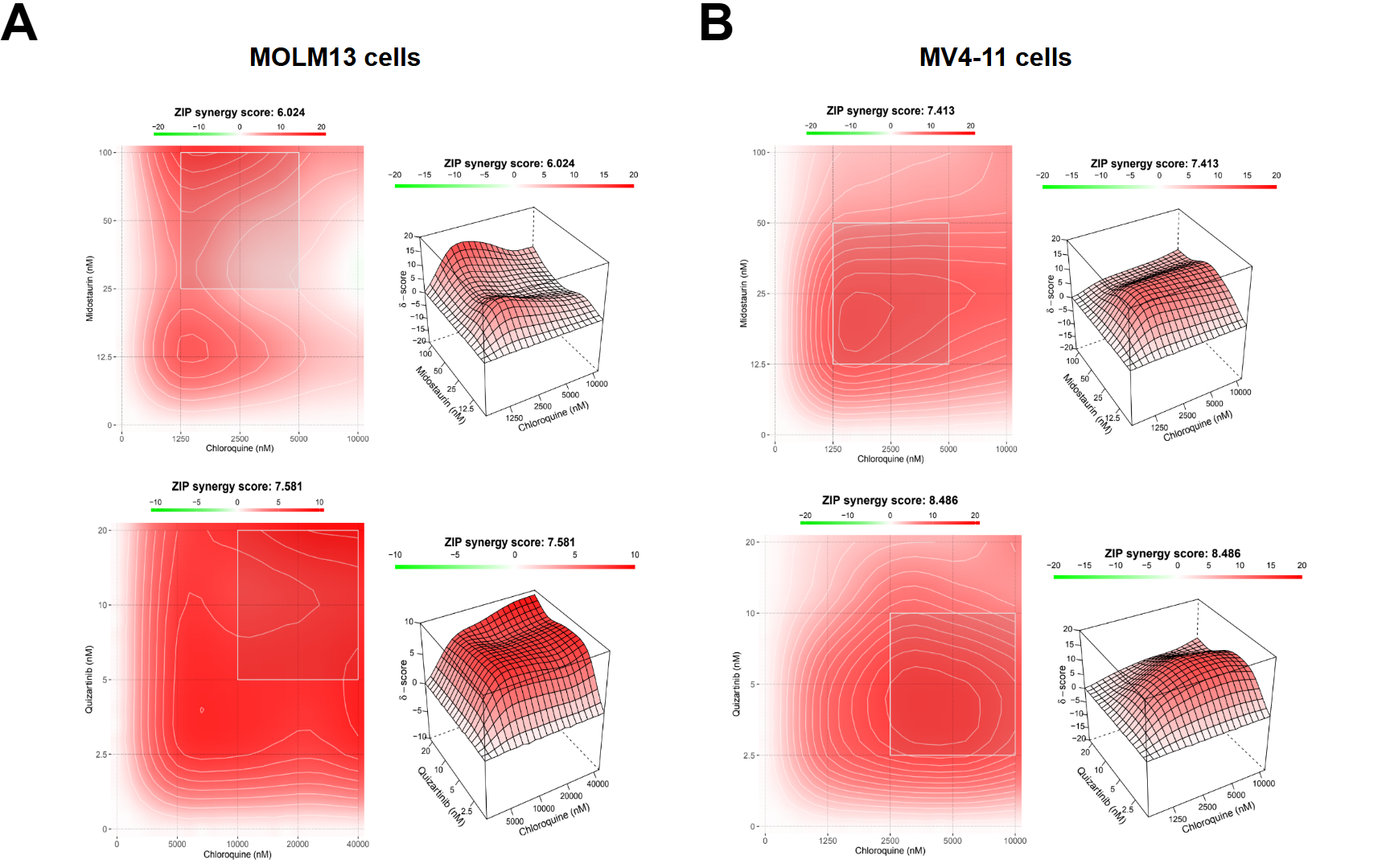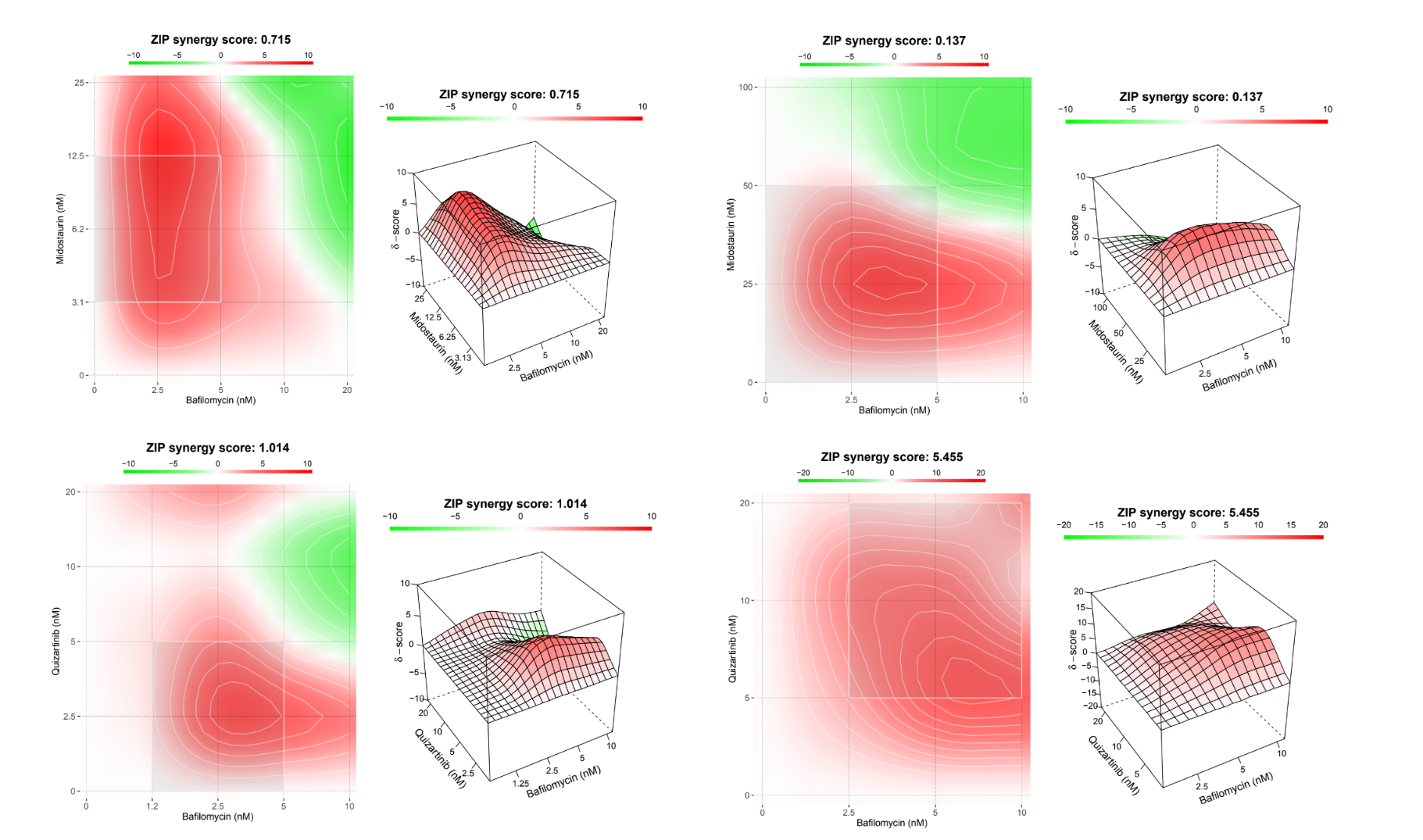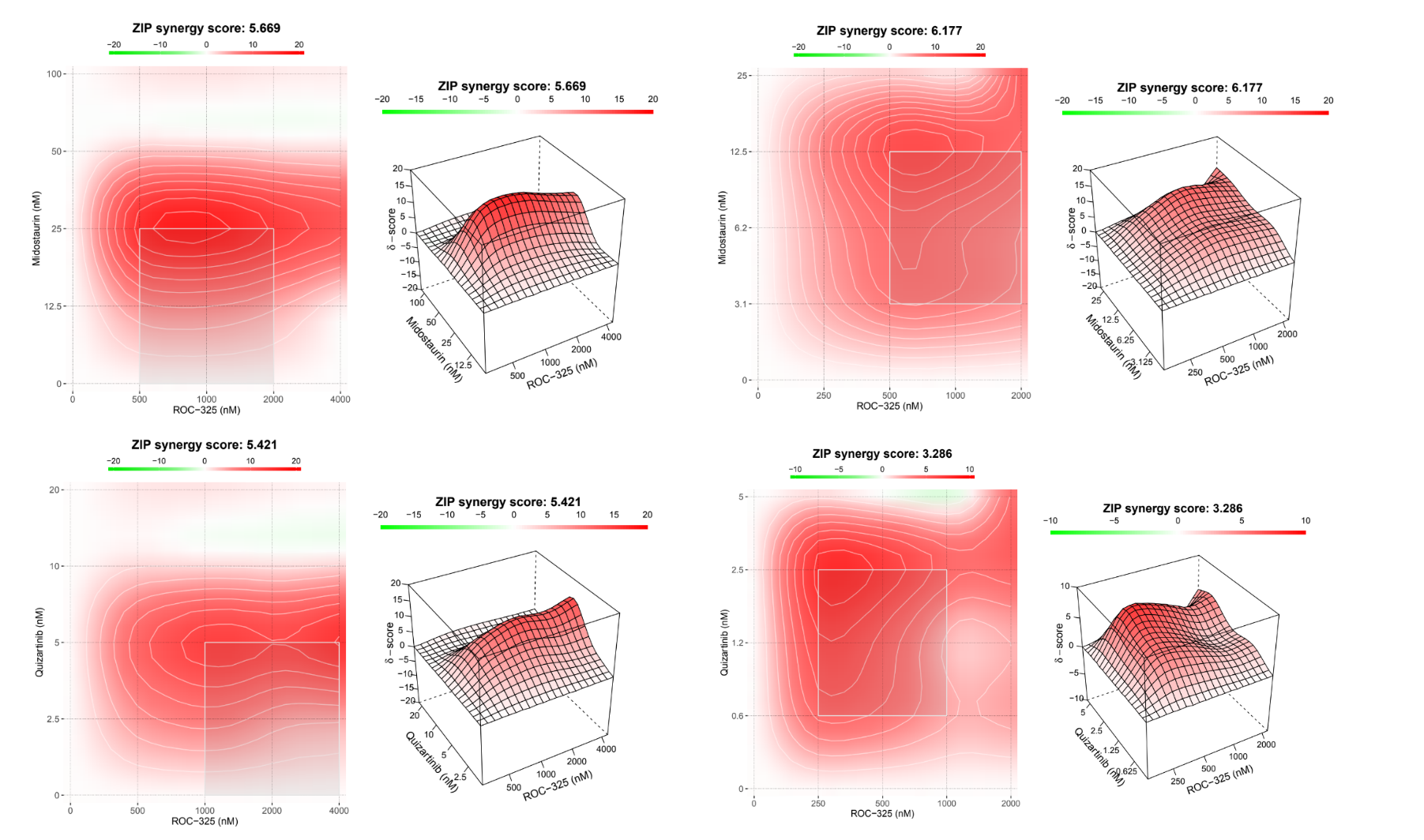** |
| --- |
| **Supplementary Figure S5. The combination of FLT3i and autophagy inhibitors demonstrate an additive effect in *FLT3*-ITD models.** Determination of the main level of synergy between midostaurin and quizartinib with chloroquine, bafilomycin A1 and ROC-325 in MOLM13 cells (A) and MV4-11 cells (B). Values are expressed as the percentage of viable cells for each condition relative to untreated controls. The ZIP score was determined using the software <https://synergyfinder.fimm.fi/>. |

| **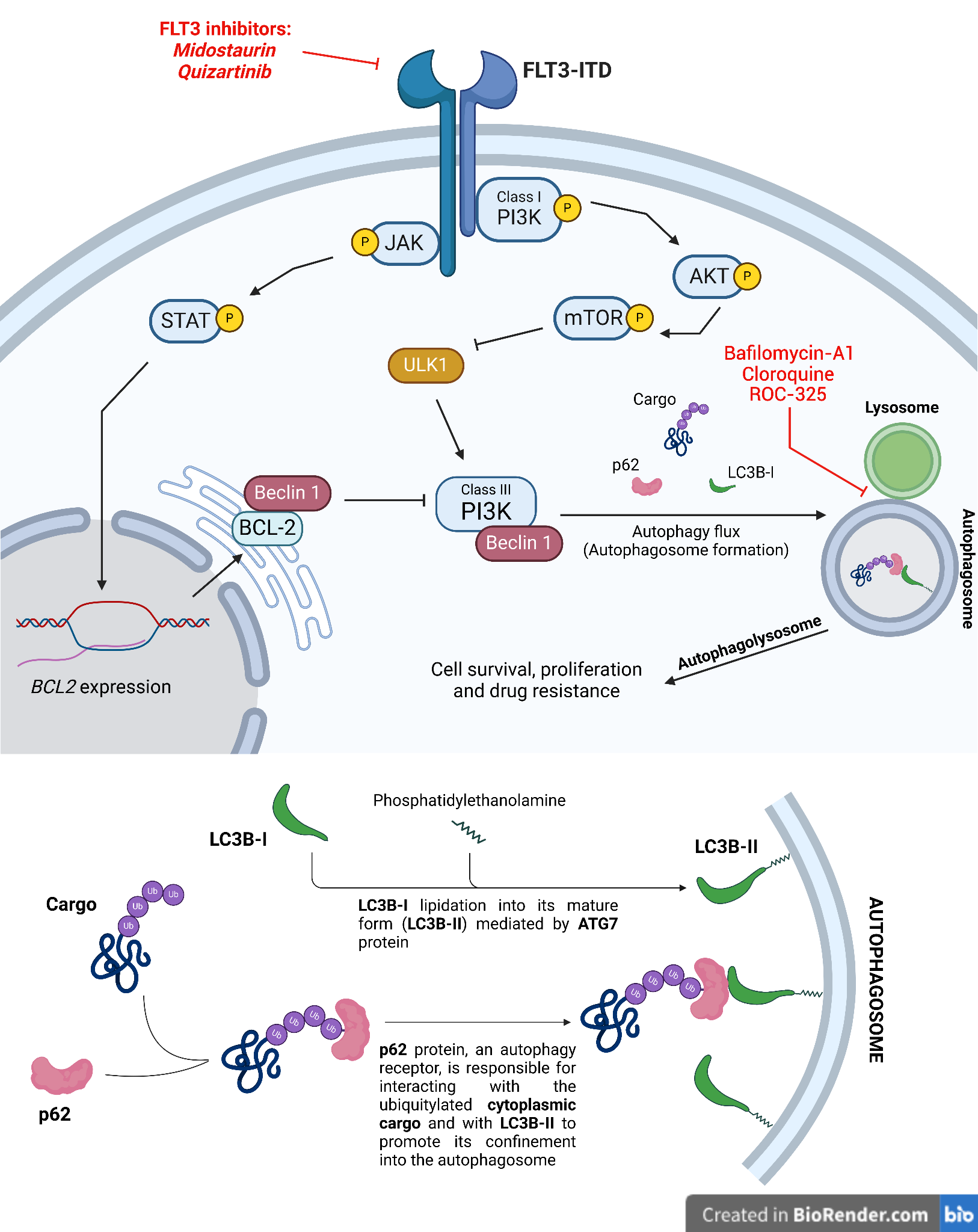** |
| --- |
| **Supplementary Figure S6. Autophagy as a limiting mechanism of the efficacy of FLT3 inhibitors.** (A) The *FLT3*-ITD mutation results in the constitutive activation of FLT3-regulated signaling cascades, such as JAK/STAT and PI3K/AKT/mTOR, which, respectively, increases the gene expression of BCL-2, which in turn controls the autophagy process when complexed with Beclin 1 and mediates the blockade of the ULK1 protein, which is essential for the initiation of the autophagosome. Treatment with FLT3 inhibitors, by inhibiting these pathways, favors the autophagy process, which in turn functions as an anti-apoptotic mechanism resulting in cell survival. Pharmacological inhibition of autophagy, however, appears to be a potential inducer of apoptosis, increasing the efficiency of tyrosine kinase inhibitor drugs. (B) The p62 and LC3B-I/II molecules act in the formation of the autophagosome and are consumed at the end of the autophagy process. The p62 protein, or SQSTM1, conducts the polyubiquitinated structures to the primitive autophagosome, also called phagophore, where it will oligomerize to the LC3B-II protein, after lipidation of the precursor LC3B-I mediated by ATG7. Image created by the author with the BioRender.com software. |
