# Supplementary material for: Autophagy inhibition potentiates the antileukemic effect of FLT3 inhibitors and overcomes resistance in FLT3-ITD acute myeloid leukemia
Source: Cell Death Discov. 2026 Mar 24;12:174. doi: 10.1038/s41420-026-03037-7 (PMC13039817; doi:10.1038/s41420-026-03037-7)

**SUPPLEMENTARY MATERIALS**

**FULL AND UNCROPPED WESTERN BLOTS**

**Full and uncropped Western Blots corresponding to DOSE COURSE EXPERIMENTS (48 hours) ilustrated in the Figure 1D of the manuscript.**

MOLM13 cells

**Ladder – Vehicle - Midostaurin 6.25 nM - 12.5 nM - 25 nM - Ladder – Vehicle - Quizartinib 0.625 nM, 1.25 nM, 2.5 nM**

p-P706SK


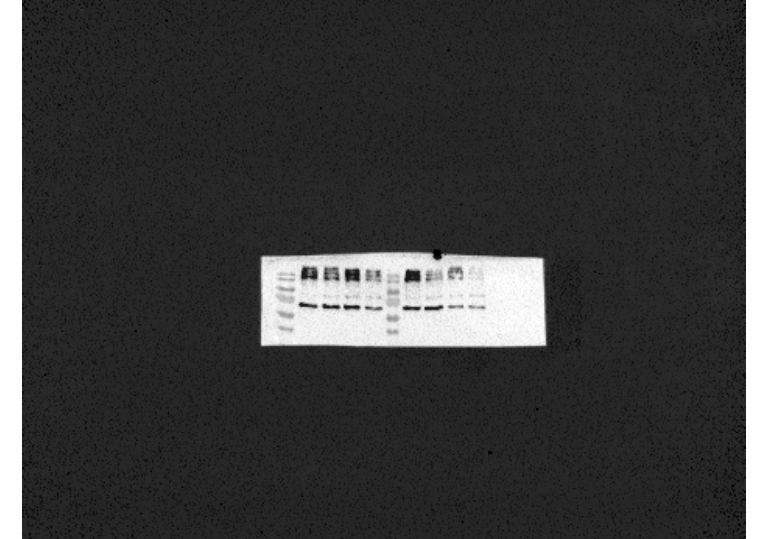


P70S6K


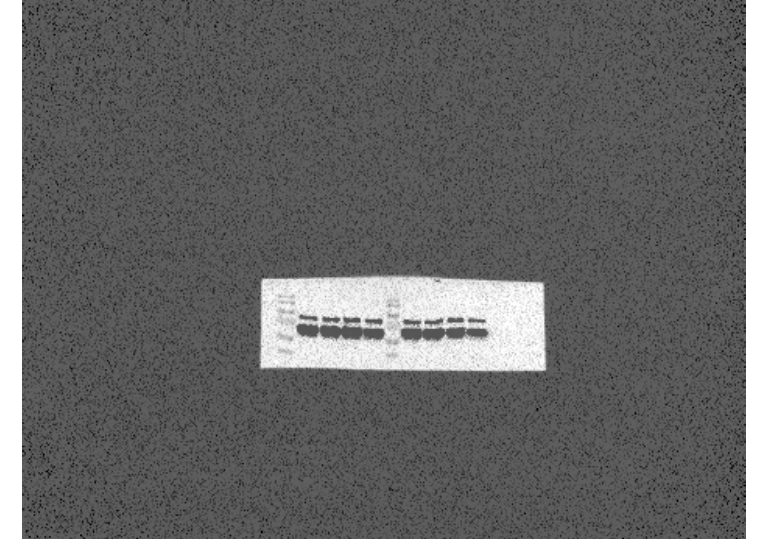


Caspase 3


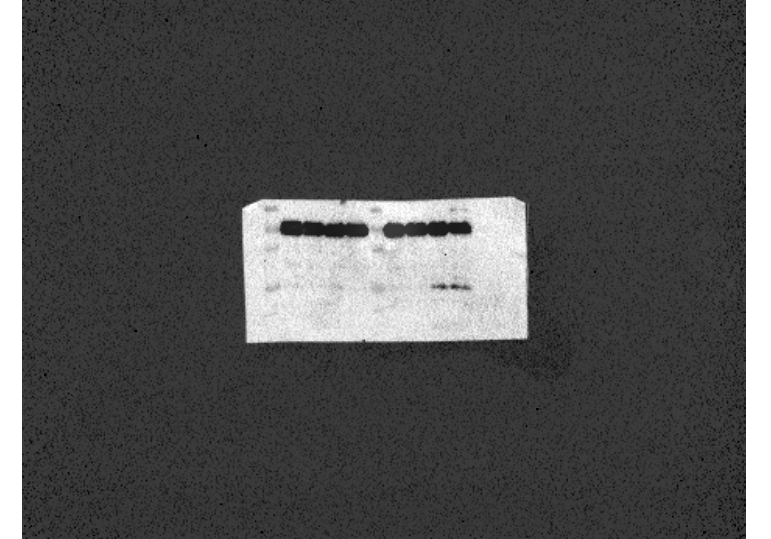


p-STAT5


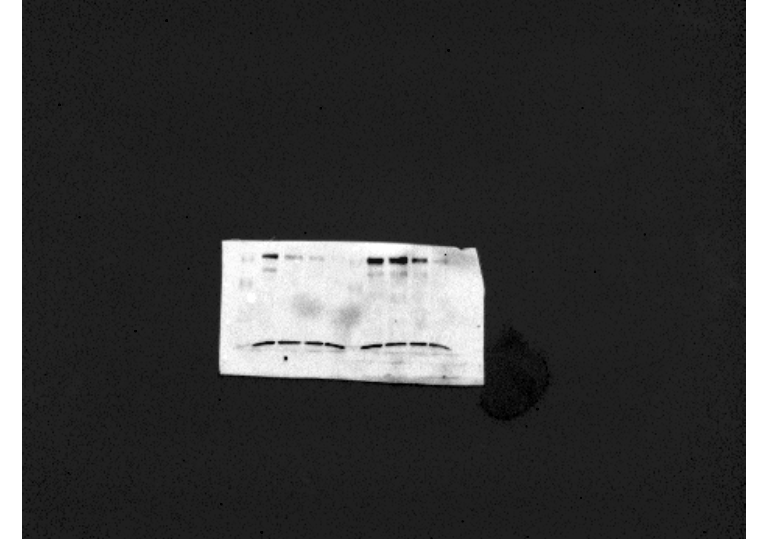


STAT5


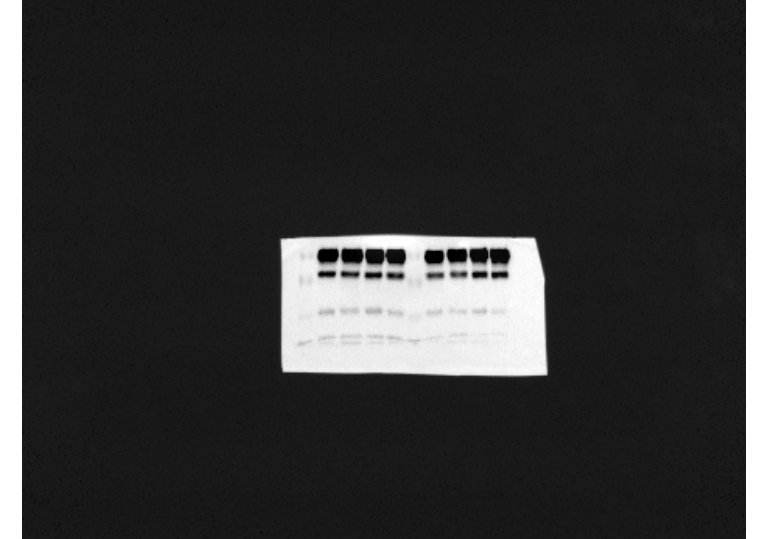


p62


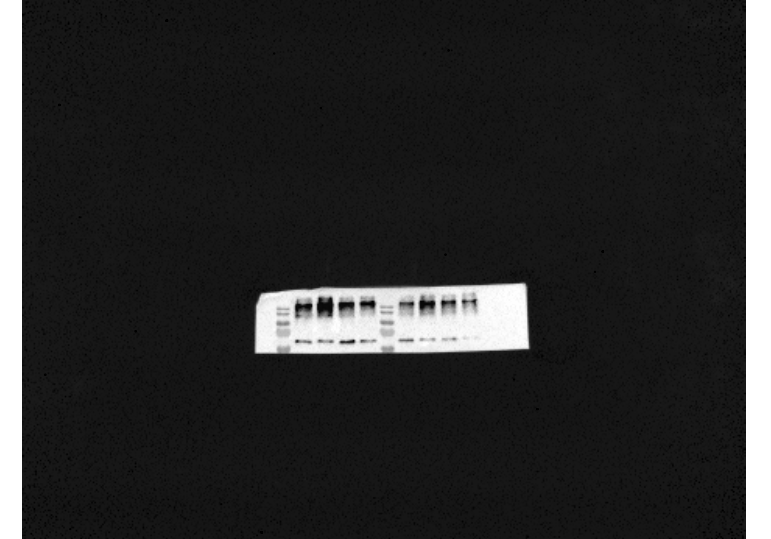


β-Actin from p-P70S6K and P70S6K gel


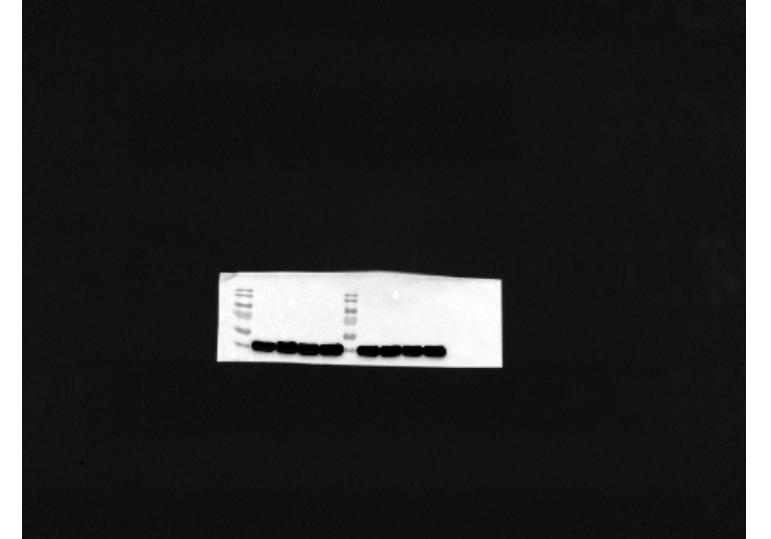


β -Actin from Caspase3 and p62 gel


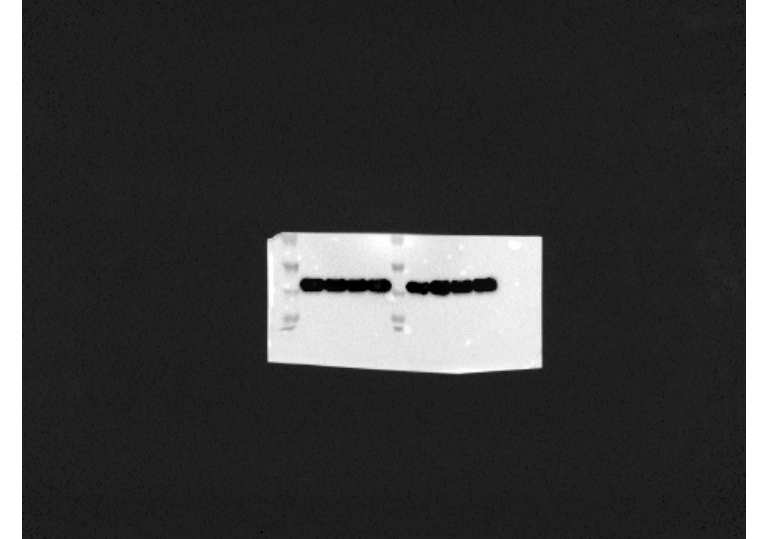


β -Actin from p-STAT5 and STAT5 gel


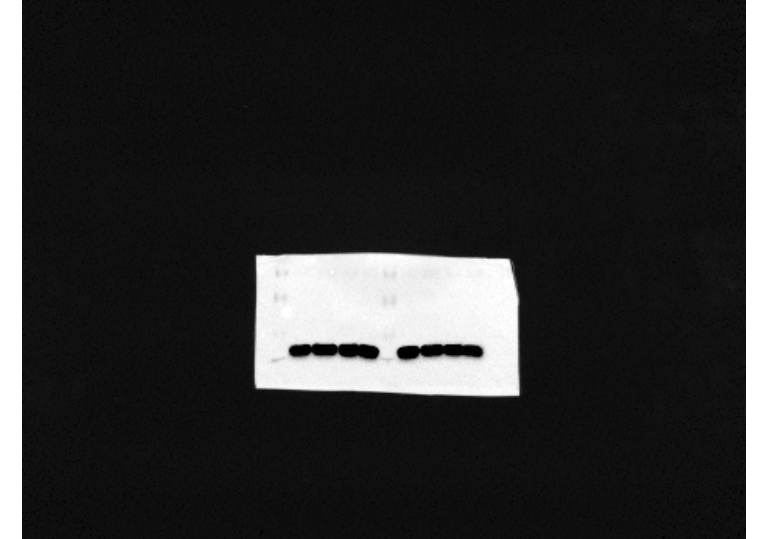


MV4-11 cells

**Ladder – Vehicle - Midostaurin 6.25 nM - 12.5 nM - 25 nM - Ladder – Vehicle - Quizartinib 0.625 nM, 1.25 nM, 2.5 nM**

p-P70S6K


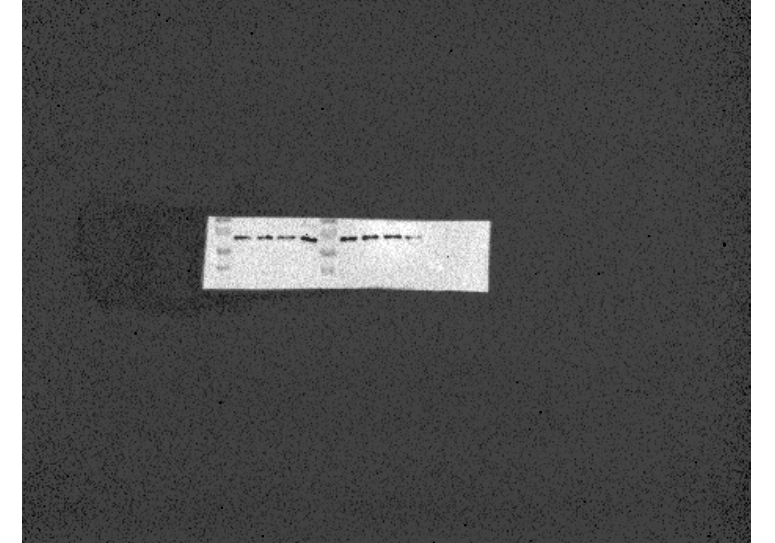


P70S6K


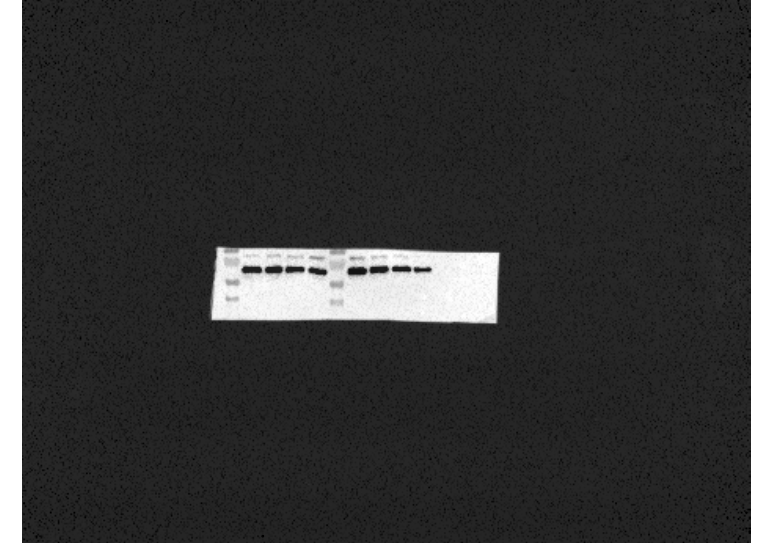


Caspase 3


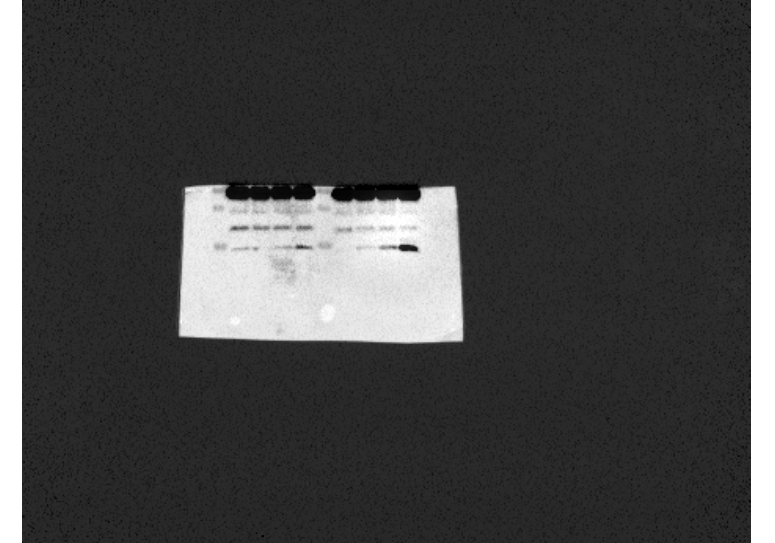


p-STAT5


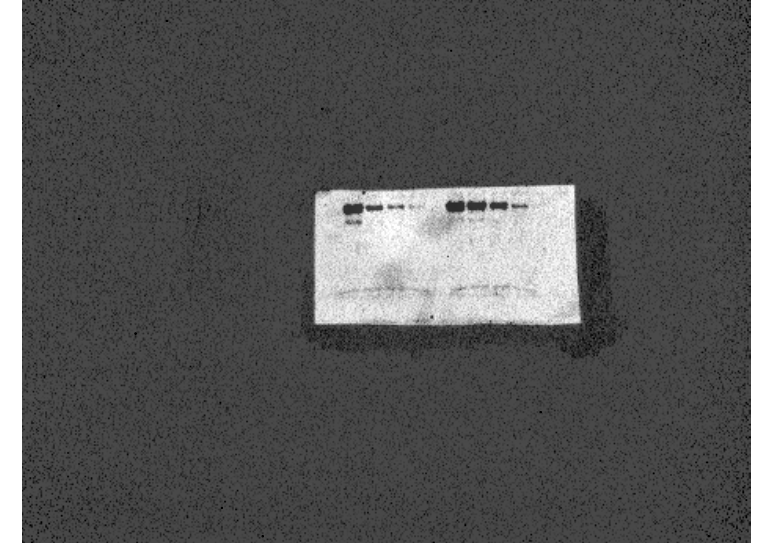


STAT5


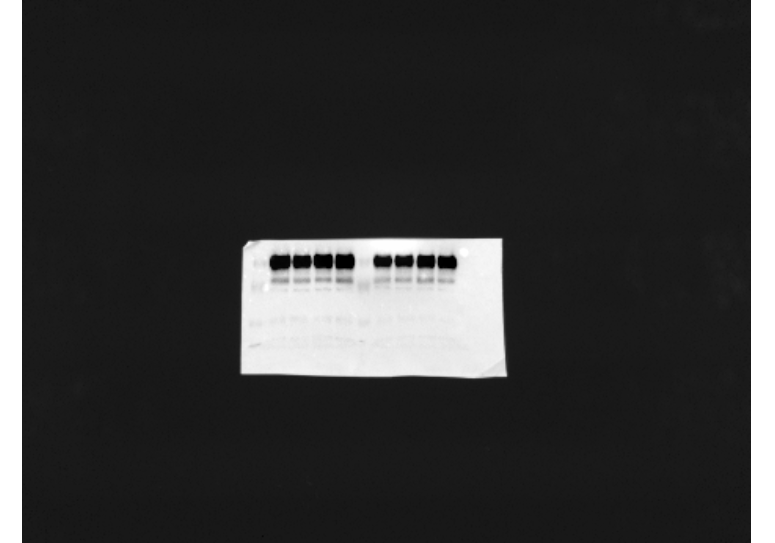


p62


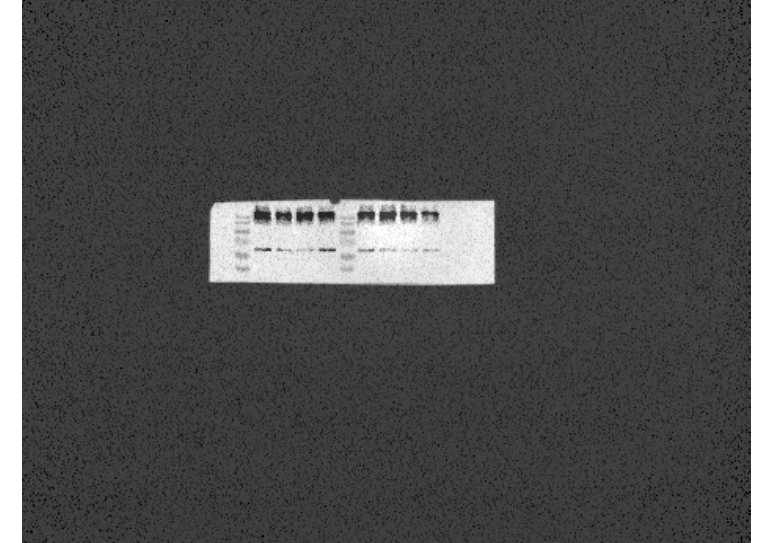


β -Actin from p-P70S6K and P70S6K gel


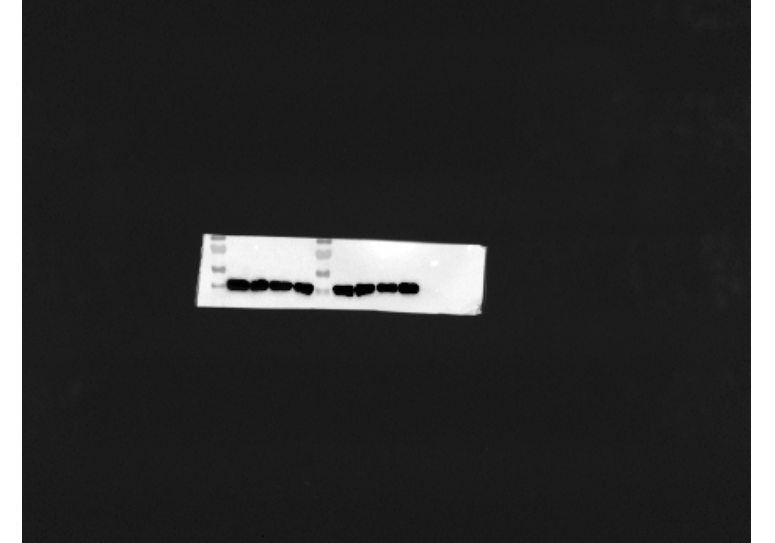


β -Actin from Caspase3 and p62 gel


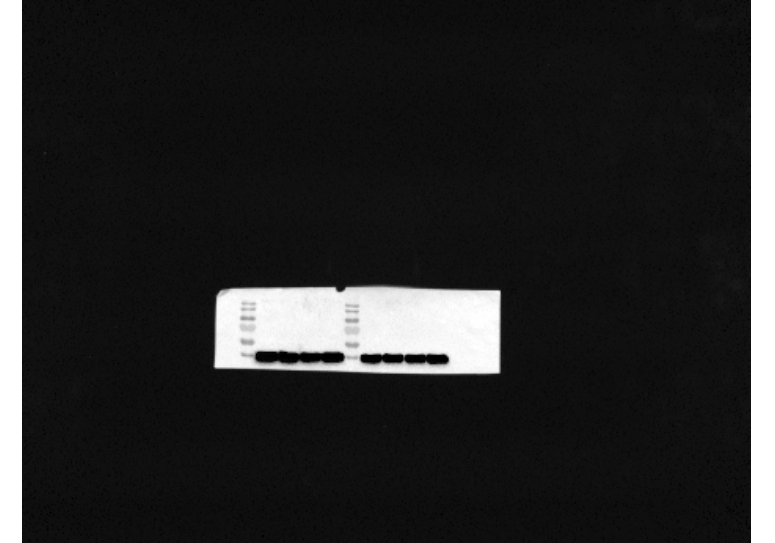


β -Actin from p-STAT5 and STAT5 gel


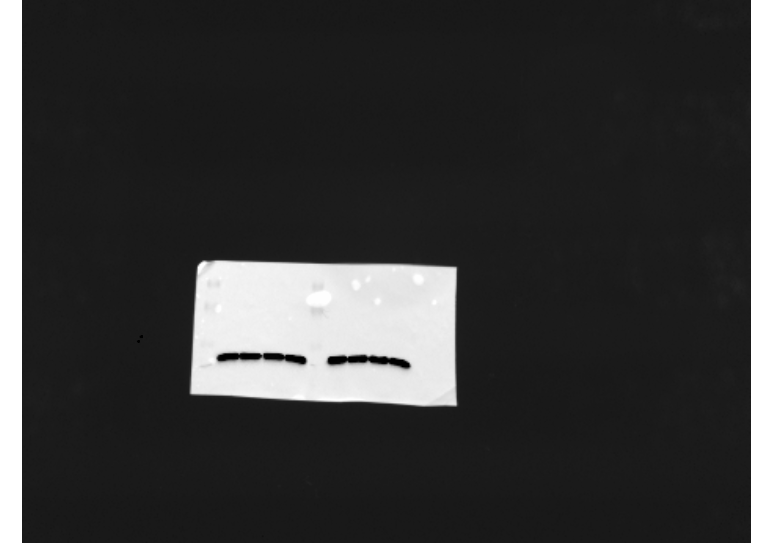


**Full and uncropped Western Blots corresponding to TIME COURSE EXPERIMENTS (Midostaurin 12.5 nM and Quizartinib 1.25 nM) ilustrated in the Figure 1E of the manuscript.**

MOLM13 cells

**Ladder – Midostaurin 0h – 3h – 6h – 9h – 12h – 24h – 48h – Quizartinib 0h – 3h – 6h – 9h – 12h – 24h – 48h**

p-mTOR


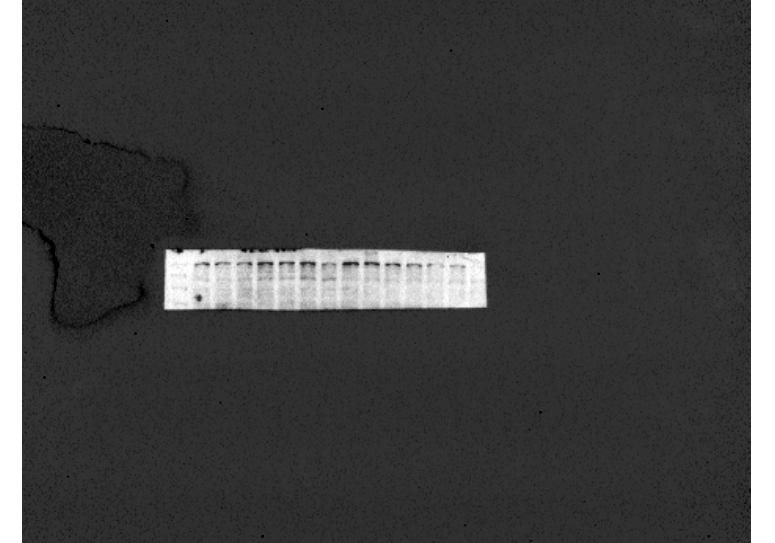


mTOR


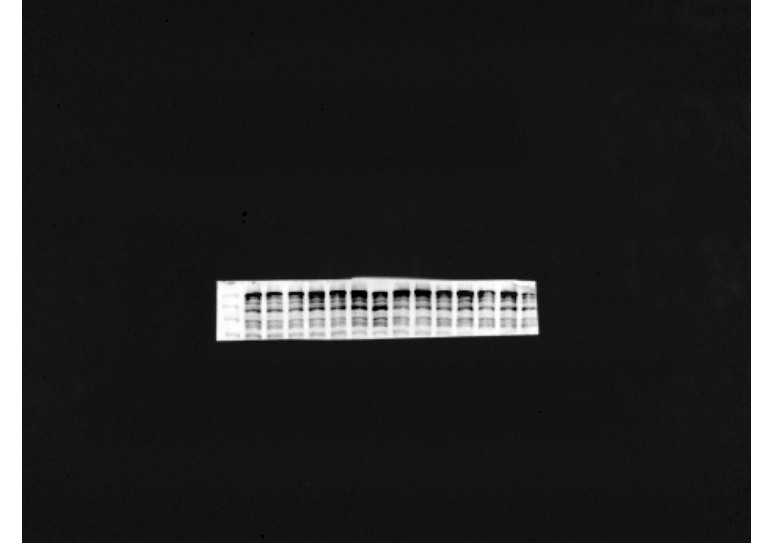


p-P70S6K


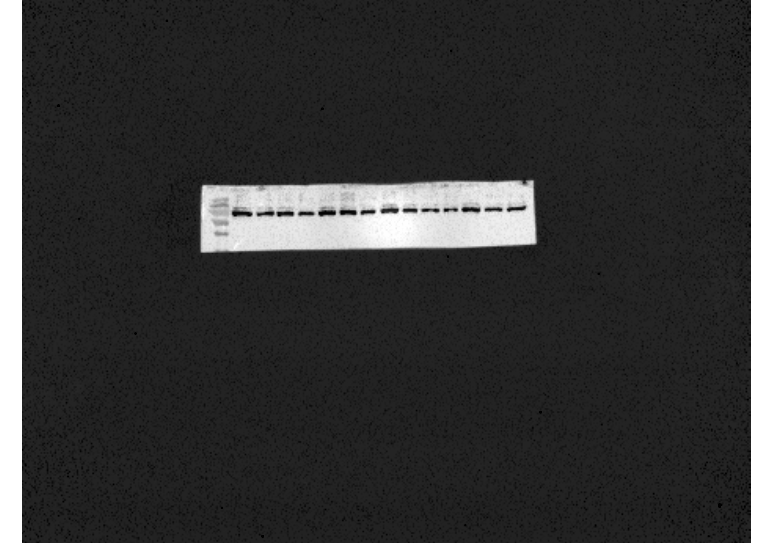


P70S6K


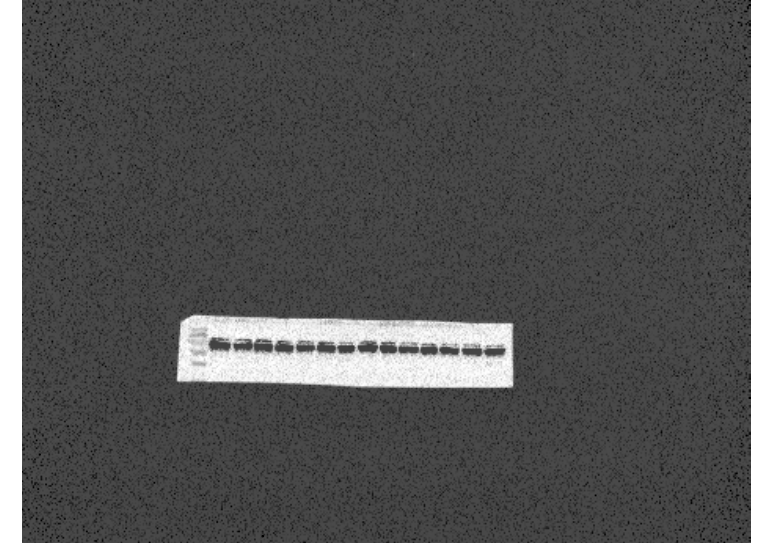


p-STAT5


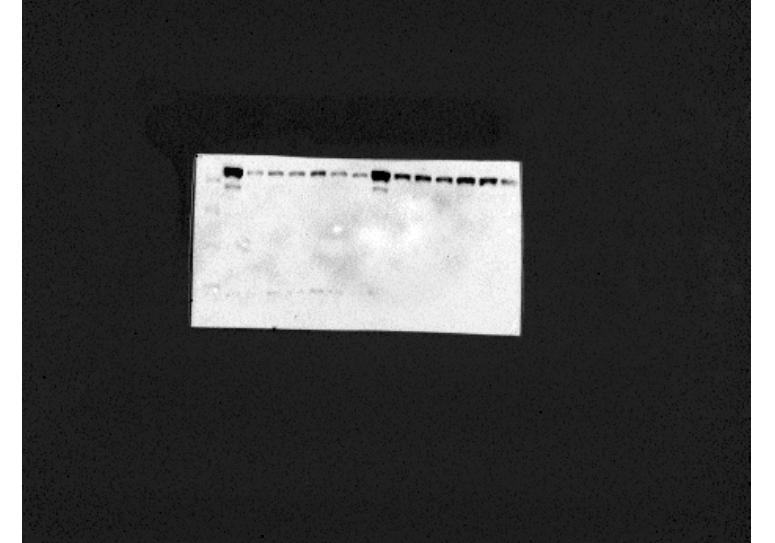


STAT5


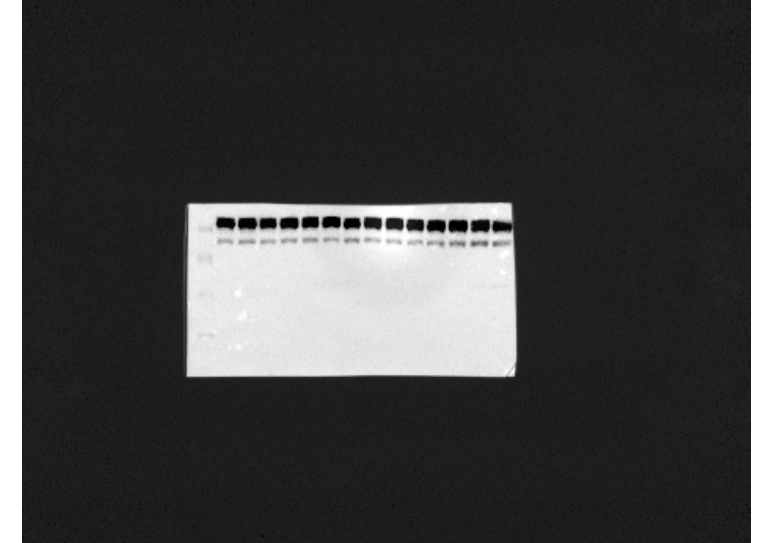


p-AKT


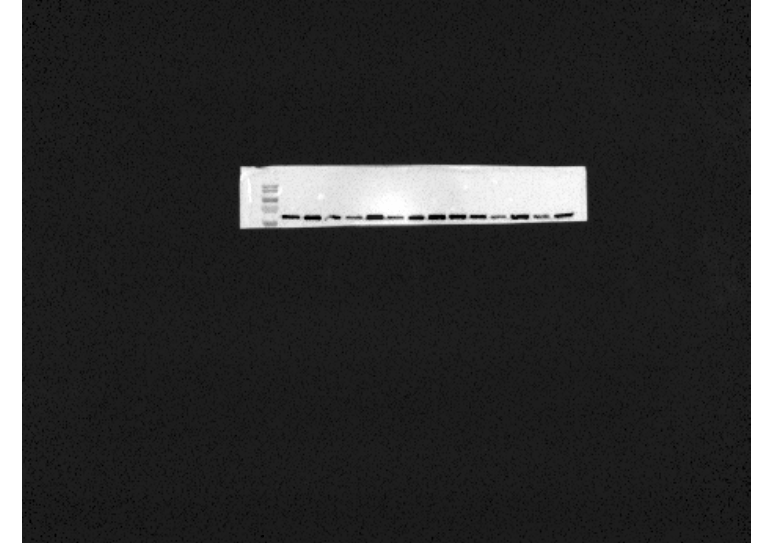


AKT


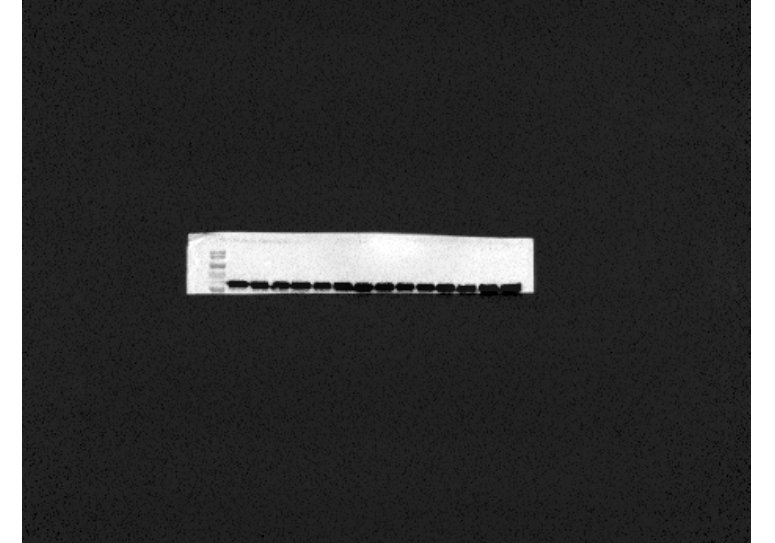


p62


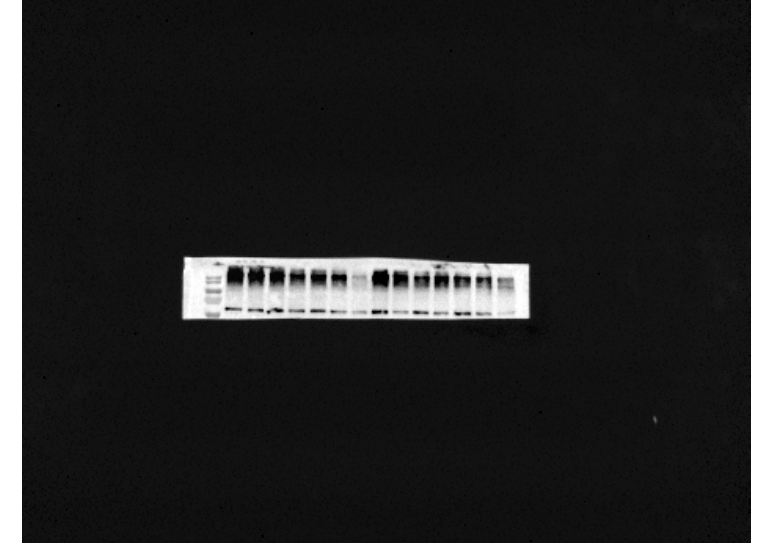


LC3BI/II


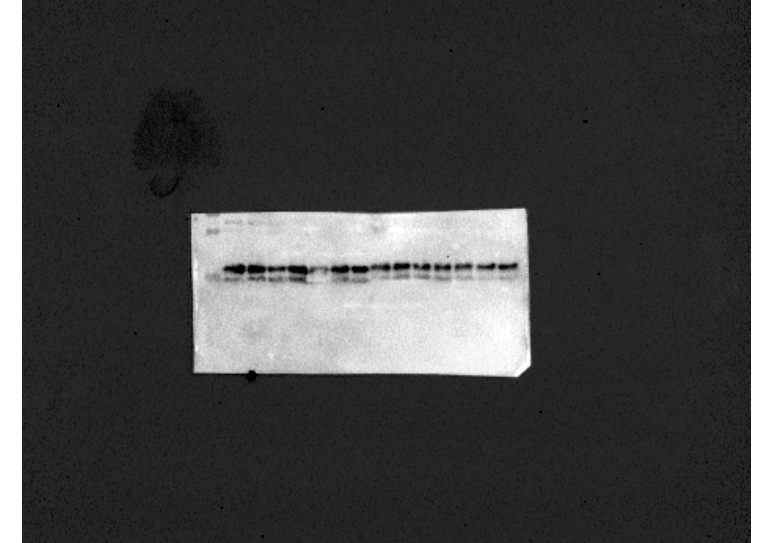


p-ULK1


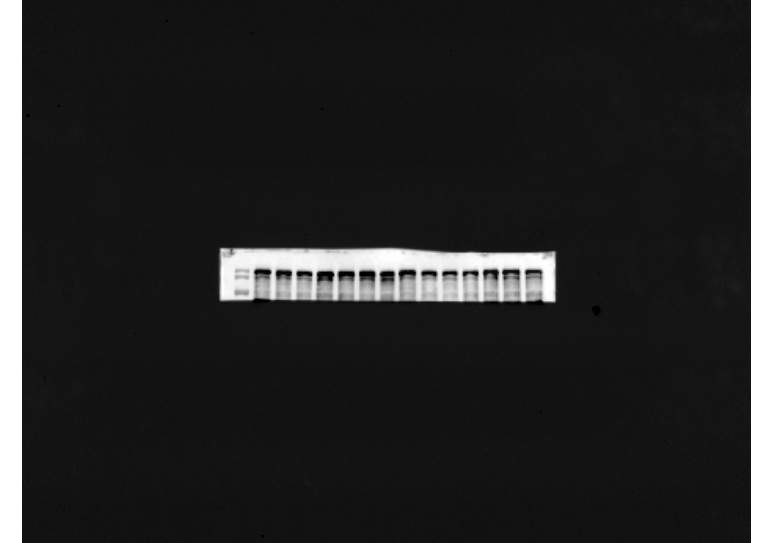


ULK1


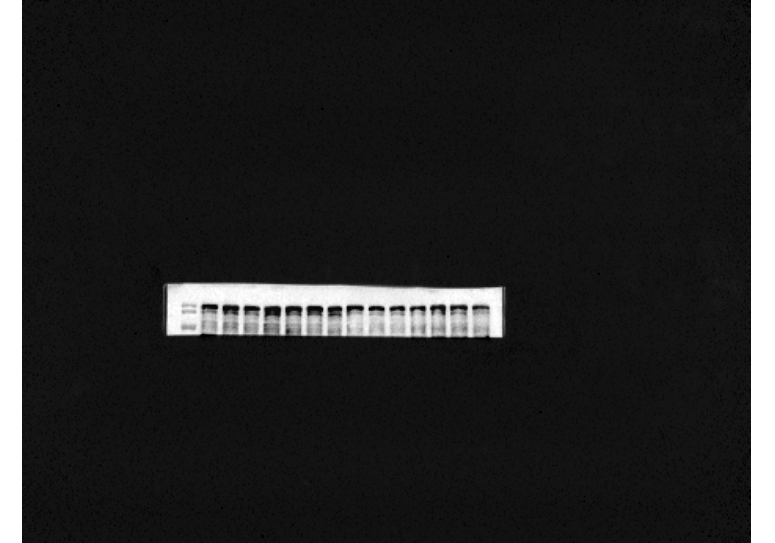


β -Actin from p-mTOR, mTOR, p-STAT5 and STAT5 gel


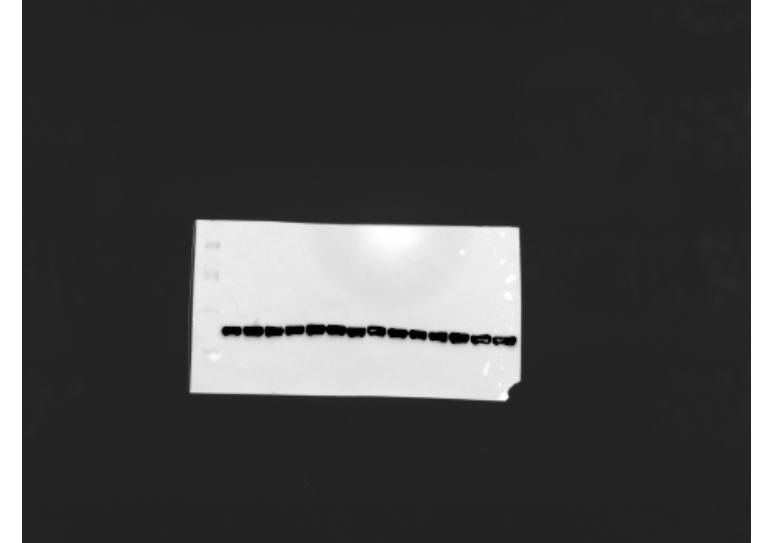


β -Actin from p-P70S6K, P706SK and LC3BI/II gel


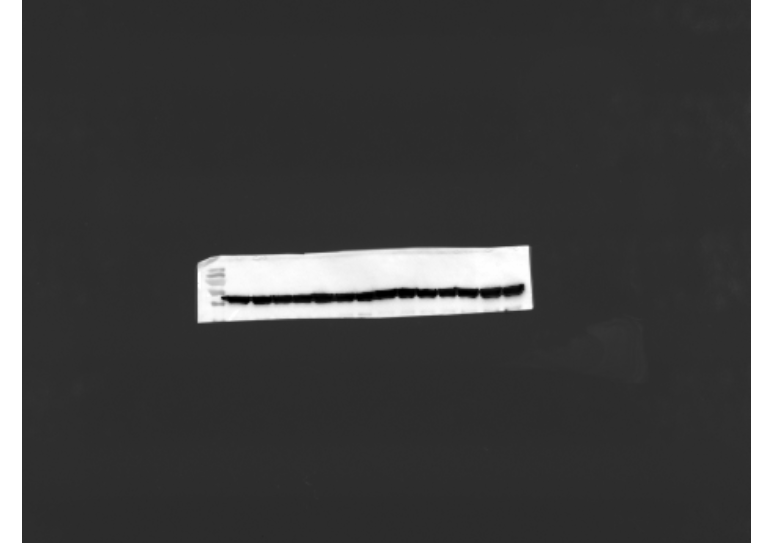


β -Actin from p-AKT, AKT and p62 gel


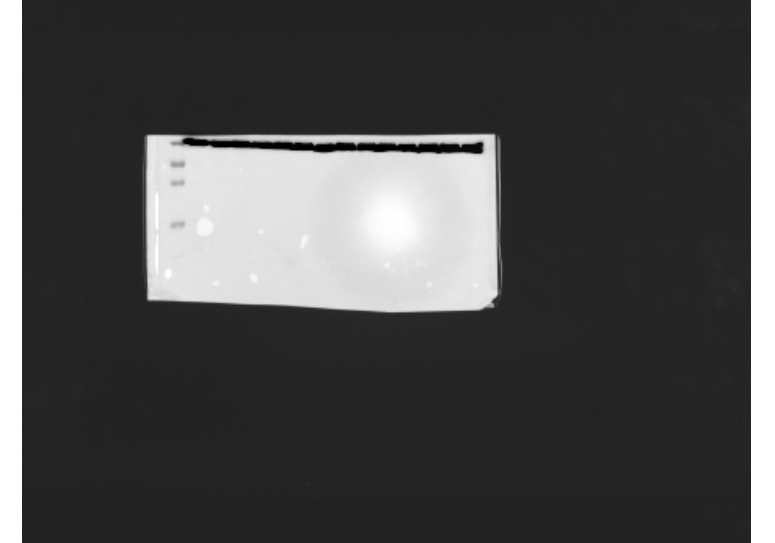


β -Actin from p-ULK1 and ULK1 gel


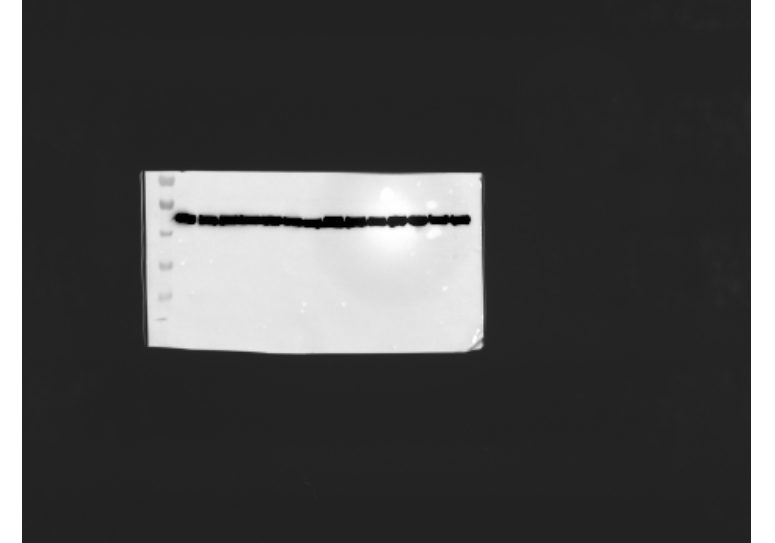


MV4-11 cells

**Ladder – Midostaurin 0h – 3h – 6h – 9h – 12h – 24h – 48h – Quizartinib 0h – 3h – 6h – 9h – 12h – 24h – 48h**

p-mTOR


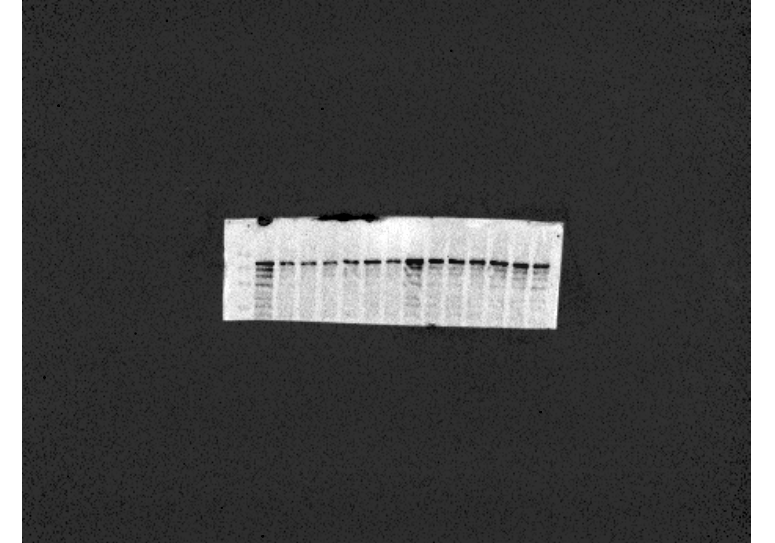


mTOR


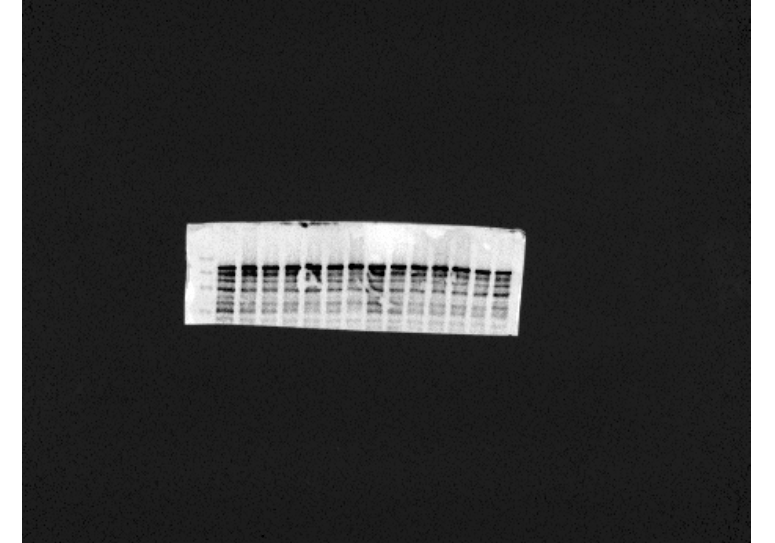


p-P70S6K


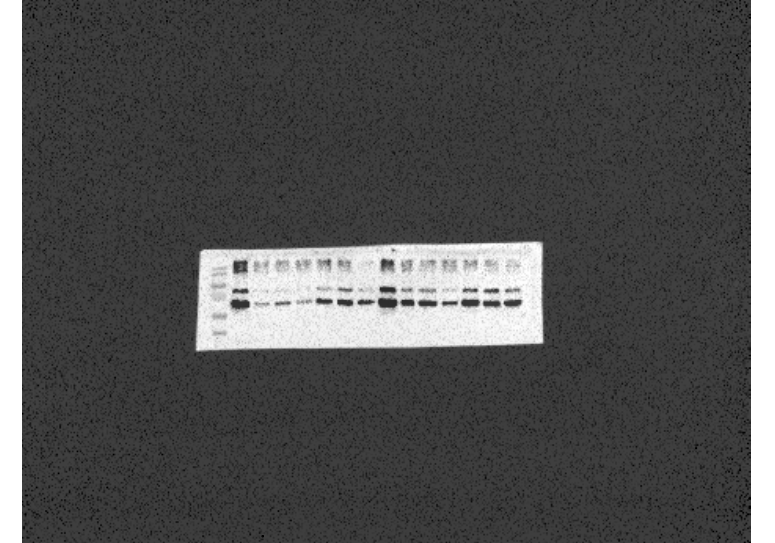


P70S6K


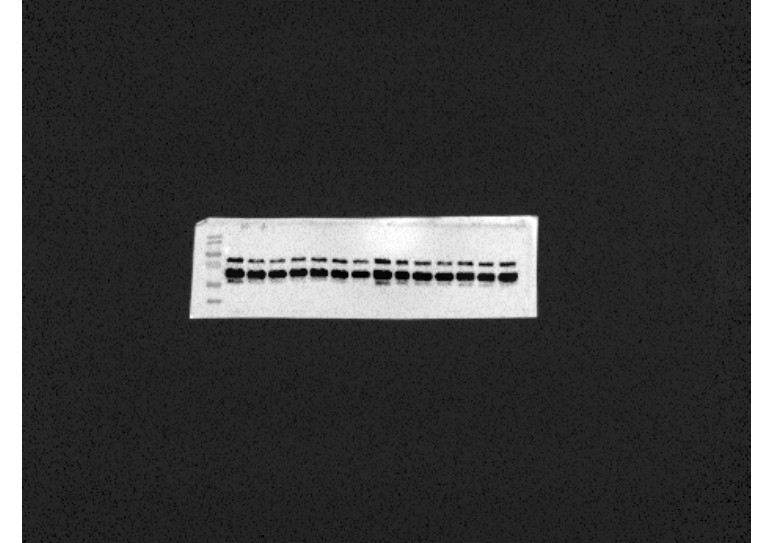


p-STAT5


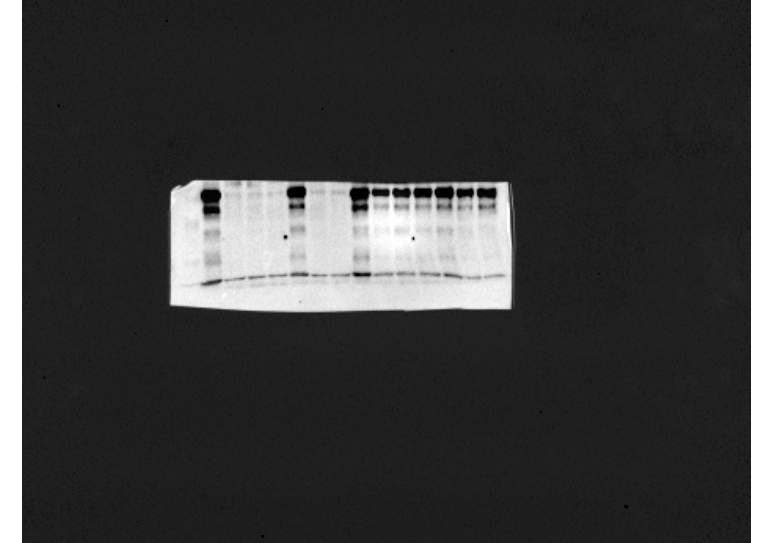


STAT5


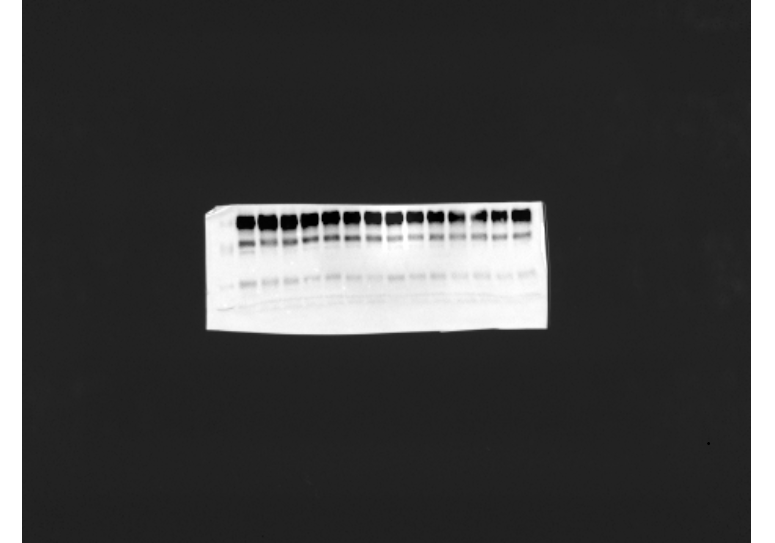


P-AKT


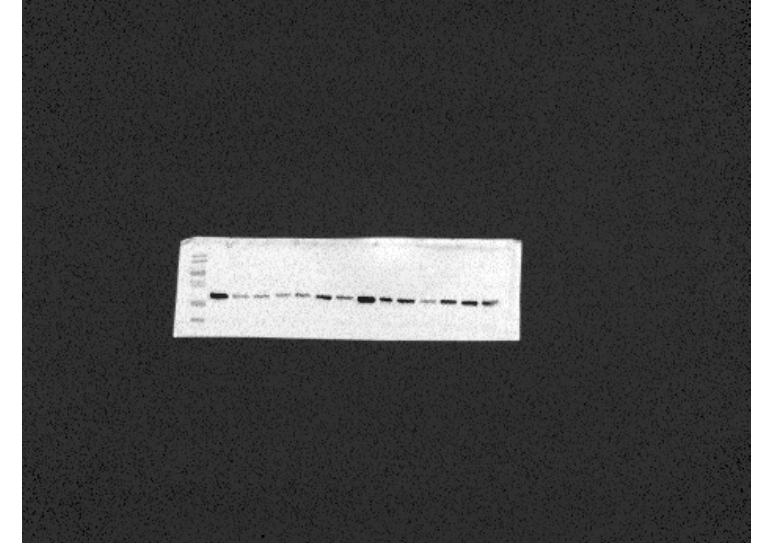


AKT


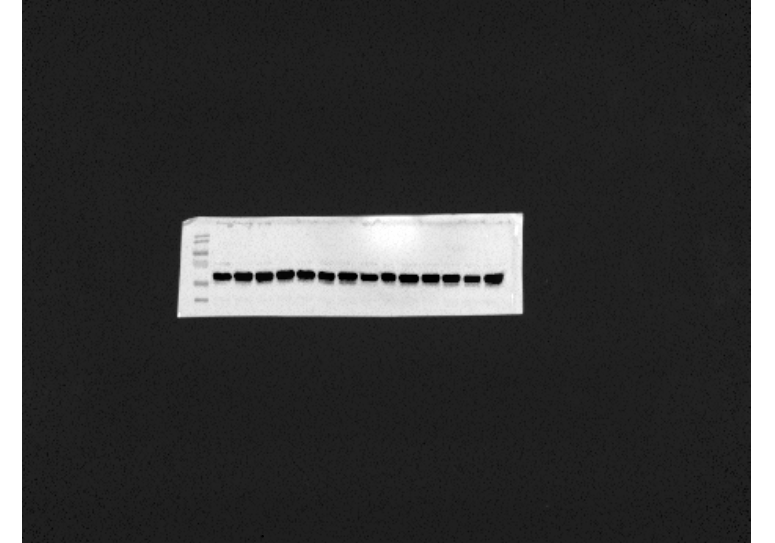


p62


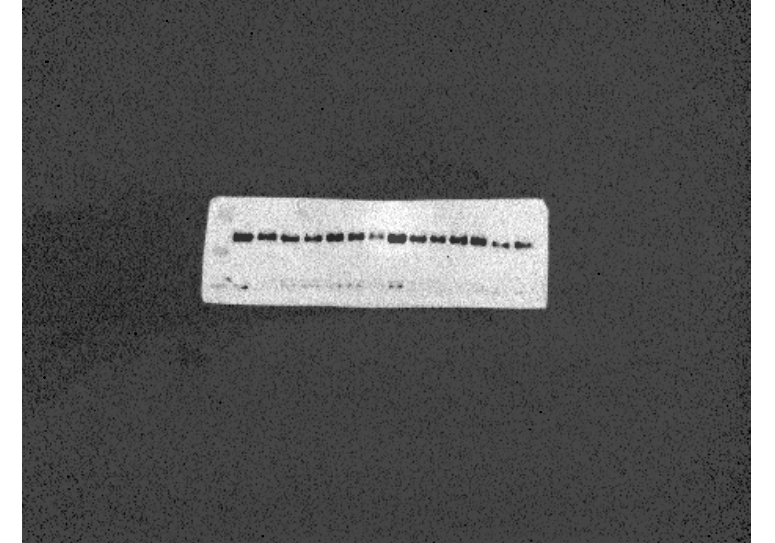


LC3BI/II


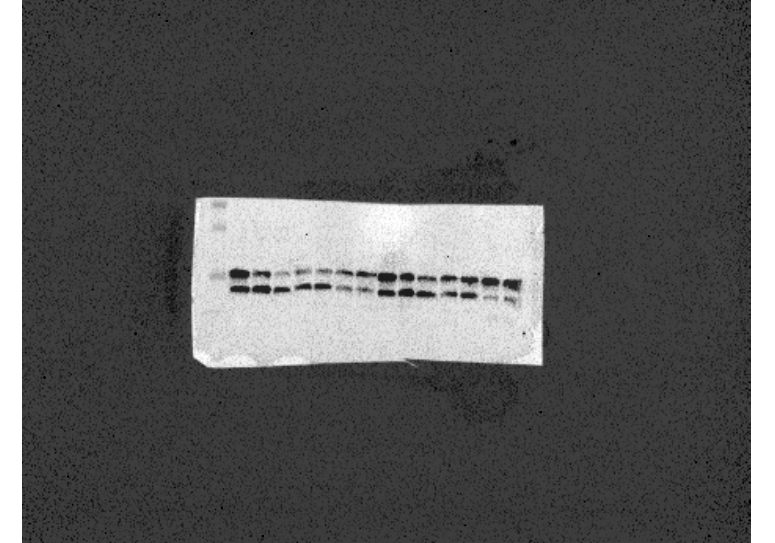


p-ULK1


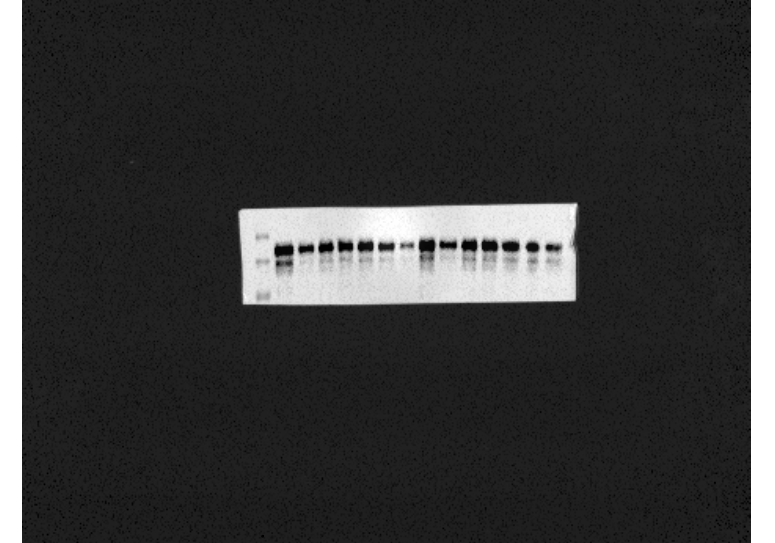


ULK1


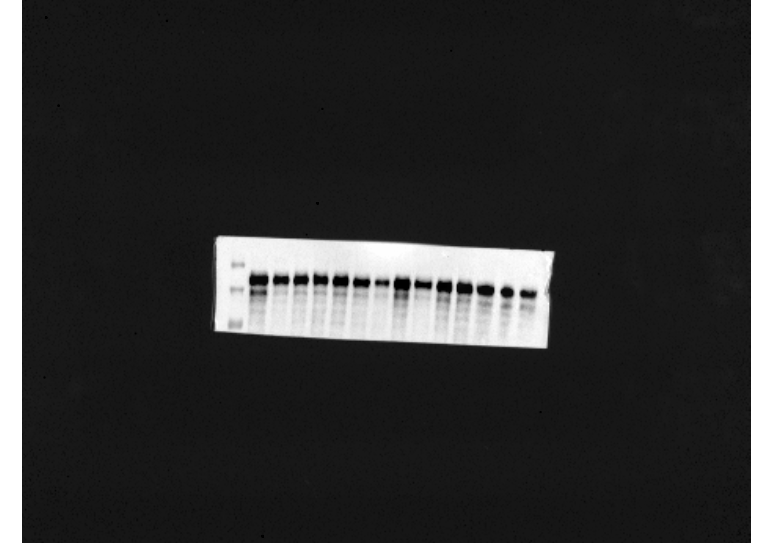


β -Actin from p-mTOR and mTOR gel


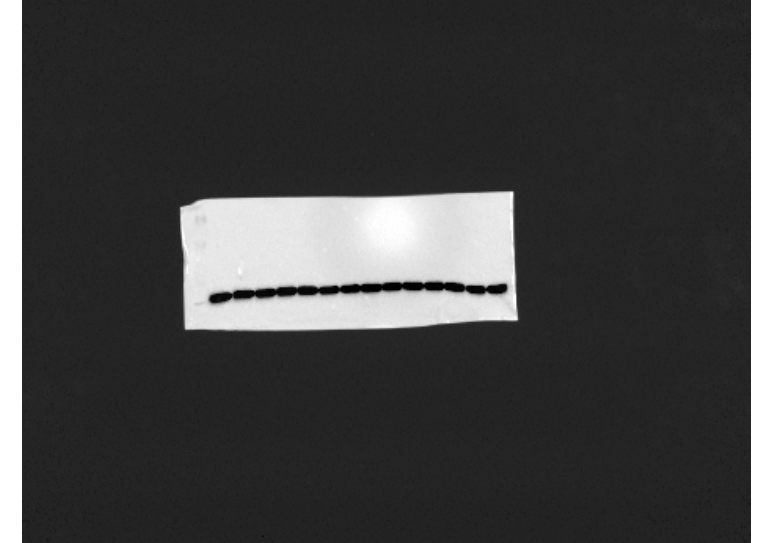


β -Actin from p-P70S6K, P70S6K, p-AKT, AKT and LC3BI/II


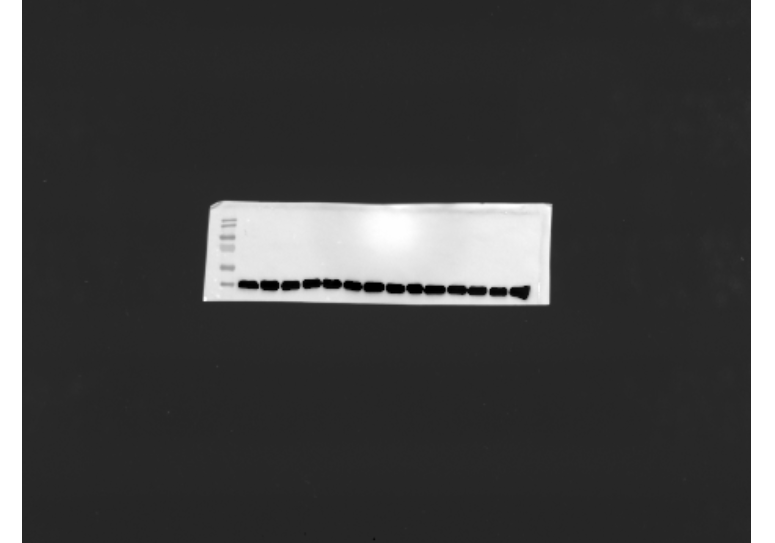


β -Actin from p-STAT5 and STAT5


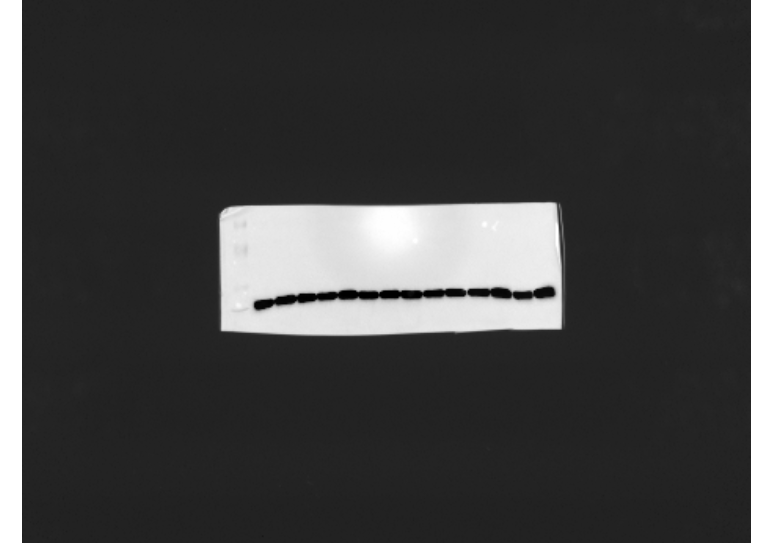


β -Actin from p62 gel


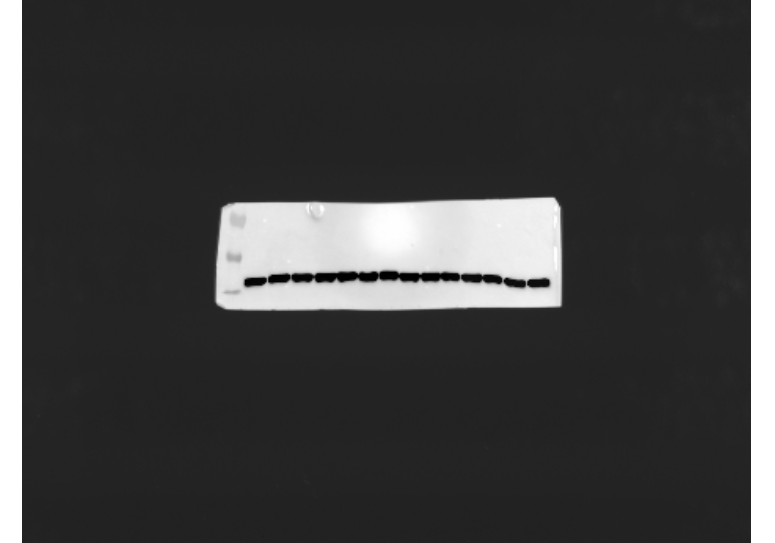


β -Actin from p-ULK1 and ULK1 gel


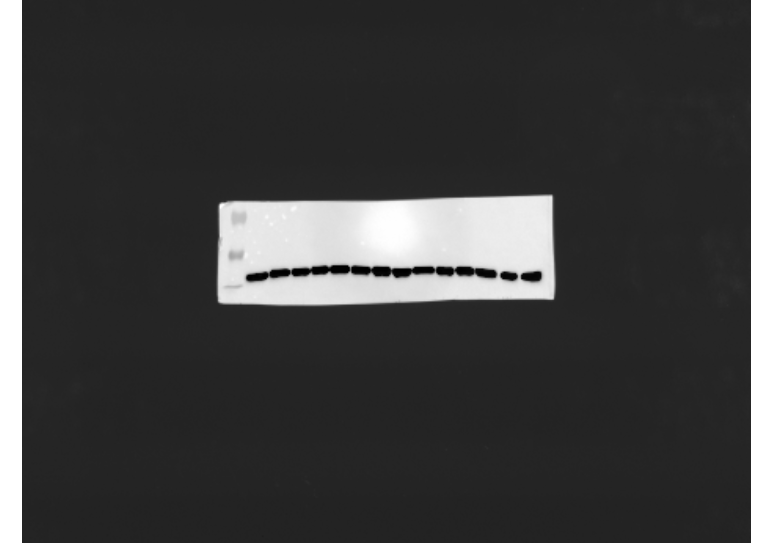


**Full and uncropped Western Blots corresponding to GENE SILENCING EXPERIMENTS ilustrated in the Figure 4A of the manuscript.**

MOLM13 CELLS shRNA

**Ladder - Parental MOLM13 cells - MOLM13 shCONTROL - MOLM13 shATG5**

ATG5


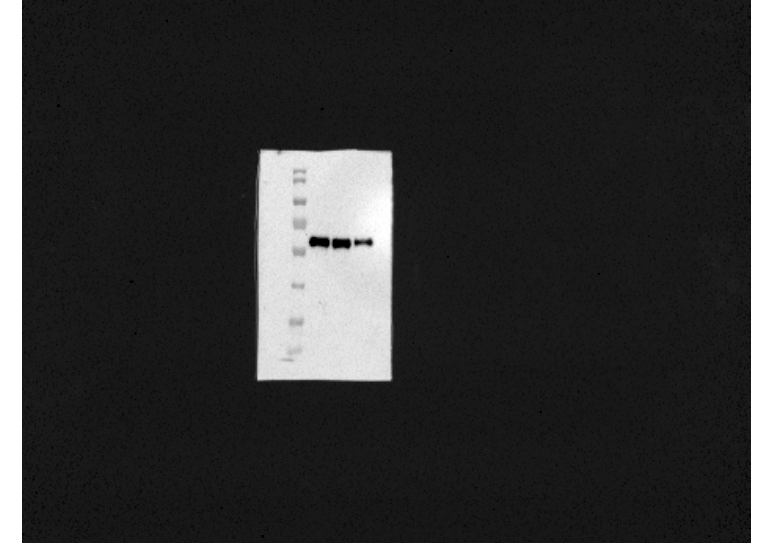


β -Actin from ATG5 gel


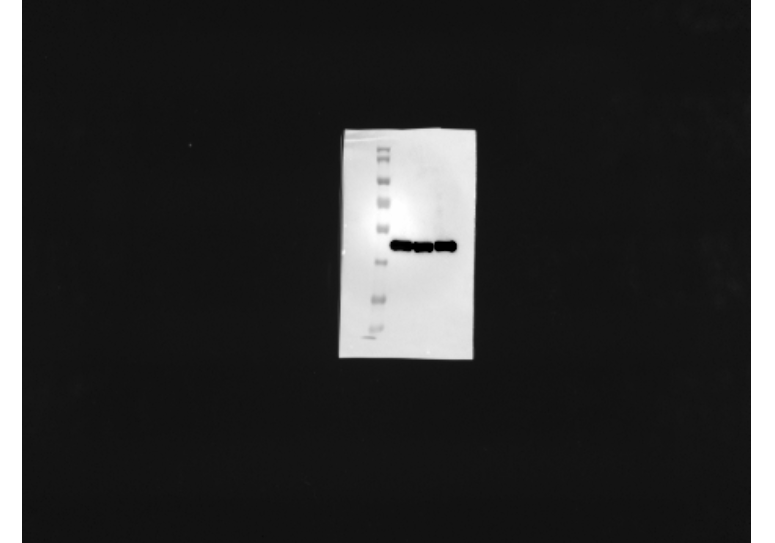


MOLM13 CELLS shRNA

**Ladder - Parental MOLM13 cells - MOLM13 shCONTROL - MOLM13 shATG7**

ATG7


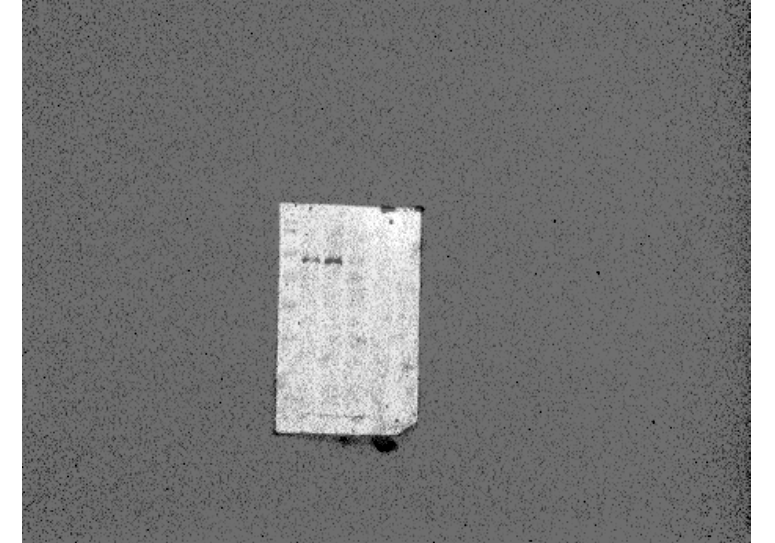


β -Actin from ATG7 gel


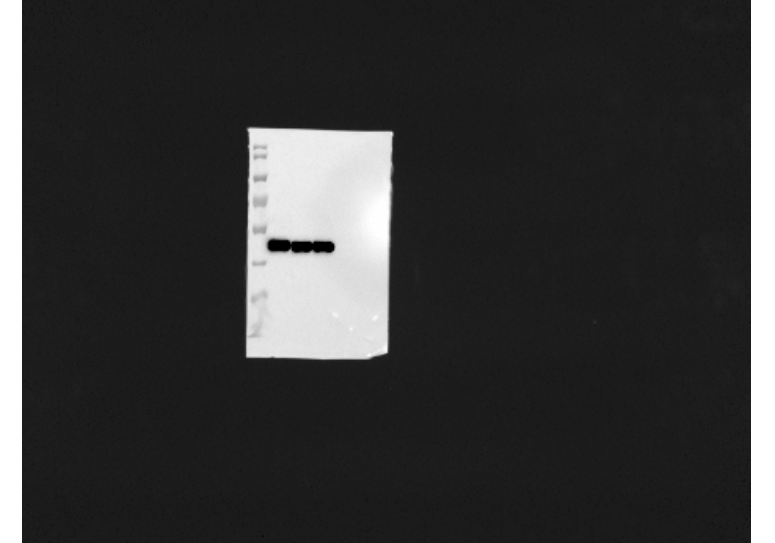


**Full and uncropped Western Blots corresponding to COMBINED EXPERIMENTS (FLT3 inhibitors + Autophagy inhibitors) ilustrated in the Figure 5A of the manuscript.**

MOLM13 cells

**Ladder – Vehicle – Midostaurin 12.5 nM – Midostaurin 12.5 nM + Chloroquine 5 µM – Chloroquine 5 µM - Vehicle - Midostaurin 12.5 nM - Midostaurin 12.5 nM + Bafilomycin 2.5 nM - Bafilomycin 2.5 nM – Vehicle - Midostaurin 12.5 nM - Midostaurin 12.5 nM + ROC-325 1µM - ROC-325 1µM**

ATG7


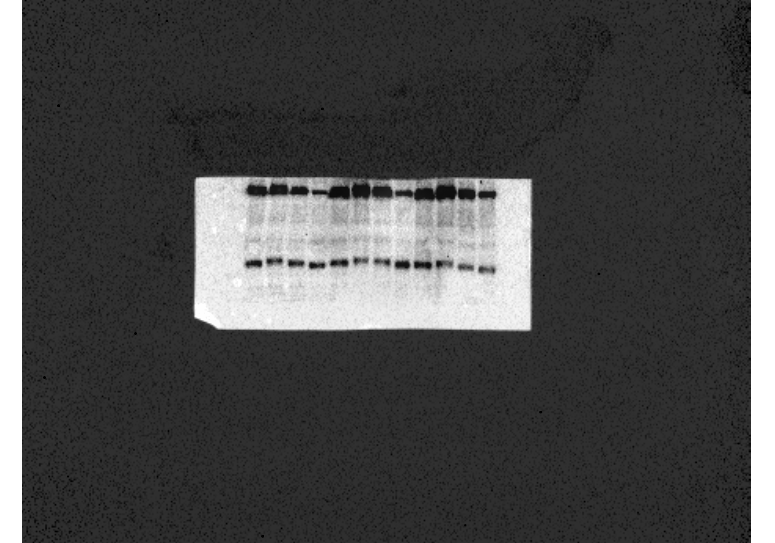


p-mTOR


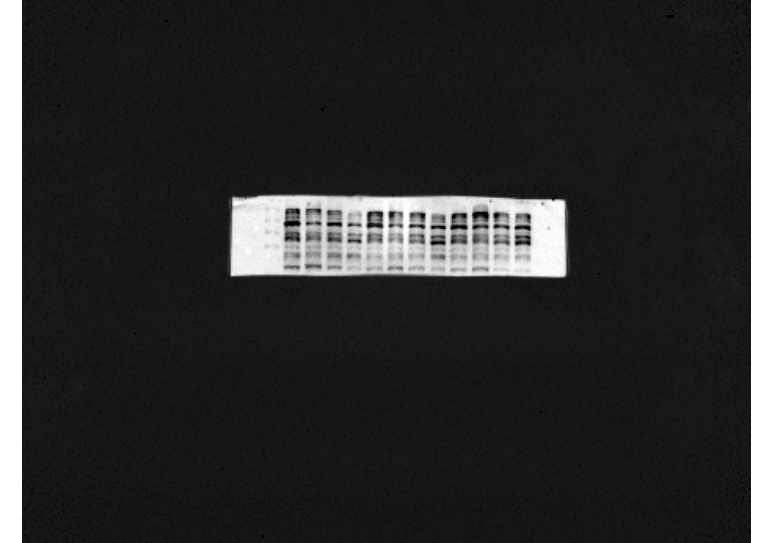


mTOR


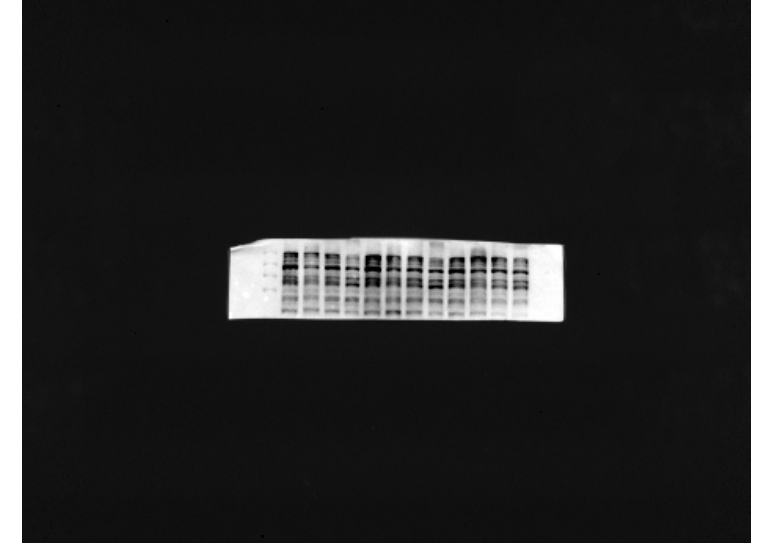


p-STAT5


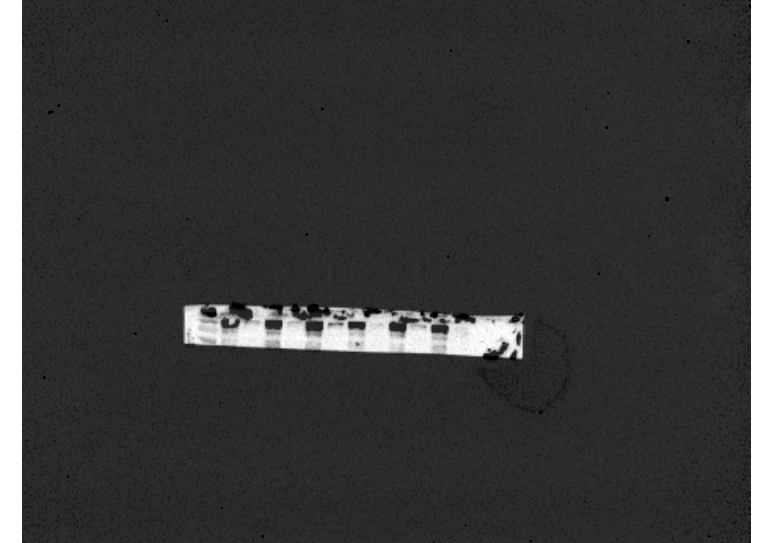


STAT5


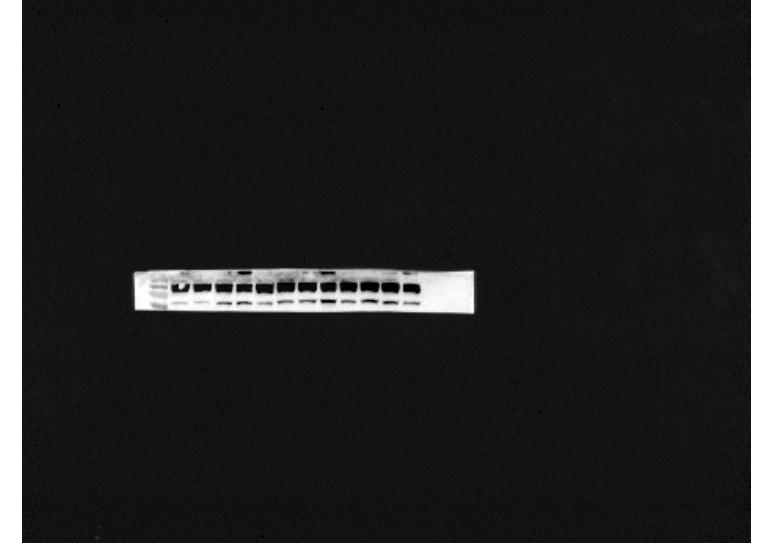


Caspase3


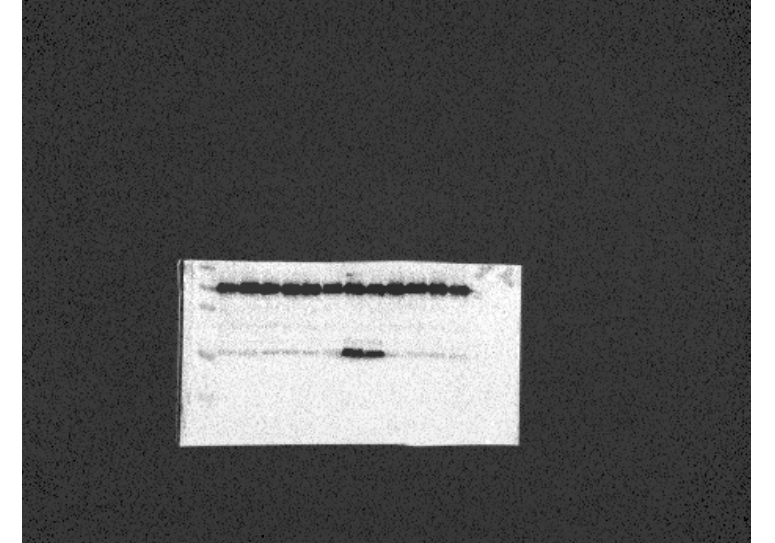


LC3BI/II


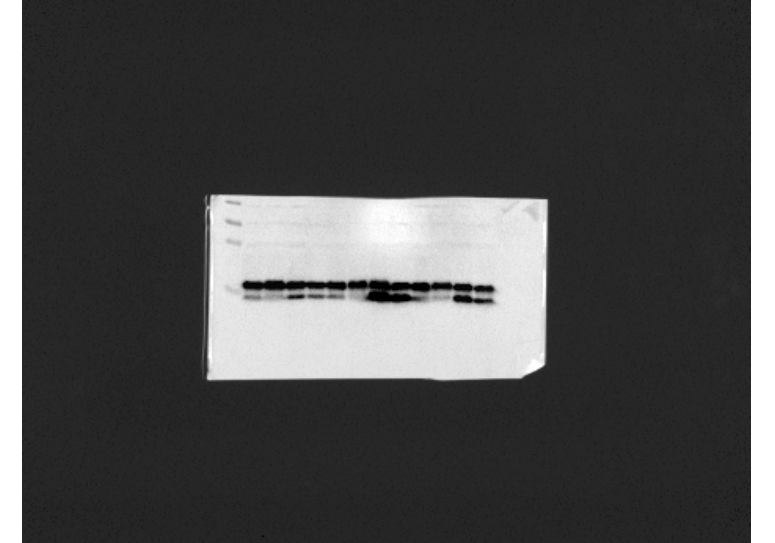


β -Actin from ATG7, p-mTOR and mTOR gel


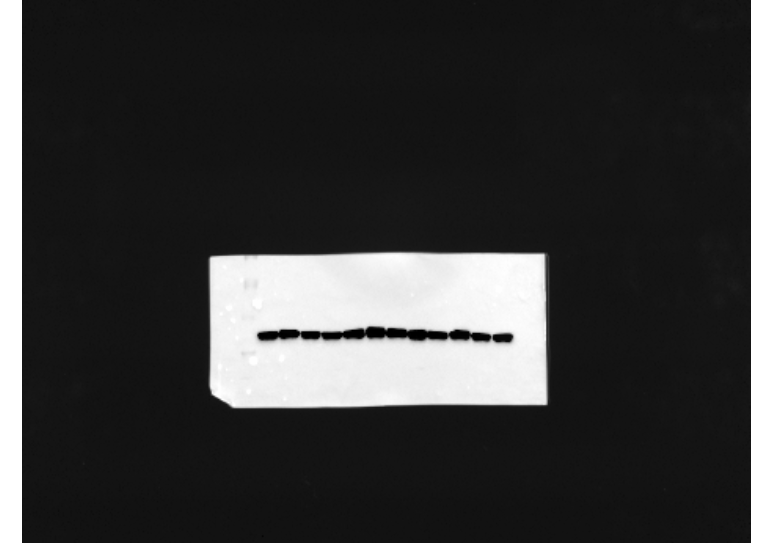


β -Actin from p-STAT5, STAT5 gel


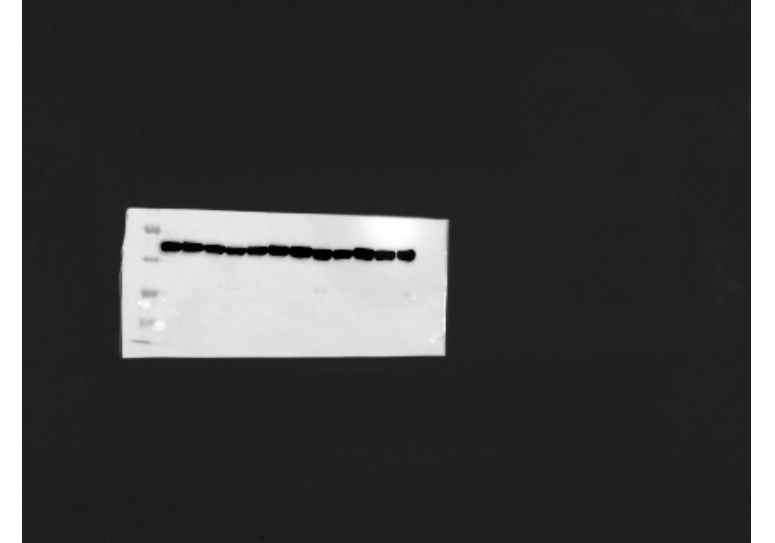


β -Actin from Caspase3 and LC3BI/II gel


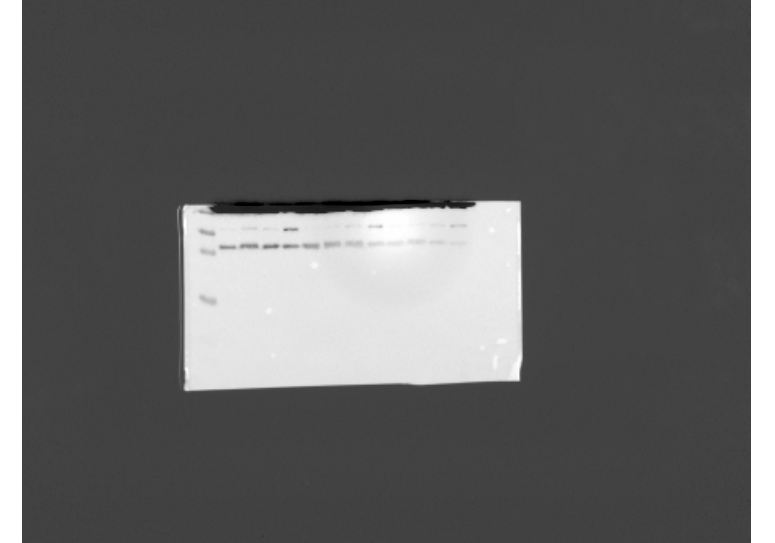


MOLM13 cells

**Ladder – Vehicle – Quizartinib 1.25 nM – Quizartinib 1.25 nM + Chloroquine 5 µM – Chloroquine 5 µM - Vehicle - Quizartinib 1.25 nM - Quizartinib 1.25 nM + Bafilomycin 2.5 nM - Bafilomycin 2.5 nM – Vehicle - Quizartinib 1.25 nM - Quizartinib 1.25 nM + ROC-325 1µM - ROC-325 1µM**

ATG7


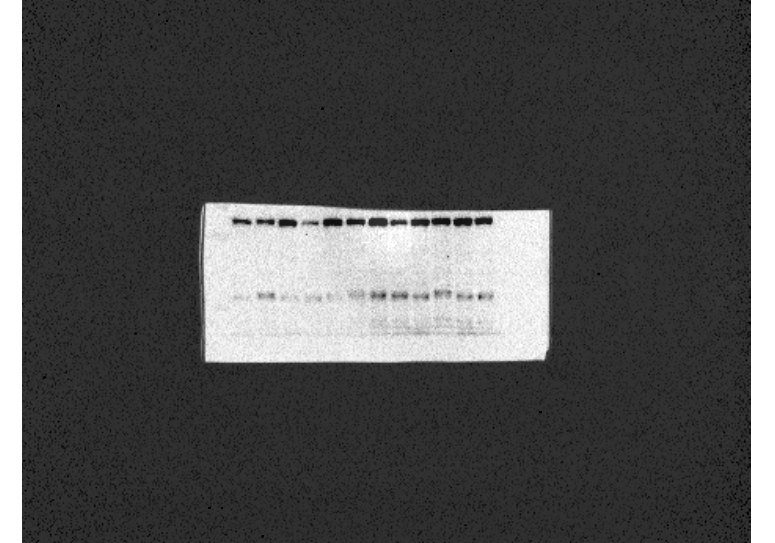


p-mTOR


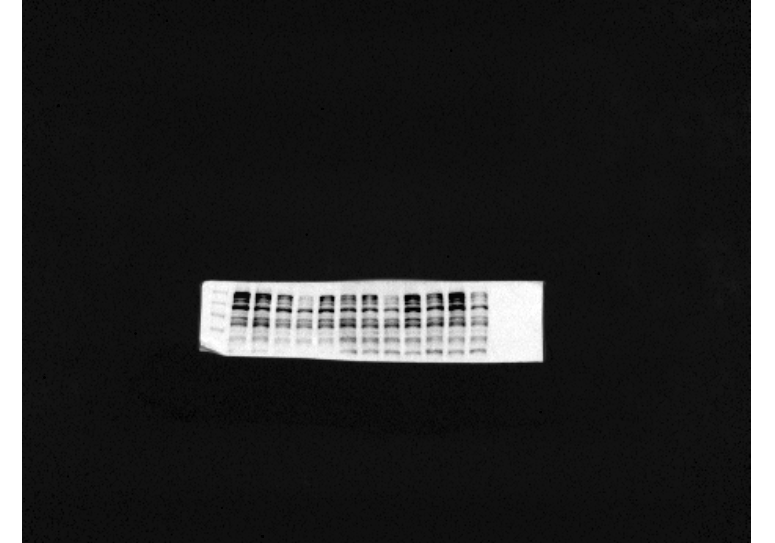


mTOR


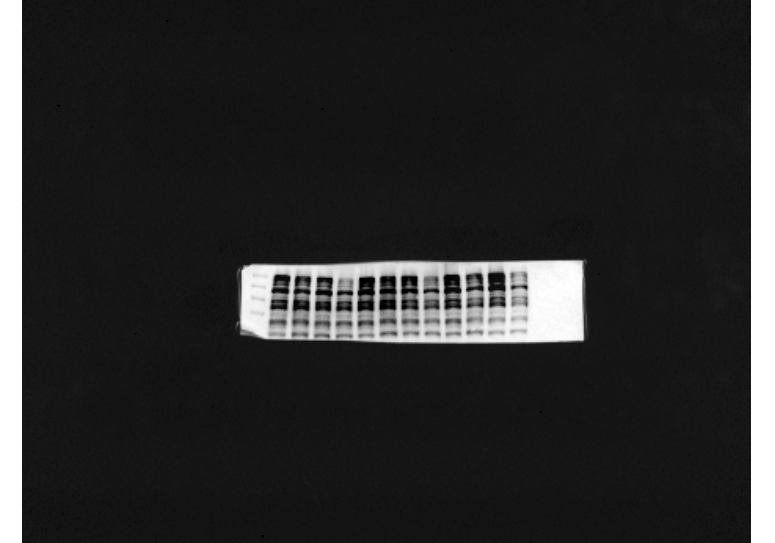


p-STAT5


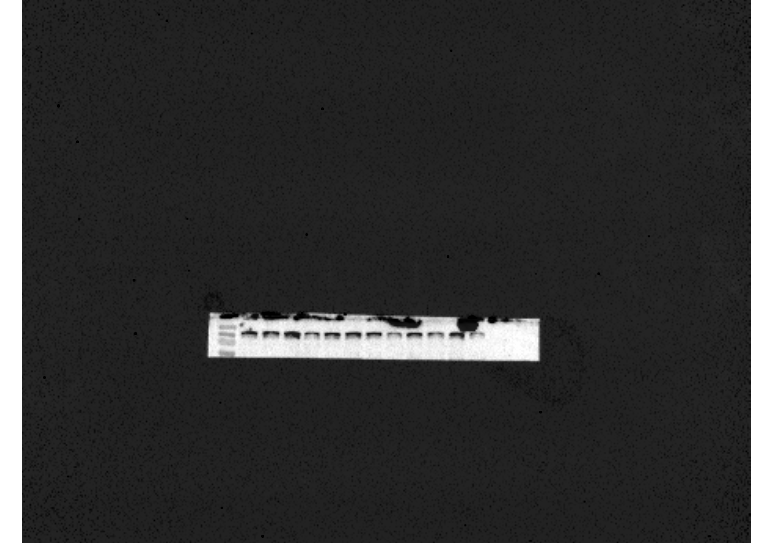


STAT5


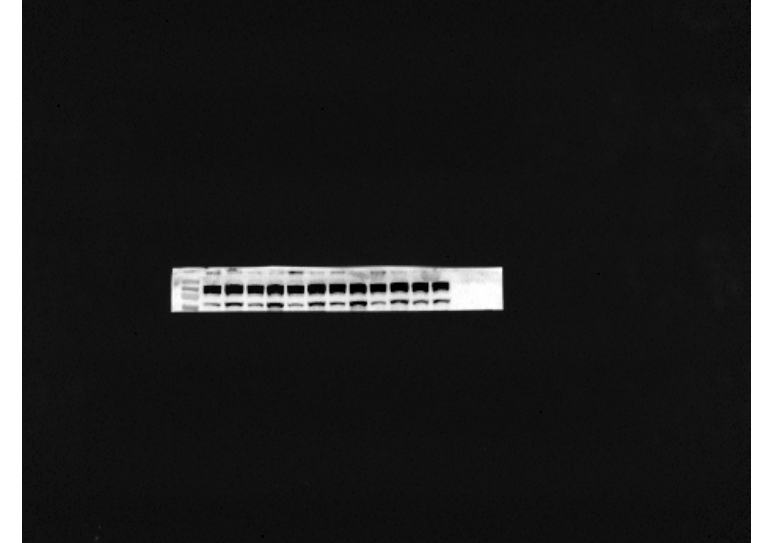


Caspase3


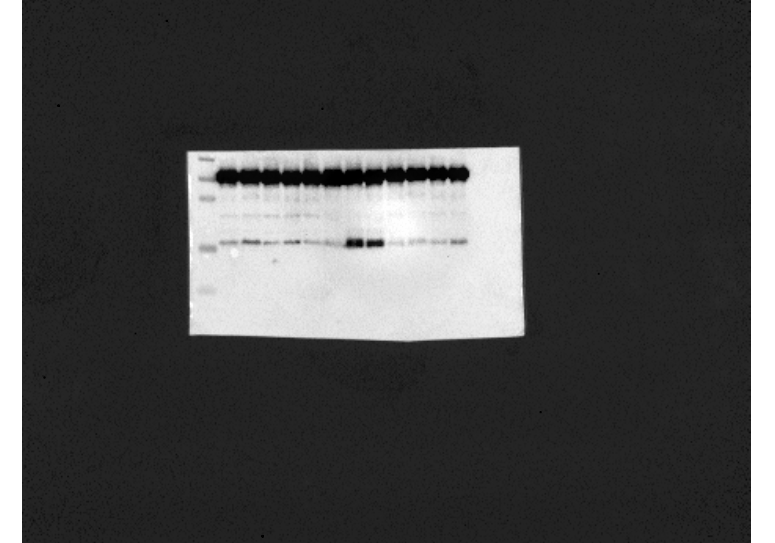


LC3BI/II


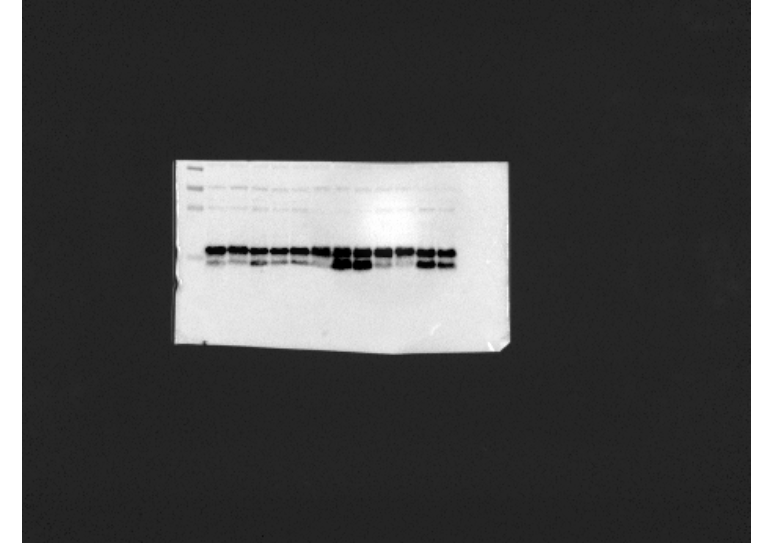


β -Actin from ATG7, p-mTOR and mTOR gel


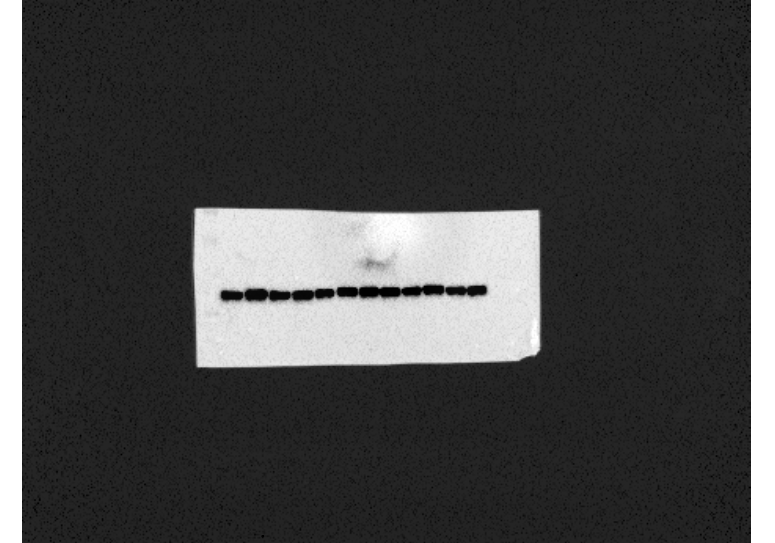


β -Actin from p-STAT5, STAT5, Caspase3 and LC3BI/II gel


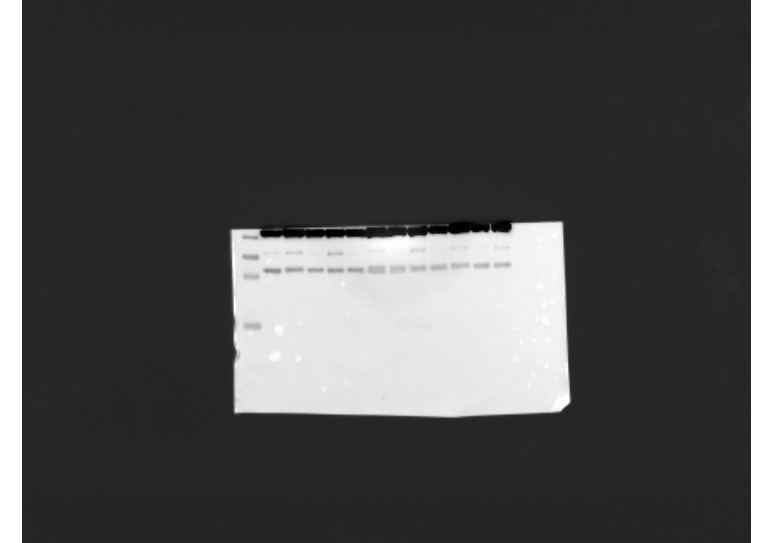


**Full and uncropped Western Blots corresponding to QUIZARTINIB-RESISTANT CELLS EXPERIMENTS ilustrated in the Figure 6E of the manuscript.**

**Ladder – Vehicle (MV4-11 cells) - Quizartinib 10 nM (MV4-11 cells ) – Quizartinib 10 nM + Chloroquine 10 µM (MV4-11 cells) - Chloroquine 10 µM (MV4-11 cells) – Empty lane - Ladder - Vehicle (MV4-11QR cells) - Quizartinib 10 nM (MV4-11QR cells) - Quizartinib 10 nM + Chloroquine 10 µM (MV4-11QR cells) - Chloroquine 10 µM (MV4-11QR cells)**

p-P70S6K


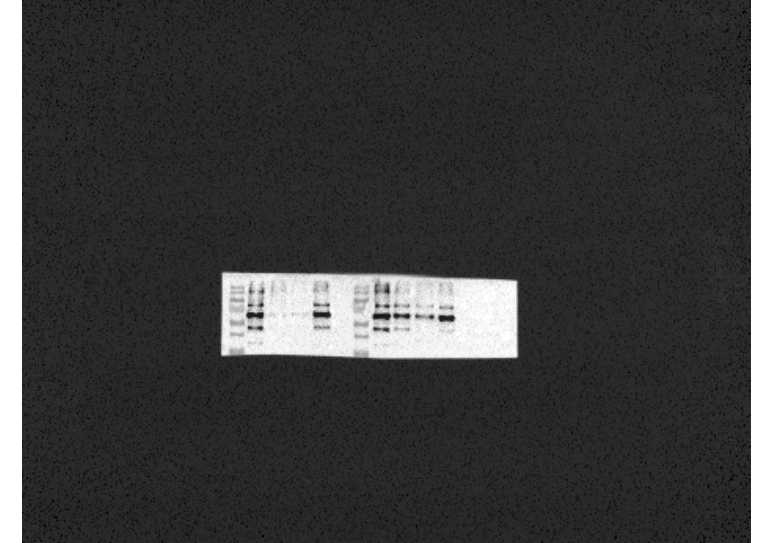


P70S6K


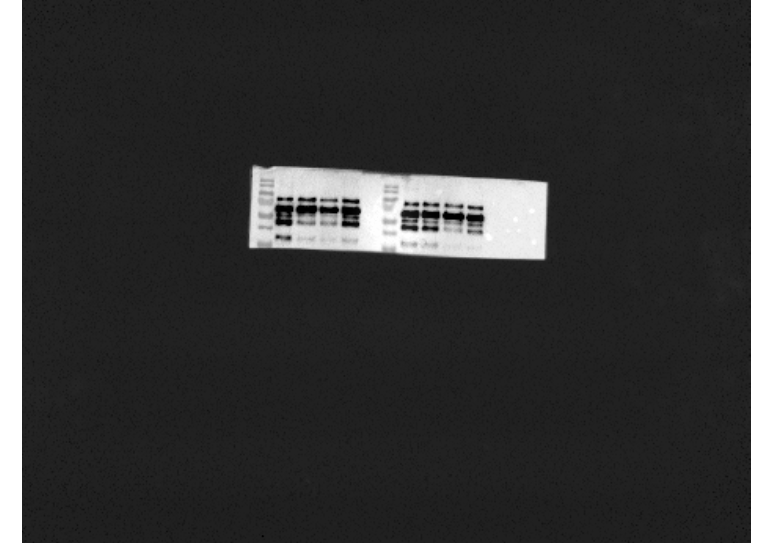


p-STAT5


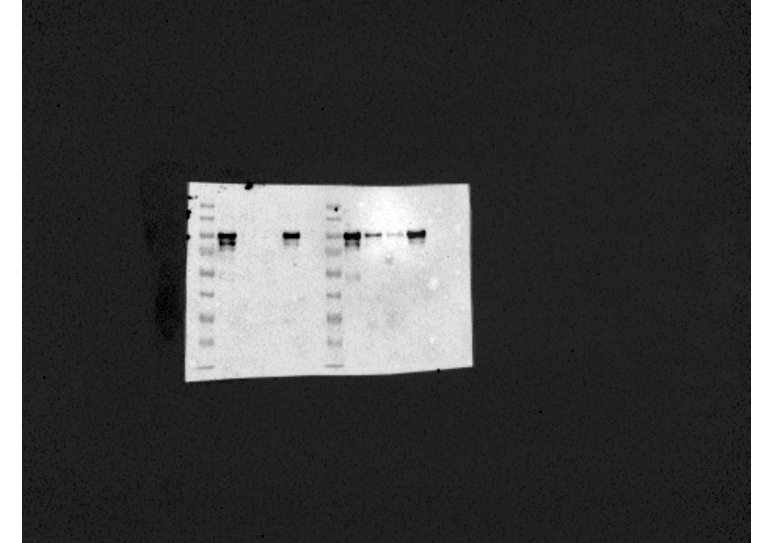


STAT5


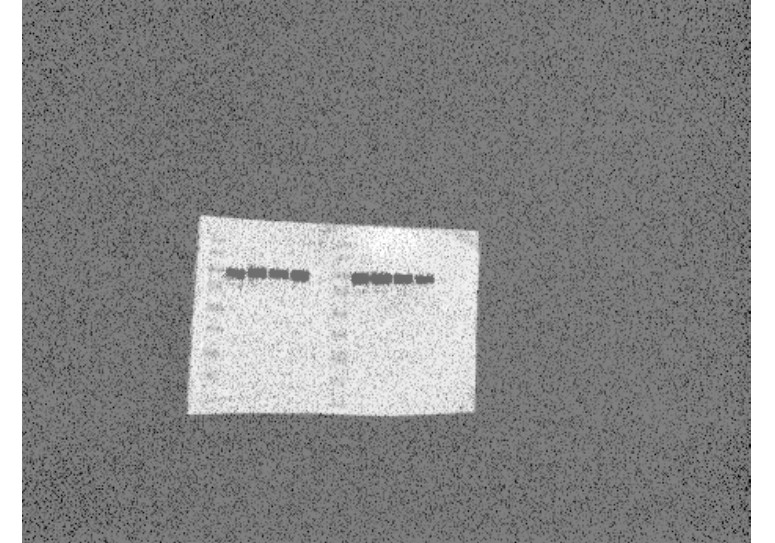


Caspase3


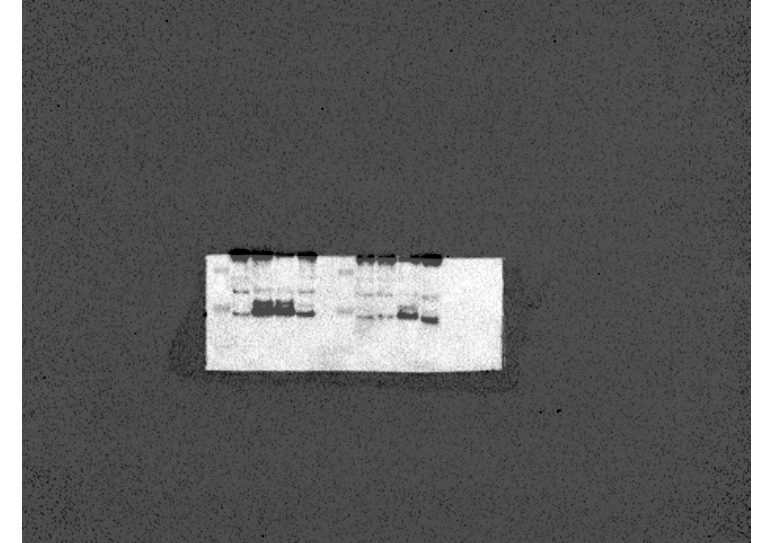


LC3BI/II


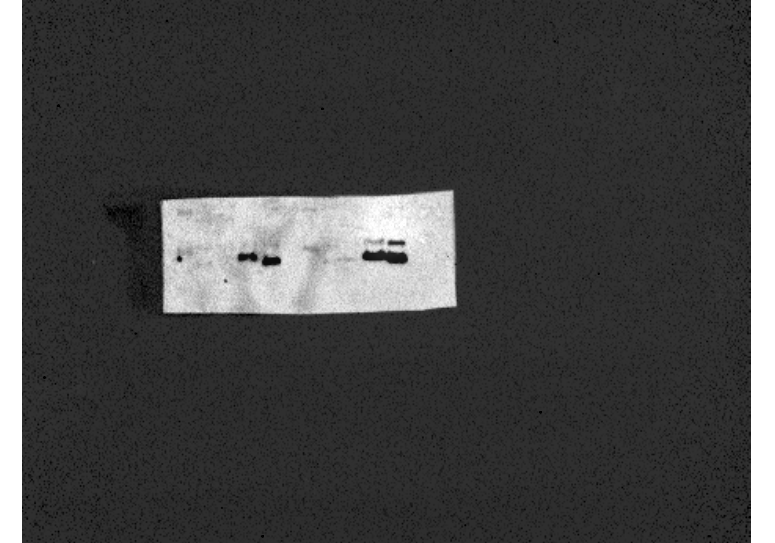


p62


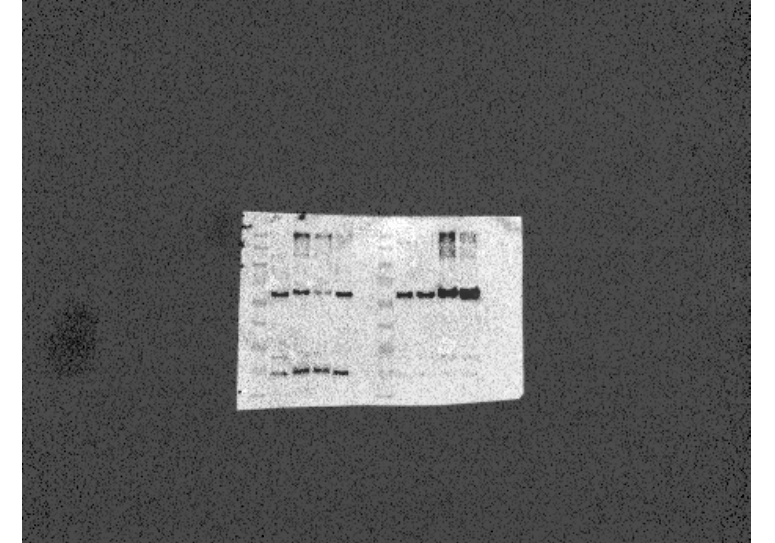


β -Actin from p-P70S6K, P70S6K and Caspase3 gel


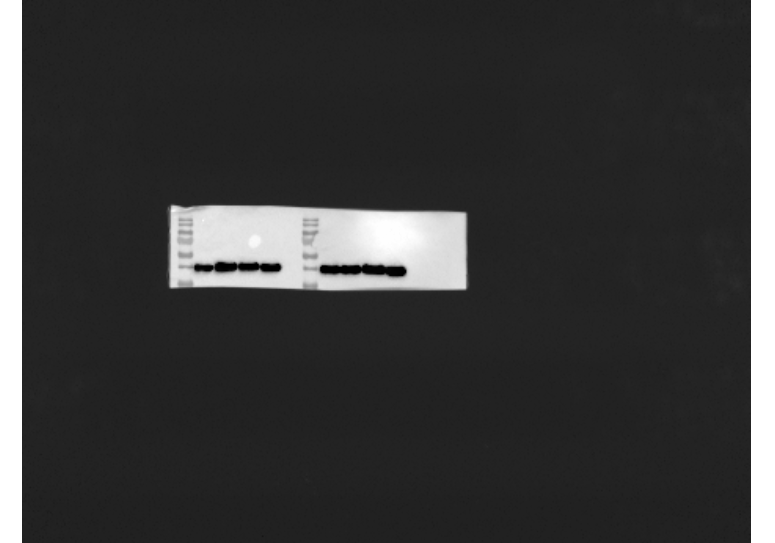


β -Actin from p-STAT5, STAT5 and p62 gel


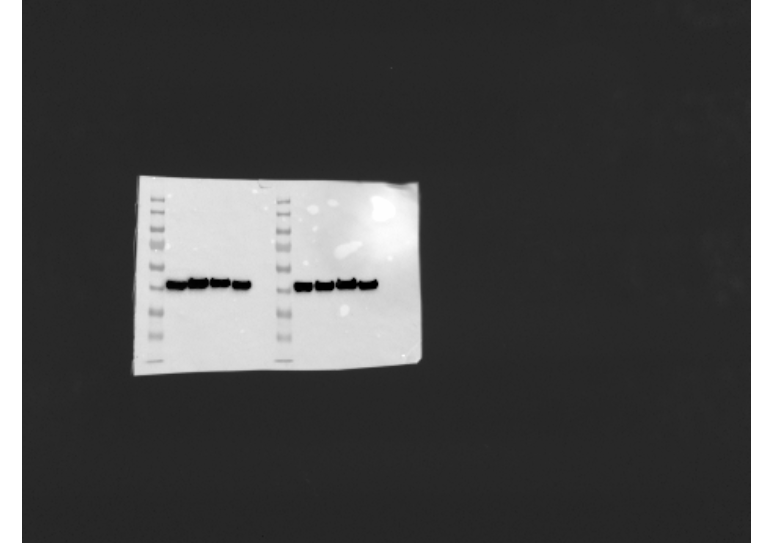


β -Actin from LC3BI/II gel


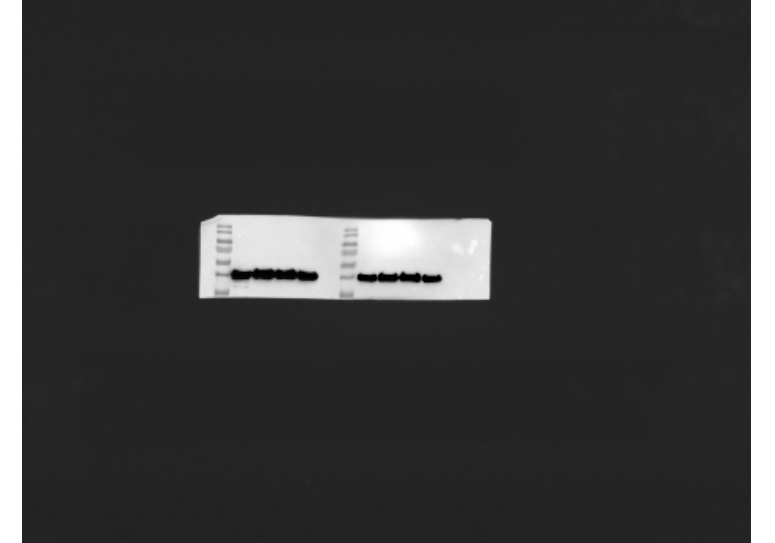

Supplement: Supplementary file 2 — Full and Uncropped Western Blot [file 41420_2026_3037_MOESM2_ESM.docx]
